# Building Better Batteries in the Solid State: A Review

**DOI:** 10.3390/ma12233892

**Published:** 2019-11-25

**Authors:** Alain Mauger, Christian M. Julien, Andrea Paolella, Michel Armand, Karim Zaghib

**Affiliations:** 1 Institut de Minéralogie, de Physique des Matériaux et de Cosmochimie (IMPMC), Sorbonne Université, UMR-CNRS 7590, 4 place Jussieu, 75005 Paris, France; alain.mauger@upmc.fr; 2 Centre of Excellence in Transportation Electrification and Energy Storage (CETEES), Hydro-Québec, 1806, Lionel-Boulet blvd., Varennes, QC J3X 1S1, Canada; paolella.andrea2@ireq.ca; 3 CIC Energigune, Parque Tecnol Alava, 01510 Minano, Spain; michel.armand@gmail.com

**Keywords:** all-solid-state batteries, solid electrolytes, fast-ion conductors, Li-ion batteries, Na-ion batteries, Li-air batteries, Li–S batteries, polymers, ceramics

## Abstract

Most of the current commercialized lithium batteries employ liquid electrolytes, despite their vulnerability to battery fire hazards, because they avoid the formation of dendrites on the anode side, which is commonly encountered in solid-state batteries. In a review two years ago, we focused on the challenges and issues facing lithium metal for solid-state rechargeable batteries, pointed to the progress made in addressing this drawback, and concluded that a situation could be envisioned where solid-state batteries would again win over liquid batteries for different applications in the near future. However, an additional drawback of solid-state batteries is the lower ionic conductivity of the electrolyte. Therefore, extensive research efforts have been invested in the last few years to overcome this problem, the reward of which has been significant progress. It is the purpose of this review to report these recent works and the state of the art on solid electrolytes. In addition to solid electrolytes *stricto sensu*, there are other electrolytes that are mainly solids, but with some added liquid. In some cases, the amount of liquid added is only on the microliter scale; the addition of liquid is aimed at only improving the contact between a solid-state electrolyte and an electrode, for instance. In some other cases, the amount of liquid is larger, as in the case of gel polymers. It is also an acceptable solution if the amount of liquid is small enough to maintain the safety of the cell; such cases are also considered in this review. Different chemistries are examined, including not only Li-air, Li–O_2_, and Li–S, but also sodium-ion batteries, which are also subject to intensive research. The challenges toward commercialization are also considered.

## 1. Introduction

Intensive efforts have been devoted to the search for high-energy dense lithium batteries that are capable of meeting the demands of the rapidly expanding portable device market and the growing electric vehicle industry. Actually, only the Li-ion technology may represent an option to support EVs and portable devices demand due to the high energy density in comparison with supercapacitors or other hybrid chemistries [1]. Owing to the apparent impossibility to master the Li^0^ electrode with liquid electrolytes (see reference [2] and other references herein for a summary of the industrial attempts), the early 1990s saw the development of lithium-ion batteries, in which the two electrodes are of intercalation type without involving metal nucleation. The limits of liquid electrolyte systems seem close to being reached, with the safety, lifetime, and energy density all reaching a plateau. All-solid-state batteries display many advantages compared to lithium-ion batteries. They are not only inherently safer owing to the lack of flammable organic components, but also exhibit potential for a dramatic improvement in the energy density [3,4,5]. Because of the reduced conductivity of polymers, some liquid electrolytes may be added, forming the so-called gel polymer electrolytes (GPEs). A few of them of particular interest that combine the advantages of both liquid electrolytes and solid polymer electrolytes (SPEs) will be considered here [6], even though the low mechanical strength and poor interfacial properties are still obstacles to their practical use [7]. The use of lithium metal as the anode material can increase the volumetric energy density by up to 70% with respect to that of lithium-ion batteries equipped with graphite anodes [8] but raises the problem of its contact with solid electrolytes [9]. Bipolar stacking is facilitated with single cells that are connected in series by a lithium-ion isolating layer. This can be used to increase the voltage of a battery cell and reduce the number of current collectors in the cell stack, as well as to optimize the packaging design. The progress in industrialization of all-solid-state lithium batteries has stagnated since 2011 [10], although advances have been reported in such batteries through research and development [11]. For these reasons, many research efforts have been devoted to the improvement of solid electrolytes in terms of their conductivity, mechanical properties, and contact with electrodes, which is an important parameter, as accommodating the volume change during cycling is more difficult with non-compliant solid electrolytes than with liquid electrolytes [12], and the investigation of polymer electrolytes has been steadily increasing ever since [13,14]. A lithium-metal polymer cell with a LiFePO_4_ (LFP) counter-electrode and a poly(ethylene oxide) (PEO)-based electrolyte has been commercially available for small EVs since 2011 [15] and provides a combination of medium power, relatively high energy density (180 Wh kg^−1^ at pack level, 250 km driving range for the EV), long life (≥3000 cycles, no calendar aging), and safety. It is now equipped in electric vehicles and buses in several countries. However, it is recognized that wide market penetration for automotive application requires ranges > 500 km at an affordable cost with suitable safety, cycling life, and rate capability, as well as other key parameters, which are not yet simultaneously satisfied [16], but investigations to achieve such a performance are being pursued. Recently, Zhao et al. found that cationic aluminum species initiate ring-opening polymerization of molecular ethers to produce solid-state polymer electrolytes. Their application in Li//S, Li//LiFePO_4_, and Li//LiNi_0.6_Mn_0.2_Co_0.2_O_2_ batteries further demonstrated their high Coulombic efficiency (>99%) and long life (>700 cycles) [17].

Several reviews have focused on the solid-state battery technology from various perspectives. The advances in SSEs and GPEs for lithium batteries over the last decades can be found in different publications [18,19]. Recent reviews identify the major steps toward mass production of all-solid-state batteries, giving insights into the promising manufacturing technologies and battery designs [20,21]. Mechanical aspects, processing, and full cell integration challenges have been reviewed by Kerman et al. [22]. Attention has been focused on polymers in reference [23], and a historical overview has been presented in reference [24]. Other reviews focused on the advancements in enhancing the conductivity of solid electrolytes [25] and the energy chemistry between the solid electrolytes and the lithium metal anode [9]. Ionic conductivity depends on the contact between the electrolyte and the electrodes. Reviews on these interfacial behaviors can be found [26,27], and more specifically, on Li–S batteries in references [28,29,30]. In this field as well, fast progress has been achieved, owing to the emergence of new techniques such as spark plasma sintering (SPS) for fabricating ceramic solid-state electrolyte and electrode pellets with clean and intimate solid-solid interfaces [31]. Molecular layer deposition, which is an extension of atomic layer deposition (ALD), also finds important applications in the fields of batteries and supercapacitors; it is reviewed in reference [32]. The different mechanisms (like VTF, WLF, free volume theory, dispersed/intercalated mechanisms, etc.) have been discussed in order to explain the lithium ion conduction in polymer electrolyte systems and numerous characterization techniques and their results have been reviewed in reference [33]. We have recently reviewed the challenges and issues facing lithium metal for solid-state rechargeable batteries. [34]. The state of the art and the strategies to suppress dendrite growth by different techniques (surface modification of lithium metal, functional additives, and separators) have also been reviewed in several papers [35,36,37,38,39,40,41]. The cathode materials have been reviewed elsewhere [42]. Therefore, in the present review, attention is focused on the electrolytes, which are of crucial importance in solid-state batteries, and we provide a detailed overview on the updates, so that attention is directed at the results obtained recently, mainly since 2016, as the efforts to develop new solid electrolytes that are compatible with lithium have intensified. For prior works concerning the lithium–sulfur chemistry, we simply guide the reader to reviews that focus on the subject [43,44]. Other sections are devoted to the solid electrolytes in lithium–O_2_/air and lithium–sodium batteries, which are not at the same level of development, but have seen constant progress that justifies the intensive efforts devoted to them.

Transference number is an important parameter for obtaining a high cationic conductivity [45], and high transference numbers can be obtained only in single-ion conductors, such as ceramics, or the class of polymers in which the anions are fixed to the backbone and cannot move separately from the chains. An account on the advances in the development of single-ion-conducting electrolytes prior to 2016 can be found in reference [46]. A recent review on such electrolytes for lithium-metal batteries is reference [47]. However, the recent results are recalled for comparison with other electrolytes, emphasizing that polymeric single-lithium-ion conductors have not yet achieved a practical level of performance, particularly at room temperature.

Poly(ionic liquid)s (PILs) are also considered as promising as they are expected to retain the good properties of ionic liquids (such as high conductivity, thermal stability, and, in addition, an improved mechanical stability) owing to the covalent bonding of the ionic species with the polymer backbone [48].

Ceramic solid-state electrolytes exhibit some advantages compared to liquid electrolytes in that they have been known for decades, no concentration gradients are observed owing to single-ion conduction, and no dissolution process occurs [49]. Indeed, LiCoO_2_ exhibits a lower interfacial resistance in contact with an oxide solid electrolyte than with liquids [50]. The main efforts in the last few years have focused on how to eliminate the grain boundaries in solid-state electrolytes, as they are highly resistive and facilitate the formation of dendrites. In parallel, significant advances have been made in obtaining polymers with high ionic conductivities and increasing the limit of oxidative stability to ensure compatibility with the cathode belonging to the four-volt family. The composites including solid-state electrolytes and polymers, in principle, allow for a good compromise between high conductivity and good mechanical properties (soft enough to accommodate the change in volume during cycling and maintain a good contact between the electrodes and the electrolytes, but with Young’s modulus high enough to avoid the formation of dendrites on the anode side). These different types of electrolytes employed in all-solid-state batteries are reviewed hereunder. There is still a considerable gap between these laboratory-based achievements and commercialization [51], but the recent progresses at the laboratory level are promising and this gap can potentially be bridged in the years to come.

## 2. Solid Electrolytes for Lithium Batteries

Owing to the lack of polymers with electrochemical windows extending above 4 V until recently, the use of solid electrolytes restricted the choice of the cathode to LiFePO_4_. As we shall see, nevertheless, advances in the preparation of polymers compatible with lithium and stable up to 5 V now make it possible to use some other electrodes. We have separately presented the electrolytes used with LiFePO_4_ at room temperature and those used at higher temperatures in different sections, because the comparison between the electrochemical properties of cells having the same electrodes and used at the same temperature is more directly related to the performance of the electrolyte. For the same reason, the electrolytes used with the positive electrodes of the four- and five-volt families are reported separately from the LiFePO_4_-based case.

Solid-state batteries open the route to flexible batteries. In the case of lithium-ion batteries, flexible batteries have been the subject of intensive research for application in wearable electronics and have already been reviewed separately [52,53,54,55,56]. The Li–S battery with liquid electrolytes is at the doorstep of commercialization, whereas all-solid-state Li–S batteries are still in the research stage. It is thus not surprising that the investigation of flexible Li–S batteries is not as popular as that of flexible lithium batteries. Nevertheless, there has been growing interest, and an excellent review of flexible Li–S batteries can be found in reference [57]. Again, we guide the reader to these reviews, and even if we report on flexible lithium–oxygen or lithium air batteries hereunder, we do not devote a separate section to flexible batteries in this review. Therefore, the present review is focused on solid electrolytes for solid state batteries, including lithium batteries, Li–S, Li-air, or Li–O_2_ batteries, and sodium batteries, which are the most advanced solid-state batteries for practical applications.

### 2.1. Solid-State Electrolytes

Inorganic solid-state electrolytes are desired owing to their safety, thermal stability, high ionic conductivity, and long cycle life. However, they suffer from two problems: Insufficient solid–solid contact at the electrolyte/electrode interface, which produces a high interfacial resistance, and interfacial compatibility with either lithium metal or the cathode [58]. Intensive research has been carried out to identify appropriate materials and their optimized preparation conditions [59,60].

#### 2.1.1. Sulfur-Based Solid-State Electrolytes

Sulfur-based solid electrolytes have been investigated. They have been reported in reference [61], where the mechanism of their ionic conductivity has also been discussed. Among the various solid electrolytes such as sulfide, polymer, and oxide, sulfide solid electrolytes are considered as the most promising candidate for commercialization, although sulfide all-solid-state batteries cannot compete yet with liquid lithium-ion batteries [62]. Compared with the oxide-based solid electrolytes, they exhibit the advantage of a much higher ionic conductivity [63,64]. However, their electrochemical stability at anode potentials is usually too low for practical use. Such is the case, for example, of Li_7_P_3_S_11_ [65]. This glass-ceramic displays a high ionic conductivity (9.7 × 10^−4^ S cm^−1^ cm^−1^ at room temperature), and a high kinetic potential window of 5 V vs. Li^+^/Li [66], but it must not be in direct contact with lithium. It cannot be in contact with active cathode particles that contain cobalt either, such as LiCoO_2_, because it forms a highly resistive cobalt sulfide interface [67]. To avoid this problem, a LiNbO_3_@LiCoO_2_/Li_7_P_3_S_11_/Li cell was assembled, in which an LiF layer was added at the interface between Li_7_P_3_S_11_ and lithium by employing HFE-coated/infiltrated Li_7_P_3_S_11_ glass-ceramic as the electrolyte [68]. Owing to this protection of the lithium anode by LiF coating, this cell delivered a capacity of 118.9 mAh g^−1^ at 0.1 mA cm^−2^, which was retained up to 96.8 mAh g^−1^ after 100 cycles. At the least, it proves that interface engineering is a promising approach to overcoming the compatibility problem of sulfur-based solid electrolytes, but it renders the industrial process more complex and expensive. Note also that the cathode must be protected from sulfur-based electrolytes. That is true for Li_7_P_3_S_11_, and for many sulfur-based solid electrolytes, with the choice of LiCoO_2_, because of the formation of cobalt sulfide. This problem is solved by coating LiCoO_2_ with LiNbO_3_ [64,69], LiNb_0.5_Ta_0.5_O_3_ [70], or Al_2_O_3_ [71]. On the other hand, stable passivation interfaces are expected with other cathodes such as LiFePO_4_ and LiMn_2_O_4_ [67]. Li_2_S-P_2_S_5_ suffers from the same problem [72], even though adaptation of the process steps of conventional LIB production would be possible [21]. Again, the solution is coating of LiCoO_2_ particles. The Li_2_CO_3_-coated LiCoO_2_/Li_2_S-P_2_S_5_/Li cell exhibited enhanced rate capability, with a discharge capacity of 86.4 mAh g^−1^ at 0.5C, but the cycling life has not been explored [73]. The best architecture is the scheme devised by Ito et al.: A preliminary coating of nanometric LiCoO_2_ particles with LiNbO_3_, followed by coating with Li_2_S−P_2_S_5_, with Li_2_S−P_2_S_5_ being the electrolyte [74].

**Figure 1:**
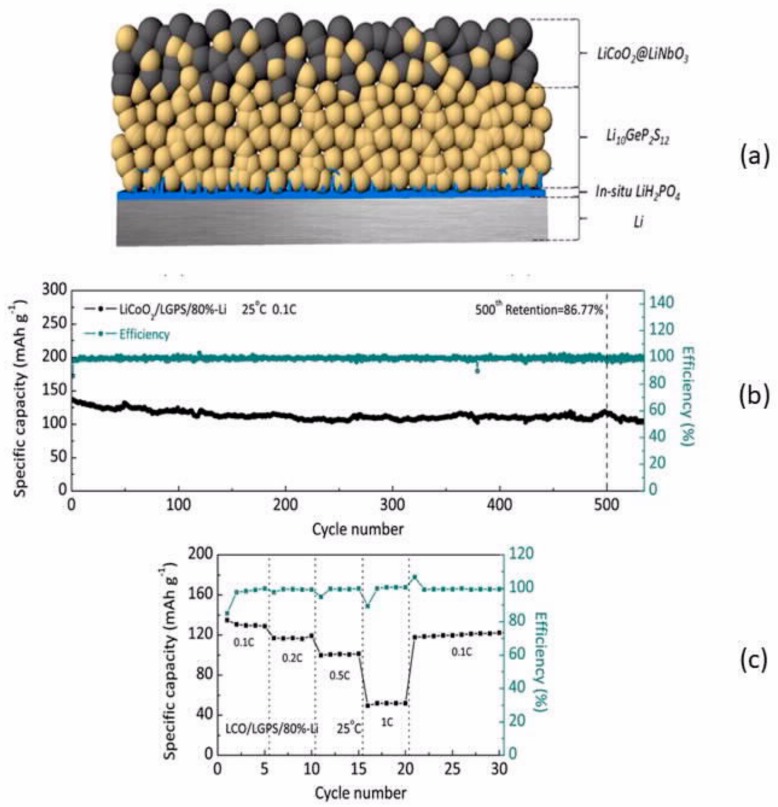
(**a**) LiCoO_2_ (LCO)/LGPS/LiH_2_PO_4_-Li all-solid-state battery. The optimized structure was obtained by in situ spin-coating of the lithium layer with 80 wt.% LiH_2_PO_4_. (**b**) Long cycle at 0.1C and (**c**) rate performance of the cell. Reproduced with permission from [84]. Copyright 2018 The American Chemical Society.

Adding P_2_O_5_ to Li_2_S−P_2_S_5_ slightly improves the cycling life but does not totally address the problem [75]. However, some progress has been achieved by using bilayers, even on the anode side. For instance, although Li_10_GeP_2_S_12_ (LGPS) is not compatible with Li^0^ [76], the bilayer LGPS/Li_3_PS_4_ exhibited improved electrochemical properties with different cathodes [77,78], which was the motivation for testing other bilayers as electrolytes [69,79,80,81]. On the other hand, LGPS, contrary to many sulfides, is compatible with cathodes containing cobalt, like LiCoO_2_. Many layered materials have been proposed, from which one can be selected to avoid a layer that leads to a high interfacial impedance and/or to not create interphases that proliferate through the LGPS electrolyte, which shortens the cycling life [82]. A 20 nm silicon thin layer fully satisfied the purpose, as LiCoO_2_//Li with this layer between lithium and LGPS electrolyte delivered a high capacity over 500 cycles [83]. However, the silicon layer was formed by pulsed laser deposition, which is an expensive process. A less expensive solution was to form in situ a LiH_2_PO_4_ layer [84], which yielded the same success (Figure 1).

**Figure 2:**
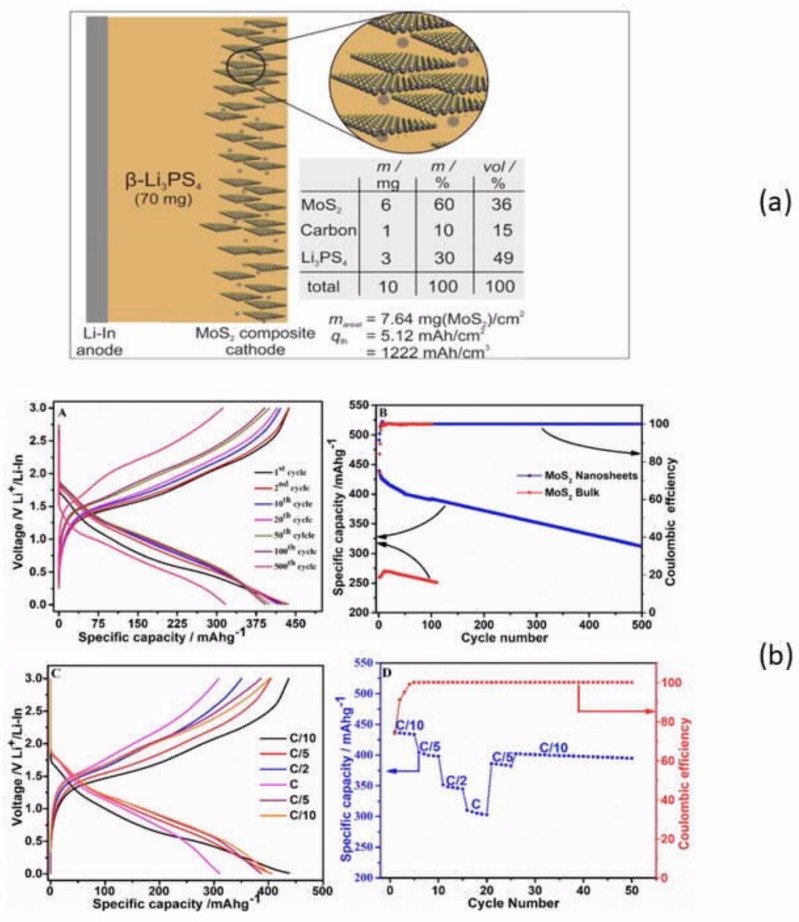
(**a**) Representation of the solid-state lithium-ion battery with MoS_2_ nanosheets as the positive and a lithium–indium alloy as the negative electrode, and β-Li_3_PS_4_ as the solid electrolyte. The table shows the specifications of the MoS_2_ composite electrode (absolute material amounts for the electrode with a diameter of 10 mm, weight, and volume fractions by assuming bulk densities), areal loading of MoS_2_, and theoretical capacity values based on *q*_th_ = 670 mAh g^−1^ (MoS_2_). The experimentally determined thickness of the MoS_2_ composite electrode is approximately 40 μm. (**b**) (A, B) Charge–discharge profile and cycling performance of MoS_2_ nanosheets at the current rate of 0.1C in the potential window 0.01–3.00 V vs. Li^+^/Li–In, and the corresponding Coulombic efficiency. (C, D) Rate performance charge–discharge profile and cycling performance of MoS_2_ nanosheets at different current densities in the potential window 0.01–3.0 V (Li^+^/Li–In). Reproduced with permission from [93]. Copyright 2019 The American Chemical Society.

A further improvement was to use a nanocomposite interphase formed by in situ electrochemical reduction of a liquid electrolyte on lithium anode [85]. This interface was composed of organic elastomeric lithium salts (LiO-(CH_2_O)_n_-Li) and inorganic nanoparticle salts (LiF, -NSO_2_-Li, Li_2_O). This interface enabled electrodeposition over 3000 h of repeated lithium plating/stripping. A solid-state Li/LGPS/TiS_2_ cell with this interlayer delivered an areal capacity of 0.18 mAh cm^−2^ at a current density of 0.1 mA cm^−2^, with a capacity retention of 91.7% after 200 cycles. However, germanium is rare and expensive and should be avoided for practical applications. One way to avoid LGPS is to simply use Li_3_PS_4_, which was employed as a solid-state electrolyte protection layer on the surface of lithium [86]. The Li_3_PS_4_ interlayer was formed by an in situ and self-limiting reaction between P_4_S_10_ and Li in N-methyl-2-pyrrolidone. As a result, symmetric Li-LPS cells (lithium electrode with a protective Li_3_PS_4_ layer) could deliver stable lithium plating/stripping voltage profiles for 2000 h, with a voltage hysteresis that was as low as 10 mV. Another approach to avoiding germanium is to replace LGPS by the less expensive tin; thus, Ju et al. fabricated the composite solid-state electrolyte polyvinylidene fluoride (PVDF)-Li_10_SnP_2_S_12_ via in situ polymerization, which is compatible with lithium metal [87]. Recently, Xie et al. simultaneously introduced Sb^5+^ and O^2−^ into the structure of β-Li_3_PS_4_ to synthesize Li_3_P_0.98_Sb_0.02_S_3.95_O_0.05_ [88]. This doping resulted in two beneficial effects. While a partially reversible conversion of PS_4_^3−^ to P_2_S_6_^4−^ was observed along with the formation of Li_2_S during cycling with pristine β-Li_3_PS_4_ [89], the doping significantly improved the compatibility with Li^0^ without affecting the stability, which now exceeded 5 V. Moreover, it ensured a good ionic conductivity of 1.08 × 10^−3^ S cm^−1^ at room temperature. β-Li_3_PS_4_ films are usually thick, because of the sensitivity to air. However, Wang et al. showed that ultrathin films of β-Li_3_PS_4_ with thicknesses controllable between 8 and 50 μm can be fabricated by evaporation-induced self-assembly technique without affecting the ionic conductivity or the electrochemical stability up to 5 V [90]. The all-solid-state lithium battery assembled with LiNbO_3_-coated LiCoO_2_ cathode, LGPS/Li_3_P_0.98_Sb_0.02_S_3.95_O_0.05_ electrolyte, and lithium metal anode (LGPS on the cathode side to avoid its contact with lithium) delivered a capacity of 133 mAh g^−1^ at 0.1C in the range 3.0–4.3 V vs. Li^+^/Li at room temperature, with a capacity retention of 78.6% after 50 cycles. Note that the LiNbO_3_-coating on LiCoO_2_ (and any cobalt-containing cathode) is commonly used to prevent cobalt–phosphorus exchange at the LiCoO_2_/β-Li_3_PS_4_ interface [91]. Li_3_PS_4_ can be also used as nanoparticles inside PEO matrix to improve the ionic conductivity of PEO. The optimal hybrid polymer PEO-2 vol.% Li_3_PS_4_ showed a conductivity of 8 ×10^−4^ S cm^−1^ at 60 °C. When it was used as an electrolyte in the solid-state LiFePO_4_/Li battery at this temperature, capacities of 153 and 127 mAh g^−1^ were obtained at 0.1C and 1C, respectively, with 80.9% retention rate after 325 cycles [92]. β-Li_3_PS_4_ was used recently as a solid electrolyte with MoS_2_ cathode, while the counter electrode was a lithium–indium alloy [93]. The cell delivered a capacity of 439 mAh g^−1^ at 0.1C, which remained at 312 mAh g^−1^ after 500 cycles, which proved that Li_3_PS_4_ is compatible with lithium–indium alloys (Figure 2).

Li_6_PS_5_*X* (*X* = Cl, Br, I) argyrodites exhibit a very high ionic conductivity of 2.58 × 10^−3^ S cm^−1^ for *X* = Br after annealing at the optimum temperature of 550 °C [94], and 1.8 10^−3^ S cm^−1^ before the annealing, with a relatively good stability toward metallic lithium [95]. The MoS_2_/Li_6_PS_5_*X* all-solid-state batteries assembled with Li_6_PS_5_Cl-coated MoS_2_ as the cathode, Li_6_PS_5_Cl as the solid electrolyte, and an indium-lithium alloy as the anode delivered a stable capacity of 350 mAh g^−1^ at the current density of 0.13 mA cm^−2^. At 1 mA cm^−2^, a linear decrease in the capacity was observed between 250 mAh g^−1^ at the 120th cycle and 210 mAh g^−1^ at the 250th cycle. However, in contact with lithium metal, Li_6_PS_5_*X* decomposes into an interphase composed of Li_3_P, Li_2_S, and Li*X*, which serves as a solid electrolyte interface (SEI) and increases the interfacial resistance [96]. The composite Li_6_PS_5_Cl-PEO with 5 wt.% PEO was used to reinforce the electrolyte and increase the ionic conductivity of PEO. LiNi_0.8_Co_0.1_Mn_0.1_O_2_/Li_6_PS_5_Cl-5%PEO/Li showed 91% capacity after 200 cycles at 0.05C and 30 °C, as the PEO added in Li_6_PS_5_Cl improved the stability against lithium and rendered this electrolyte compatible with the metal [97]. However, the initial discharge capacity was only 75.6 mAh g^−1^, because the ionic conductivity was too low. At 60 °C, the discharge capacity increased to 110 mAh g^−1^ at 0.05C. At this temperature and 0.5C, the capacity was 60 mAh g^−1^ with 44% retention after 500 cycles. Different from other oxygen-incorporated sulfides, the oxygen atoms in Li_6_PS_5−x_O_x_Br prefer to substitute the sulfur atoms at free S^2−^ sites, rather than those at the PS_4_ tetrahedra, and, for *x* = 0.3 (LPSOB-0.3), the ionic conductivity reaches 1.54 mS cm^−1^ at room temperature [98]. The compatibility with lithium was demonstrated, and all-solid-state batteries based on LiNi_0.8_Co_0.1_Mn_0.1_O_2_ (NCM811) positive electrode and lithium–indium negative electrode were devised by Zhang et al. using LPSOB-0.3. With NCM811, the cell delivered capacities of 108.7 mAh g^−1^ at 0.1C to 47.4 mAh g^−1^ at 0.8C, whereas the capacity at 0.8C vanished for *x* = 0. The authors of this recent work concluded that oxygen incorporation in sulfide SEs would be a universal strategy to improving the electrochemical performances of all-solid-state batteries.

In practice, carbon is added to the active particles of cathodes to improve the electrical conductivity. However, with solid-state electrolytes, contrary to the case of liquid electrolytes, this strategy does not necessarily work, because carbon can prevent the contact between the solid electrolyte and the powder of an electrochemically active material. That is why the wet-slurry process must be employed to fabricate sheet-type electrodes [61]. For instance, Jung et al. demonstrated that the capacity of an all-solid lithium cell with LiFePO_4_ importantly increased upon the addition of solvate ionic liquid LiG3, which is an equimolar complex of lithium bis-(trifluoromethanesulfonyl)imide (LiTFSI) and triethylene glycol dimethyl ether (G3) [99]. LiG3 is not miscible with nonpolar solvents because of immense differences in polarity. On the other hand, G3 is a strong Lewis base that reacts with electrophilic species such as P^5+^ in sulfide-based solid electrolytes, therefore it is not compatible with them. Solvents with intermediate polarity must then be used, among which is dibromomethane (DBM), which was chosen by Oh et al. for a slurry accommodating Li_6_PS_5_Cl (LPSCl) and solvate-ionic-liquid-based polymeric binders (NBR-Li(G3)TFSI, NBR:Nitrile−butadiene rubber) [100]. The ionic conductivity of LPSCl–NBR–LiG3 is 3.3 × 10^−3^ S cm^−1^. The electrodes prepared without using NBR suffered from peeling-off, which emphasizes the fact that a polymeric binder is mandatory. Unfortunately, the availability of polymeric binders with sulfur-based solid electrolytes is very limited, owing to their high reactivity. In practice, the choice is limited to NBR, styrene−butadiene rubber, and silicone rubber [101], and, for the same reason, the choice of solvent for wet-processing, with a few exceptions, is limited to toluene and xylene [80,99,102]. This limited choice makes it highly complicated to fabricate all-solid lithium batteries with sulfur-based solid electrolytes. Owing to the NBR binder, the LiNi_0.6_Co_0.2_Mn_0.2_O_2_ (NMC622)/LPSCl–NBR–LiG3/Li-In cell delivered a capacity of 174 mAh g^−1^ at 30 °C at 0.1C, for a concentration of NBR of 3 wt.%. The cell with LiNi_0.7_Co_0.15_Mn_0.15_O_2_ with a mass loading of 45 mg cm^−2^ revealed an areal capacity of 7.4 mAh cm^−2^. Besides DBM, which was already mentioned as an exception for the choice of solvent for wet-processing, tetrahydrofuran (THF) was used by Oh et al. In particular, these researchers prepared a slurry by adding NMC622, Li_3_PS_4_ precursors (Li_2_S and P_2_S_5_), polymeric binder (NBR), and carbon additive to THF [103]. This slurry was then cast and uniformly coated on aluminum current collector by doctor blade method. Then, heat treatment allowed THF to evaporate, thus resulting in a scalable single-step wet-chemical fabrication process for a sheet-type electrode. This is in contrast with the more typical fabrication based on dry-mixing of the active material powder plus solid-electrolyte plus conductive powders, which is difficult to scale up for batteries employing sheet-type electrodes because of their mechanically unstable features [102]. The same single-step wet-chemical process was employed to fabricate a graphite anode, which allowed for the fabrication of LiNi_0.6_Co_0.2_Mn_0.2_O_2_ (NCM622)/solid electrolyte/graphite that delivered a capacity of 131 mAh g_NCM622_^−1^ in the voltage range 2.50–4.15 V at 0.1C and 30 °C, which corresponded to an energy density of 241 Wh kg_NCM622+graphite_^−1^. At 100 °C, the capacity was 110 mAh g_NCM622_^−1^ at 15C, with the retention being 86% after 250 cycles. The results obtained with a lithium–indium anode are quite comparable to those obtained with LPSCl–NBR–LiG3 electrolyte in reference [100], with a slow decrease in the capacity as a function of the number of cycles. The capacity retention in the case of graphite anode, however, was very good because the interdiffusion of the sulfur-based electrolyte and lithium that induces a large interfacial resistance was avoided. This is also the reason that the rate capability is so high with the graphite anode. Finally, the good performance obtained at 100 °C cannot be achieved with liquid electrolytes because of the lower boiling temperature of the carbonates.

As part of a different approach, Kim et al. infiltrated liquefied LPSCl into LiCO_2_ cathode and then solidified it [80]. The LiCO_2_ particles of the electrode were coated with Al_2_O_3_ to avoid the formation of cobalt sulfide, as mentioned above. The full cell with LPSCl-infiltrated LCO and graphite electrodes with a conventional thick (≈ 600 μm) solid electrolyte layer cycled in the voltage range 2.0–4.3 V at 30 °C and delivered a capacity of 117 mAh g_LCO_^−1^ at 0.1C (0.14 mA cm^−2^), which corresponded to an energy density of 213 Wh kg_electrodes_^−1^. At 0.5C, the capacity was 75 mAh g_LCO_^−1^, which was very stable over 80 cycles of testing. This remarkable cyclability is attributed to the choice of graphite anode, which avoids contact between the sulfur-based electrolyte and lithium. The result also demonstrates that the strategy of using a homogeneous solid electrolyte solution that enables direct coating of highly conductive solidified electrolytes onto active materials for all-solid-state batteries is very promising. The interest in this strategy can also be inferred from the fact that the same group used 0.4LiI-0.6Li_4_SnS_4_ and Na_3_SbS_4_ electrodes in lithium and sodium batteries, respectively [69,104]. Li_4_SnS_4_ belongs to the group of tin-based electrolytes. They exhibit the advantage of being stable in air, which is contrary to the case of phosphorus-based solid electrolytes for reasons analyzed in reference [105]. Choi et al. fabricated a half-cell with Li_4_SnS_4_-coated LiCoO_2_ by adding LiCoO_2_ powder to a predissolved Li_4_SnS_4_ solution and Li_4_SnS_4_ solid electrolyte [106]. The capacity at 1C was 71% (97 mAh g^−1^) of the capacity at 0.1C. However, the cell failed after 120 cycles. We presume this might be due to the fact that, although the stability of SnS_4_^−4^ avoids the aggressive evolution of H_2_S in humid environments, it does not necessarily avoid the formation of the highly resistive cobalt sulfide in contact with a cathode containing cobalt, such as LiCoO_2_.

The strong interest in sulfide solid electrolytes dates back to only 2015, when a bendable sulfide solid electrolyte was reinforced by a mechanically compliant poly(paraphenylene terephthalamide) (PPTA) nonwoven (NW) scaffold [102]. The cell was prepared via the doctor-blade method to coat the sulfide solid electrolyte (SE) slurry on a nickel foil, followed by cold pressing onto the NW scaffold. The SE was a LGPS-Li_3_PS_4_ bilayer. The full all-solid-state battery consisting of LCO as the cathode and LTO as the anode showed an energy density of 44 Wh kg_cell_^−1^, which was still lower than that of commercial flexible lithium-ion batteries (100–200 Wh kg_cell_^−1^). The sulfide solid electrolytes are thermodynamically unstable with both lithium metal and high-voltage cathode materials, and extremely hygroscopic, producing toxic H_2_S upon contact with moisture. Nevertheless, it has been shown that the interface can be kinetically stabilized by forming artificial surface coatings or naturally fabricated SEI layers. Because of the fact that the sulfide electrolyte can be manufactured by a roll-to-roll process using machines similar to those used in LIB manufacturing, it has attracted intense the battery industry. In particular, agyrodite is stable with lithium metal without an artificial interfacial layer. Recently, some start-ups, battery manufacturers, and automotive companies (Toyota being one of them) announced aggressive production plans with sulfide-based electrolytes.

#### 2.1.2. Oxygen-Based Solid Electrolytes

**Figure 3:**
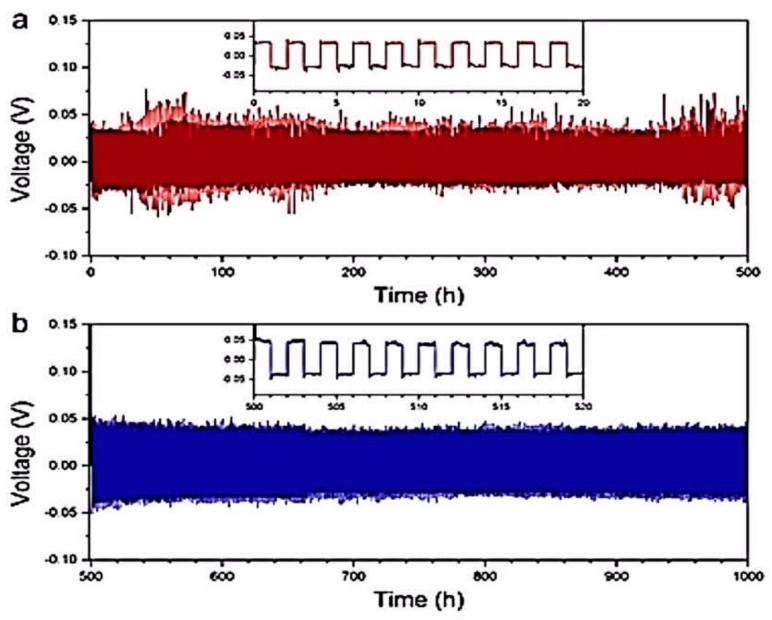
Electrochemical performance of lithium–magnesium alloy anode. (**a**,**b**) Voltage profile of lithium cycling at room temperature in a symmetric Li_0.93_Mg_0.07_/Li_6.75_La_3_Zr_1.75_Ta_0.25_O_12_/Li_0.93_Mg_0.07_ solid-state cell at 1 mA cm^−2^ for 1 h in each half cycle for a total of 500 h (**a**), followed by 2 mA cm^−2^ for 1 h in each half cycle for another 500 h (**b**). The average overpotentials during lithium plating/stripping at 1 and 2 mA cm^−2^ were 0.030 and 0.035 V, respectively. Reproduced with permission from [124]. Copyright 2019 Wiley.

Parallel to sulfide-based solid electrolytes, promising results have also been obtained with oxide-based solid electrolytes, even though their ionic conductivities are lower [107]. In particular, lithium-garnet solid electrolyte composite ceramics have emerged as a new class of inorganic fillers for SPEs owing to their high ionic conductivity, chemical stability vs. lithium metal, and wide electrochemical window [108]. The first problem with garnets, however, is the difficulty of synthesizing them without forming grain boundaries, which limit their mechanical strength and increase the resistance [109]. The second problem is the difficulty of maintaining contact at the interface with the electrode, which usually requires a buffer layer. However, the recent progress in solving these problems justifies the interest in these materials, and a review on the challenges and perspectives of garnet solid electrolytes for all solid-state lithium batteries has been published by Liu et al. [110]. A review on the stability against ambient air, lithium metal, and cathodes can be found in reference [111]. Among garnets, Li_7_La_3_Zr_2_O_12_ (LLZO) has attracted tremendous interest owing to a high ionic conductivity of up to 10^−3^ S cm^−1^ after doping [112,113,114,115], which is close to its maximum theoretical value [116], wide potential window (up to 9 V), and good chemical stability. However, it must be protected against humidity, and its stability in air is sensitive to the synthesis parameters [117,118]. In addition, the interfacial resistance between lithium and LLZO is low, provided that the lithium surface is free of lithium carbonate or LiOH impurity [119,120]. With LLZO, like in the case of any ceramic, some buffering layer is desirable to maintain contact between the lithium surface and the rigid ceramic. Yang et al. proposed a calcium-, niobium-doped LLZO electrolyte that consists of a dense layer as the separator and two porous layers for hosting lithium on one side and the active cathode material on the other side [121]. The upper layer of the garnet host was coated with ZnO by ALD to increase its wettability, with molten lithium infiltrating the pores of LLZO, following a process demonstrated by Wang et al. [122]. The process is expensive [123], but the solid lithium metal anode in the garnet host exhibited a very good cycling stability. It could be cycled for 1 mAh cm^−2^ at 0.5 mA cm^−2^ for 300 h without dendrite-induced short circuit or a significant overpotential. Yang et al. have shown that a lithium-ion and electron dual-conductive framework can be built by dealloying a lithium–magnesium alloy anode (approximately Li_0.93_Mg_0.07_) on LLZO electrolyte [124]. When lithium is stripped from the alloy anode, the lithium magnesium alloy becomes a lithium-deficient material with a porous framework, but still maintains good interface contact with LLZO, and the remaining lithium in the lithium–magnesium skeleton provides continuous pathways for both lithium ions and electrons. The lithium anode within the lithium magnesium host that melted on the garnet solid state electrolyte exhibited excellent cycling stability for 500 h at 1 mA cm^−2^ and for a further 500 h at 2 mA cm^−2^, totaling 750 mAh cm^−2^ cumulative plating capacity (Figure 3).

Good results were also obtained by Duan et al. with an asymmetric solid electrolyte (ASE) [125]. This 36 μm thick ASE was composed as follows. On the lithium metal side, a LLZO layer modified with a 7.5 nm polymer electrolyte on the surface established a rigid barrier with a high elastic modulus to prevent dendrite penetration. On the cathode side, a soft layer of a polymer electrolyte with thickness below 5.4 μm was in good contact with the active particles to reduce the interfacial resistance. The polymer was poly(ethylene glycol-methyl ether-acrylate). The corresponding Li/ASE/LiFePO_4_ cell delivered a capacity of 160 mAh g^−1^ at 0.2C and 55 °C, with the capacity retention being 94.5% after 120 cycles. Hu et al. proposed the use of a germanium layer to modify the Li/LLZO interface in the Li/LLZO/LiFePO_4_ system; the modified interface exhibited a stable cycling performance at room temperature [126]. Such a chemistry, however, is not scalable, because germanium is rare and too expensive to be used in industry.

Yan et al. fabricated an ultrathin Li/LLZO/LiFePO_4_ all-solid-state battery without cold or hot-pressing. The LLZO particles were prepared via a solid-state reaction, and LLZO slurry was prepared using ball-milling. An ultrathin electrolyte film was obtained by wet coating the final slurry on the prepared cathode. The cell showed a discharge capacity of 160.4 mAh g^−1^, which was maintained at 136.8 mAh g^−1^ after 100 cycles at room temperature [127]. Note that oxide solid electrolytes display large electrochemical windows. Therefore, a good strategy to increasing the energy density of solid-state lithium batteries is to couple the lithium anode and high-voltage cathode with solid-state electrolytes [128,129,130,131,132]. However, their direct contact with 4 V cathodes such as LiCoO_2_ must be avoided. With LLZO, Kim et al. found an irreversible electrochemical decomposition at ∼3.0 V vs. Li^+^/Li, which could be avoided by the surface modification of LLZO (e.g., deposition of a cobalt-diffused surface layer and/or the presence of an interlayer such as Li_3_BO_3_) [133]. An interface-engineered all-solid-state lithium battery has also been proposed for LiCoO_2_/LLZO, where LiCoO_2_ is used as the cathode [134], and for the Li_4_Ti_5_O_12_/LLZO interface with Li_4_Ti_5_O_12_ as the anode [135]. In the same way, oxide solid electrolytes cannot be used in direct contact with high-voltage spinel cathodes because of the formation of dense cathode composites between the spinel cathodes and oxide electrolytes, which results in high-impedance interfacial products due to the fact that the oxygen lost from the cathode is absorbed by the ceramic electrolyte [136].

A different approach proposed by He et al. involved the introduction of succinonitrile (SCN) and a salt (LiTFSI in this work) to improve the contact between the garnet solid electrolyte (niobium-doped LLZO) and the cathode particles [137]. SCN was chosen because of its plastic crystal nature and its ability to dissolve many salts. A solid Li//LiFePO_4_ cell was prepared with the LiTFSI-SCN composition of 7.5 mol% LiTFSI that led to the highest ionic conductivity (1.27 × 10^−3^ S cm^−1^ at 20 °C). This cell delivered a capacity of 149.8 mAh g^−1^ after 100 cycles at 0.05C, which showed that the network is flexible enough to accommodate the volume change of LiFeO_4_ during cycling, at least at this very low rate. The capacity delivered at 1C was still 106.7 mAh g^−1^, which indicated that the conductivity is high enough to maintain a good capacity at a higher rate. It remains to be tested whether, at this higher rate, the cycle stability is maintained, but the fact that the initial capacity at 0.05C is recovered after the tests at 1C is promising.

Another solution is to disperse LLZO into a polymer matrix to avoid contact between the garnet and electrodes. In particular, LLZO (70% in weight) dispersed into a P(EO)_15_LiTFSI polymer electrolyte matrix (30 wt.%) via solvent-free processing was fabricated by Keller et al. The LLZO-P(EO)_15_LiTFSI hybrid electrolyte showed improved compatibility with lithium metal compared to that of pure P(EO)_15_LiTFSI and pure ceramic LLZO electrolytes, which are known to form poor interfacial contacts with lithium metal [138].

LLZO exhibits two kinds of phases: Tetragonal and cubic. Unfortunately, from a thermodynamic point of view, the tetragonal phase, which reveals a conductivity that is two orders of magnitude lower than that of the cubic one, is more stable at room temperature [139]. The substitution of Li^+^ by other ions such as Al^3+^ or Ga^3+^ is necessary for the stabilization of cubic LLZO at room temperature [140,141]. Moreover, the substitution of Zr^4+^ by Nb^5+^, Ta^5+^, Mo^6+^, etc., can further increase the ionic conductivity owing to a sufficiently high vacancy concentration that disrupts the ordering [142,143]. In particular, Li_7_La_2.75_Ca_0.25_Zr_1.75_Nb_0.25_O_12_ was selected by Fu et al. to introduce a thin layer of aluminum between the garnet solid electrolyte and the lithium metal interface. As a result, an ionically conducting lithium–aluminum alloy is formed, which acts as an interfacial layer between the garnet SSE and the lithium metal anode [123]. This design changes the wettability of the garnet surface (from lithiophobic to lithiophilic) and reduces the interface resistance by more than an order of magnitude: 75 Ω cm^2^ against 950 Ω cm^2^ for the pristine garnet/lithium interface. More stable cell performances were obtained with this design for lithium-ion, lithium–sulfur, and lithium–oxygen batteries, provided a liquid electrolyte was added between the garnet electrolyte and the cathode. Indeed, according to Aguesse, the addition of a liquid interfacial layer between the cathode and the ceramic electrolyte is a prerequisite for achieving a low interfacial resistance and for full utilization of the active material [144]. However, the battery is no longer an all-solid-state battery; the design of polymer/solid electrolyte/polymer electrolyte would maintain this label. An example will be given below when reporting the results obtained with a sodium superionic conductor (NASICON)-type material.

At the tantalum-doped LLZO (LLZTO)/Li interface, the SEI resistance is high, unless a surface treatment is performed to increase the ionic contact and wettability. This can be achieved by coating the garnet with 10 nm thick amorphous silicon that is deposited by plasma-enhanced chemical vapor deposition [145], conformal ZnO surface coating of the garnet by ALD [122], or deposition of a lithium electrode on a LLZTO pellet by vacuum-evaporation [146]. It should be noted that a homogeneous contact between LLZTO (or LLZO) and lithium is mandatory to avoid the formation of lithium dendrites [147]. The introduction of Li_3_PO_4_ as an additive to LLZTO also improved the interfacial compatibility and suppressed the growth of lithium dendrites owing to the formation of Li_3_P [148]. The drawback, however, is a decrease in the ionic conductivity to 1.4 × 10^−4^ S cm^−1^. No test on a half-cell has been carried out.

**Figure 4:**
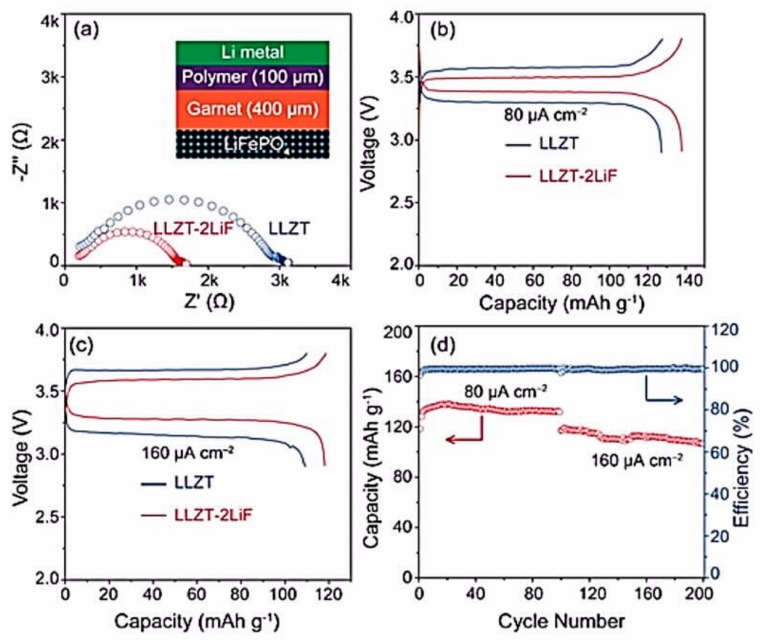
(**a**) Impedance plots of Li/LLZTO-2LiF/LiFePO_4_ battery, where LLZTO-2LiF stands for 2 wt.% LiF added to Li_6.5_La_3_Zr_1.5_Ta_0.5_O_12_. Charge and discharge voltage profiles of Li/LLZT/LiFePO_4_ at (**b**) 80 and (**c**) 160 μA cm^−2^. (**d**) Capacity retention and cycling efficiency of LiFePO_4_/Li cells at 80 and 160 μA cm^−2^. Reproduced with permission from [149]. Copyright 2017 Wiley.

Li et al. introduced 2 wt.% LiF to tantalum-doped Li_6.5_La_3_Zr_1.5_Ta_0.5_O_12_ (LLZTO-2LiF) garnet to increase the stability of the garnet against moisture, and added a lithium-ion conducting cross-linked polyethylene oxide (CPEO)-LiTFSI polymer that acts as a buffer layer between the lithium anode and the garnet (Figure 4) [149].

The all-solid-state Li/CPEO-LiTFSI/LLZTO-2LiF/LiFePO_4_ delivered capacities of 142 and 128 mAh g^−1^ at 80 and 160 μA cm^−2^, respectively. The capacity was retained at 120 mAh g^−1^ after 100 cycles at 80 μA cm^−2^. This work illustrates that the introduction of tantalum-doped LLZO powders, which we will refer to as LLZTO (the Ta concentration may differ from one work to another), into SPEs promotes complete dissociation of lithium salt as well as enhances the migration of Li^+^. As a result, the conductivity is increased up to 4 × 10^−4^ S cm^−1^ [150], which allows use of the cells at room temperature. In addition, LLZTO prohibits the formation of ion clusters in the electrolyte membranes [151]. Note that the choice of the salt is also important to achieve this result. LiTFSI is a sulfonamide-based salt and is thus capable of enhancing the ionic conductivity by effectively decreasing the crystallinity of SPEs and promoting the dissociation of Li^+^ due to the flexible S–N bond and the highly delocalized negative charge of sulfonimide anion [152]. Until now, LiTFSI is the only source of Li^+^ that can be used to obtain a polymer electrolyte that is endowed with an ionic conductivity that reaches 10^−4^ S cm^−1^; another example is LiTFSI in a polymer prepared by mixing PEO as the polymer matrix and bis[2-(2-methoxyethoxy)ethyl] ether (tetraglyme; tetraethylene glycol dimethyl ether (TEGDME)) as the active plasticizer [153]. The role of TEGDME has been clarified by investigation of the ionic transport in LLZO-PEO (LiClO_4_)-TEGDME composite by high-resolution solid-state nuclear magnetic resonance [154]. This study shows that the transport occurs mainly via TEGDME-associated phases. However, the beneficial effect is only short-term, as a decrease in active lithium sites and degradation of ionic conductivity with time are observed, which limit the interest in TEGDME.

A high concentration of LLZTO in the composite guarantees mechanical strength but results in poor contact with the electrodes. On the other hand, low concentrations result in the opposite and a lower conductivity, which is of the level of that of the polymer. In an attempt to find a compromise, Huo et al. recently fabricated a sandwich PIC/CIP/PIC electrolyte [155]. The “polymer in ceramic” (PIC) was composed of 80 vol.% LLZTO in the form of particles 5 μm in size in PEO, whereas the “ceramic in polymer” (CIP) was composed of 20 vol.% LLZTO particles 200 nm in size in PEO. At 30 °C, the LiFePO_4_//Li cell with this composite electrolyte delivered a capacity of 118.6 mAh g^−1^ at 0.1C, with Coulombic efficiency being 93.4%, and a capacity retention of 82.4% after 200 cycles.

Another approach used by He et al. was to insert a tin layer between the lithium anode and Li_0.33_La_0.557_TiO_3_ (LLTO) doped with niobium, instead of tantalum (LLZNO) [156]. Li/Sn-LLZNO/LiFePO_4_ delivered a capacity of 167 mAh g^−1^ at room temperature at 0.1C, with a capacity retention of 99.6% after 100 cycles.

LLZTO is not the only possible choice of ceramic electrolyte. Li_1.5_Al_0.5_Ge_1.5_(PO_4_)_3_ (LAGP) has also been considered owing to its high conductivity that can reach 1 × 10^−3^ S cm^−1^ [157,158], despite the fact that a compound with germanium has no future on the industrial scale. Hereon, we refer to Li_1+x_Al_y_Ge_2−y_(PO_4_)_3_ ceramics by the generic term LAGP, irrespective of the values of x and y, which may slightly differ from one work to another. Li et al. used a LAGP with a high-salt-concentrated polymeric electrolyte comprising poly(propylene carbonate) (PPC) and lithium bis(fluorosulfonyl)imide (LiFSI) that was prepared with an optimum salt concentration of 80 wt.% [159]. Owing to this high concentration, the ionic conductivity reached 10^−4^ S cm^−1^ at ambient temperature, the lithium transference number was 0.75, and the anodic stability 4.5 V vs. Li^+^/Li. The LiFePO_4_//Li cell with LAGP/PPC-LiFSI 80 wt.% composite electrolyte delivered a specific discharge capacity of 138.3 mAh g^−1^ at 0.1C and a high capacity retention of 97.1% after 100 cycles at room temperature. These results show that ceramic/high-salt-concentrated PPC-based polymer composite electrolytes are promising for ambient temperature solid-state lithium batteries.

In a different approach, the PEO-based SPE of 1% PEO-75% Li_2_S-24% P_2_S_5_-1% P_2_O_5_ was coated on a LAGP pellet as a composite electrolyte (CE; LAGP/SPE) for the construction of the all-solid-state LFP//Li cell [160]. The lithium-ion conductivity of the cathode layer had also been optimized via the incorporation of PEO-LiClO_4_ into LFP. The discharge capacity was maintained at 127.8 mAh g^−1^ after the 1000th cycle at 1C, with a retention of 96.6%, and the initial discharge capacity was 153.4 mAh g^−1^, with a high retention of 99.9% after 200 cycles at 0.1C.

LAGP has also been chosen to fabricate hybrid solid electrolytes (HSEs) composed of 3D ordered bicontinuous conducting ceramic and insulating polymer (epoxy) microchannels [161]. The ceramic channels provide continuous pathways, which help in maintaining a high ionic conductivity between the electrodes, while the polymer channels permit improvement of the mechanical properties compared to those of the ceramic alone, in particular, mitigation of the brittleness of ceramics. Printed templating permits not only the control of the ceramic-to-polymer ratio, but also the micro-architecture. The best electrical and mechanical properties were obtained with the gyroidal structure, in which case the conductivity was 1.6 × 10^−4^ S cm^−1^ at room temperature, with a compressive failure strain that was 28% higher than that of a LAGP pellet. Only the compatibility with lithium has been tested yet, but the result is promising, and tests of the electrochemical properties on half-cells are highly desired.

Another NASICON-type ceramic Li_1+x_Al*_x_*Ti*_2-x_*(PO_4_)_3_ (LATP) has attracted much attention because of its high lithium ion conductivity that can reach 3.15 × 10^−4^ S cm^−1^ at room temperature [162] and relatively low-cost synthesis [163]. It also offers the advantage of promoting the salt dissociation, owing to possible interactions between LATP (a Lewis acid) and the salt anion (e.g., TFSI^−^, a Lewis base), which may result in the formation of an “ion-ceramic complex” [158]. It should be noticed that LATP, contrary to LLZO, is reduced when in contact with lithium anode, which leads to the formation of electron-conducting phases, and thus, an increased short-circuiting risk [164,165]. This drawback of LATP, however, does not preclude its use, provided that it is associated to a polymer that not only acts as a buffer, but also avoids direct contact between LATP and lithium. For instance, Ban et al. chose PEO-LiClO_4_. Li/PEO-LiClO_4_ 50 wt.% LATP/LiFePO_4_ battery at 80 °C still delivered a capacity of 109 mAh g^−1^ after 500 cycles at 1C [166].

**Figure 5:**
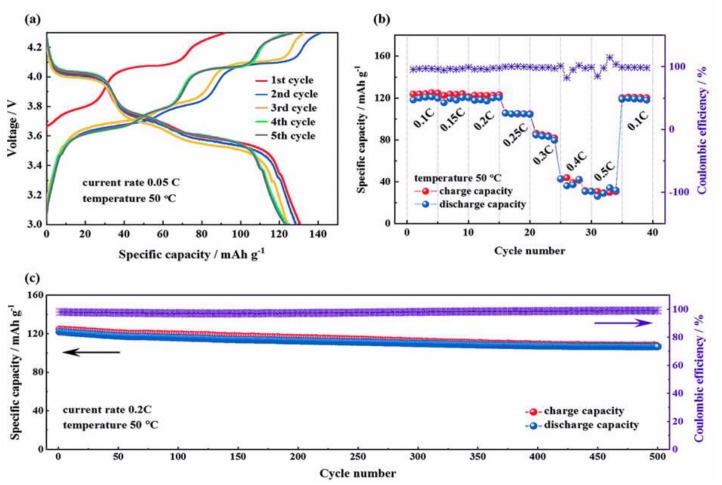
Electrochemical performance at 50 °C in the voltage range 3.0–4.3 V vs. Li+/Li of a cell with lithium anode, Li_3_V_2_(PO_4_)_3_/CNT cathode, and a solid electrolyte consisting of the ceramic LATP with a protective SPE consisting of polyphosphazene/PVDF–HFP/LiBOB: (**a**) Charge–discharge curve for the initial five cycles at a current rate of 0.05C, (**b**) rate capability at current rates from 0.1C to 0.5C, and (**c**) long-term cycling measurement at a current rate of 0.2C. Reproduced with permission from [168]. Copyright 2019 Royal Society of Chemistry.

Another polymer that is chosen in association with LATP is PVDF, which can promote the dissociation of lithium salts, owing to the polarity of the CF_2_ groups in its main chain. However, despite being polar, PVDF is not solvating; thus, traces of casting solvent may be involved. Yet, the LiMn_2_O_4_/LATP-PVDF/Li cell delivered 117 mAh g^−1^ at 0.2C, and the capacity was retained at 107 mAh g^−1^ after 200 cycles at room temperature [167]. The cell still delivered 92 mAh g^−1^ at 2C. This is an example of the benefit that can be obtained from the high-voltage stability of LATP for use in an electrolyte with a spinel cathode of the 4 V class to increase the energy density, while its high ionic conductivity improves the rate capability. A layered HSE SPE/LATP/SPE designed by coating the ceramic LATP electrolyte with a protective SPE consisting of polyphosphazene/poly(vinylidene fluoride-co-hexafluoropropylene) (PVDF–HFP)/lithium bis(oxalato)borate (LiBOB) was evaluated with a metallic lithium anode and the high-voltage Li_3_V_2_(PO_4_)_3_/CNT cathode (Figure 5) [168]. The cathode was chosen to take advantage of the stability of the electrolyte up to 4.7 V. This cell showed a high capacity and excellent cycling performance with negligible capacity loss over 500 cycles at 50 °C, but only at low C-rates, ≤0.2C, because the SPE conductivity at this temperature is only 2.6 × 10^−4^ S cm^−1^. LATP was also inserted in the form of vertically aligned channels in PEO polymer matrix, and the conductivity of this electrolyte (5 × 10^−5^ S cm^−1^) was 3.6 times that of the composite electrolyte with randomly dispersed LATP nanoparticles [169]. The improvement in the conductivity by vertical alignment of ceramics was confirmed by the results obtained with vertically aligned nanowires [170,171].

To date, except the chlorine-doped silicon analogue of LGPS, Li_9.54_Si_1.74_P_1.44_S_11.7_Cl_0.3_, which exhibits an ionic conductivity of 2.5 × 10^−2^ S cm^−1^ at room temperature [64], the highest bulk lithium ion-conducting solid electrolyte is the perovskite (ABO_3_)-type lithium lanthanum titanate Li_3*x*_La_(2/3) −*x*_□_(1/3)−*2x*_TiO_3_ (LLT, 0 < *x* < 0.16). For x of ~0.1, the conductivity reaches 1 × 10^−3^ S cm^−1^ at room temperature [172]. However, the use of LLT as an electrolyte is not favorable because LLT is not stable in direct contact with lithium and undergoes easy and fast lithium insertion, with the consequent reduction of Ti^4+^ to Ti^3+^, which leads to a high electronic conductivity. Moreover, its lithium uptake is very small, and the grain boundaries importantly reduce the ionic conductivity [173]. On the other hand, the family of low-cost lithium-rich anti-perovskite conductors Li_3_O*A* (*A* = halogen) has shown great promise for solid electrolytes owing to their ionic conductivities that exceed 10^−3^ S cm^−1^ at room temperature and very low electronic conductivities [174,175,176]; they are, of course, thermodynamically stable against lithium [177].

However, the ionic conductivity reported from subsequent investigations was much lower, ranging from 5 × 10^−7^ to 2 × 10^−4^ S cm^−1^ [178,179,180], with evidence that the “Li_3_O*A*” was actually Li_2_OH*A*. A closer look at the XRD spectra in reference [174] clearly reveal LiCl to be the main additional phase. Its presence indicates the formation of a chlorine-deficient compound, which presents evidence for the formation of OH-based lithium-rich anti-perovskites, rather than “OH-free Li_3_OCl” [181]. Substitution of F^−^ for OH^−^ transforms the anti-perovskite Li_2_OHCl into a cubic phase that shows electrochemical stability up to 9 V vs. Li^+^/Li and lithium-ion conductivity that is two orders of magnitude higher [182]. Consequently, the lithium/fluorine-doped Li_2_OHCl/LiFePO_4_ all-solid-state battery showed good cyclability and a high Coulombic efficiency over the 40 charge/discharge cycles tested. By partial substitution of halogen “A” with the super-halogen BH_4_, Fang et al. deduced from density functional theory (DFT) calculations that Li_3_OCl_0.5_(BH_4_)_0.5_ would also display a conductivity of 10^−3^ S cm^−1^, owing to the translation and rotation of the BH_4_^−^ super-halogen upon thermal excitation, which generate different orientational symmetries of the BH_4_ tetrahedra [183]. This mechanism explains the superionic conduction reported for lithium and sodium salts containing BH_4_^−^ [175,184]. It also justifies its use in the replacement of halogens to synthesize new perovskite crystals [185] with the aim of designing new inorganic–organic hybrid perovskites for solar cells [186,187] and to prepare new 2D hybrid perovskites for light-emitting diode (LED) applications [188]. Unfortunately, anti-perovskite electrolytes are very hygroscopic and should be operated in an inert atmosphere, which renders their practical application in solid-state batteries difficult [189].

It should be noted that the ceramic pellets utilized in the electrolytes used in the laboratory are usually ceramic powders that are pressed into thick pellets and sintered, with only a few exceptions such as what was reported in reference [127]. The sintering process, however, is not easy to use in an industrial process. In addition, this sintering usually involves high temperatures, although the cold sintering process offers great potential in the preparation of solid-state batteries and solid electrolytes in the future [190,191]. However, it is now possible to use flame spray pyrolysis to prepare 4 cm^2^ flexible films of aluminum-doped LLZO, which provides a pathway for large-scale industrial production of ceramic electrolytes [192].

Some attempts have also been made to build flexible batteries using oxide-based solid state electrolytes as ceramic fillers by taking advantage of the fact that the ionic conductivity of a polymer is enhanced when an oxide solid state electrolyte like LLTO is incorporated in the form of nanowires [193] or nanofibers [194], especially when the nanofibers are well-oriented [169], as nanofibers facilitate ionic conduction along them, without the obstacle of a resistive interface. In addition, they reinforce the skeleton for flexible electrolytes [195]. Zhai et al. fabricated a flexible solid composite electrolyte with LTP nanofibers that were vertically aligned and connected through an ice-templating process in PEO matrix [169]. The electrolyte maintained its integrity over 100 bending cycles.

### 2.2. Ionic Liquid-Based Systems and PILs

We also note that, even without any ceramic, high salt concentrations in ionic liquid systems is a good strategy for decoupling lithium- or sodium-ion transport from the bulk dynamics [196,197,198]. The reason is that ion speciation leads to complexes and aggregates that can percolate through the electrolyte, which presents another diffusion mechanism. This was studied in the case of lithium-coordination in TFSI, both experimentally [199,200] and by molecular dynamics simulations [201]. These electrolytes seem to avoid the formation of dendrites on lithium or sodium metal even at high C-rates [196,202]. In particular, the reduction in the formation of dendrites upon increasing the salt concentration has been evidenced by analysis of the SEI with lithium metal in the case of phosphonium bis(fluorosulfonyl)imide ionic liquid electrolyte in references [203,204].

PILs have been considered as promising as they are expected to retain the good properties of ionic liquids (high conductivity, thermal stability, and, in addition, improved mechanical stability) owing to the covalent bonding of the ionic species with the polymer backbone. However, a good compromise between ionic conductivity and mechanical strength is yet to be realized. A hierarchical PIL-based solid electrolyte was obtained by in situ polymerizing 1,4-bis[3-(2-acryloyloxyethyl)imidazolium-1-yl] butane TFSI (C1-4TFSI) monomer in an electrolyte composed of LiTFSI and 1-ethyl-3-methylimidazolium TFSI and then filling a poly(diallyldimethylammonium) TFSI (PDADMA-TFSI) porous membrane [48]. A conductivity of ≈ 10^−3^ S cm^−1^ was observed at room temperature, but the transference number was only *t*_Li+_ = 0.20, owing to the mobility of the TFSI^−^ ions. The LiFePO_4_//Li cell with this electrolyte delivered 155 mAh g^−1^, was stable over 60 cycles at 0.1C, but then, the capacity started to decrease to 147 mAh g^−1^ at the 100th cycle. The same electrolyte with LiTFSI replaced by NaTFSI was tested as an electrolyte in a Na_0.9_[Cu_0.22_Fe_0.30_Mn_0.48_]O_2_/Na cell, but then the capacity decreased almost linearly upon cycling from 100 to 85 mAh g^−1^ at 0.1C.

**Figure 6:**
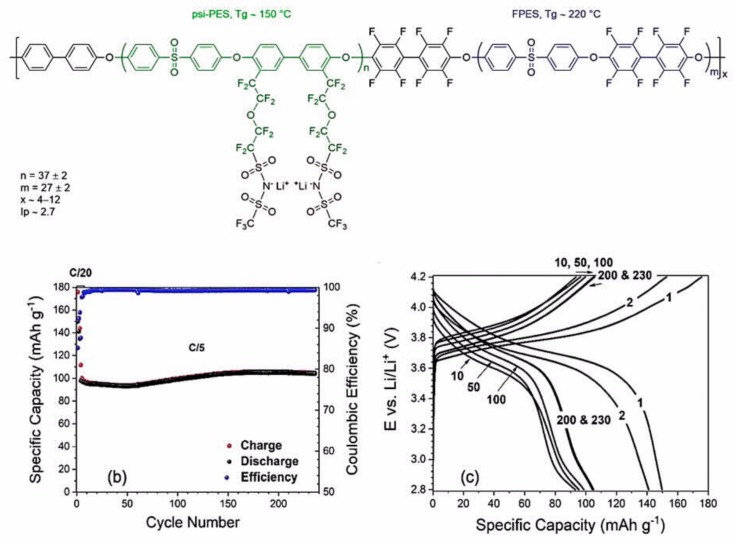
(**a**) Partially fluorinated multiblock copoly(arylene ether sulfone)s bearing lithium perfluorosulfonimide functioning as ionomer (SI) were synthesized in the work of reference [206]. To obtain the polymer electrolyte (SI-S55), the dried ionomer was infiltrated with ethylene carbonate by immersing the membrane in dimethyl sulfoxide (DMSO) solvent during the time required to obtain 55 wt.% DMSO. (**b**) Discharge/charge capacities and Coulombic efficiency as functions of cycle number of Li/SI-S55/LiNi_1/3_Mn_1/3_Co_1/3_O_2_ cells; the first three cycles were conducted at C/20, all the following ones at C/5 (1C = 160 mA g^−1^). (**c**) The corresponding potential profiles for the selected cycles. The cutoff potentials were set to 2.8 and 4.2 V vs. Li/Li^+^ and the cells were kept at a constant temperature of 40 °C. Reproduced with permission from [208]. Copyright 2018 Royal Society of Chemistry.

The ionic liquid can be solidified by combining it with a polymeric matrix that not only provides mechanical integrity but also may be involved in the conduction process to form a so-called “ion gel.” In particular, an ion gel electrolyte was fabricated in which the PIL poly(DADMA-TFSI) with a high molecular weight was used as a host polymer and combined with a superconcentrated IL-based electrolyte solution composed of LiFSI in tri-methyliso-butyl phosphonium bis(fluorosulfonyl)imide (P_111i4_FSI) ionic liquid [205]. An extremely stable polarization was observed in Li//Li symmetrical cycling tests. Replacing TFSI by FSI in the PIL, 50 wt.% PIL–50 wt.% electrolyte solution was used as the electrolyte in a Li//LiFePO_4_ cell at C/15 rate at 50 °C; it showed a stable capacity of approximately 120 mAh g^−1^ over 25 cycles, which corresponded to an initial areal capacity of 0.8 mAh cm^−2^ [206], which is a good result if we take into account that it is challenging to obtain large areal capacities with solid-state batteries. A ternary polymer electrolyte consisting of poly(styrene-b-1-((2-acryloyloxy)ethyl)-3-butylimidazolium bis(trifluoromethanesulfonyl)imide) (S-PIL_64−16_) PIL block copolymer with a high LiFSI salt concentration and a low content of N-propyl-N-methylpyrrolidinium bis(fluorosulfonyl)imide (C3mpyrFSI) ionic liquid was proposed by Goujon et al. [207]. Its ionic conductivity was only 6.6 × 10^−6^ S cm^−1^ at 50 °C, but the lithium transference number was high (0.53 at 50 °C). A Li//LiFePO_4_ cell with LiFePO_4_ loadings of 10 mg cm^−2^ and 1.8 mAh cm^−2^ using this electrolyte delivered an initial capacity of 167 mAh g^−1^ at C/20 at 50 °C; however, 3% capacity loss was observed after eight cycles. On the other hand, a nanostructured block copolymer consisting of polystyrene blocks and a perfluorinated sulfonimide anionic block, which was plasticized with ethylene carbonate, afforded much better results (Figure 6) [208].

## 3. Polymer Electrolytes

PEO-based materials are widely used as polymer hosts in commercial solid-state electrolytes. The main limitation of PEO originates from the high crystallinity of the ethylene oxide (EO) chains, which results in a low ionic conductivity. Nevertheless, progress has been made to increase this conductivity by different methods such as blending, modifying, and preparing PEO derivatives, which have been reviewed in reference [209]. The role of propylene carbonate (PC) as a plasticizer in PEO-LiClO_4_ has been investigated in reference [210]. The addition of PC in the polymer increases the interactions between different ion species such as ClO_4_^−^, Li^+^, the carbonyl oxygen in PEO, and the lone pair electrons of the C=O bond of PC. As a result of this new PEO-Li^+^-PC pathway, a maximum conductivity of 16 × 10^−3^ S cm^−1^ (which seems very high) at room temperature was obtained with 40 wt.% PC. At larger PC concentrations, the conductivity decreased again, due to an increase in ion-pair concentration. However, the highest conductivity with the PEO-LiClO_4_ system was obtained by adding 10 wt.% LATP nanoparticles, in which case the conductivity reached 1.7 × 10^−4^ S cm^−1^ at 20 °C owing to the cation transport within the interphase region surrounding the particles, and percolation was achieved at low nanoparticle loadings [211]. A remarkable result was obtained recently by enhancing the interfacial contact between the PEO-based electrolyte and the cathode. The strategy used by Chen et al. was to prepare a cathode-supported solid-state electrolyte membrane that was directly cast on the cathode layer [212]. The membrane was made of PEO, PVDF, and LiTFSI, with particles of Al_2_O_3_ as the plasticizer, and the cathode was LiFePO_4_. The corresponding cell with lithium metal counter electrode at 30 °C delivered capacities of 125 and 90 mAh g^−1^ at 0.1C and 0.2C, respectively. At 50 °C, the capacity was raised to 167 and 137 mAh g^−1^ at 0.1C and 0.5C, respectively. Similar results have been obtained with TiO_2_ particles as plasticizer [213]. However, the conductivity of PEO is still too low at room temperature, even though 10 wt.% TiO_2_ reduces the degree of crystallinity to 9.04%, and the batteries with PEO-based electrolytes perform only above ambient. In addition, it should be noted that the choice of LiTFSI with PVDF is not necessarily the best. Investigation of the interface between lithium and PVDF-based electrolytes has shown that much better results can be obtained with LiFSI-PVDF than with LiTFSI-PVDF electrolyte, owing to the formation of the most stable 20 nm thick LiF–Li_x_SO_y_ sulfur compounds-LiOH-Li_2_CO_3_-Li_2_O mosaic interface between lithium and LiFSI [214].

Huang et al. used BF_3_ as an initiator and LiClO_4_ as the lithium salt to form a poly-tetrahydrofuran (PTHF) polymer electrolyte that uniformly filled the 3D framework of a cellulose mechanical support [215]. The addition of BF_3_ resulted in two beneficial effects: (a) It triggered the ring-opening polymerization of THF to form PTHF; (b) it coordinated with ClO_4_^−^ to increase the lithium transference number *t*_Li+_. Importantly, this PTHF-based solid electrolyte (PTSPE) was prepared by in situ polymerization of a precursor solution (0.6 mol L^−1^ LiClO_4_ and 0.6 mol L^−1^ BF_3_ dissolved in THF), which yielded much better electrochemical properties than those of commercial PTHF. The in situ PTSPE revealed a transference number *t*_Li+_ = 0.36, but its ionic conductivity of 2.3 × 10^−4^ S cm^−1^ at 60 °C was too low for it to be used at room temperature. At 60 °C, the Li/PTSPE/LiFePO_4_ cell delivered a capacity of 153 mAh g^−1^ at 0.1C, with the capacity retention being 91.3% after 100 cycles.

Such results illustrate the different strategies used presently to find solid electrolytes based on polymers: One is to increase the conductivity either by modification of existing polymers or by synthesis of new polymers, or by mixing them. Another solution involves adding small quantities of liquid electrolytes to form gel polymers, sometimes of the order of a few microliters per cell, just to reduce the interface resistance. Other efforts are intended to reinforce the mechanical properties, which can be achieved either by combining them with solid-state electrolytes in the form of pellets (Section 2) or by filling the polymers with nanosized ceramics. The progress of these different strategies is reviewed in this section. In practice, several of these strategies are used simultaneously to obtain the best results, but for clarity of presentation, they are reported in different subsections to outline these different aspects.

### 3.1. Electrolytes that Work at Room Temperature

Owing to the progress made in the synthesis of highly conductive polymers, some of them can now be possibly used as electrolytes in batteries that work at room temperature. In this context, a solid-state polymer electrolyte with an interpenetrating poly(ether−acrylate) (ipn-PEA) network was developed by Zeng et al. via photopolymerization of ion-conductive PEO and branched acrylate to obtain a rigid-flexible structure. This electrolyte revealed the bifunctionality of a high ion conductivity (0.22 mS cm^−1^) at room temperature and a high mechanical strength (ca. 12 GPa) as a result of the ideal combination of plasticity and rigidity, respectively, which were inherited from PEO and PEA. It was stable up to 4.5 V vs. Li^+^/Li. The Li/ipn-PEA/LiFePO_4_ cell at room temperature delivered capacities of 141 and 66 mAh g^−1^ at 0.5C and 5C, respectively [216]. A novel non-flammable SPE was proposed by Li et al. [217] that consisted of interpenetrating rigid-flexible poly (arylether-ketone) that was nonwoven and cross-linked with poly(ethylene glycol) dimethacrylate (average *M*_w_ = 900 g mol^−1^) to transport lithium ions. The ionic conductivity reached 1.2 × 10^−3^ S cm^−1^ at room temperature, the decomposition voltage was higher than 4.5 V, and it showed no noticeable volumetric expansion or contraction at 80 °C. Owing to the high ionic conductivity, the solid-state battery with this electrolyte works at room temperature. With LiFePO_4_ cathode, it delivered capacities of 124 and 116 mAh g^−1^ after 200 cycles at 0.5C and 1C, respectively, which were more than 90% percent of the corresponding initial values. Poly(ethylene glycol) diamine-based gel polymer electrolyte was used as an electrolyte in a flexible cell with LiFePO_4_ deposited on a carbon cloth as the cathode, and the anode was fabricated by stacking TiO_2_ and Ti_3_C_2_ that were coupled at the molecular level [218]. The flexible cell delivered a capacity of 84 mAh g^−1^ at the current density of 2 A·g^−1^. This cell, with a power density of 1412 W kg^−1^ and an energy density of 59 W kg^−1^, was stable during mechanical deformation and self-healing cycles. Another polymer for room temperature all-solid-state batteries is poly(ethyleneglycol)-borate ester (B-PEG) that was further covalently bonded with crosslinked silicon-doped poly(ethylene glycol) (PEG) [219]. The resulting hybrid polymer exhibited the highest ionic conductivity of 1.6 × 10^−4^ S cm^−1^ at room temperature and a transference number *t*_Li+_ = 0.68 for a B-PEG content of 23.1%. The Li//LiFePO_4_ cell with this hybrid SPE at 25 °C delivered a capacity of 120 mAh g^−^ at 0.5C. At 1C, the capacity was 90 mAh g^−1^, with the capacity retention being 84% after 100 cycles.

**Figure 7:**
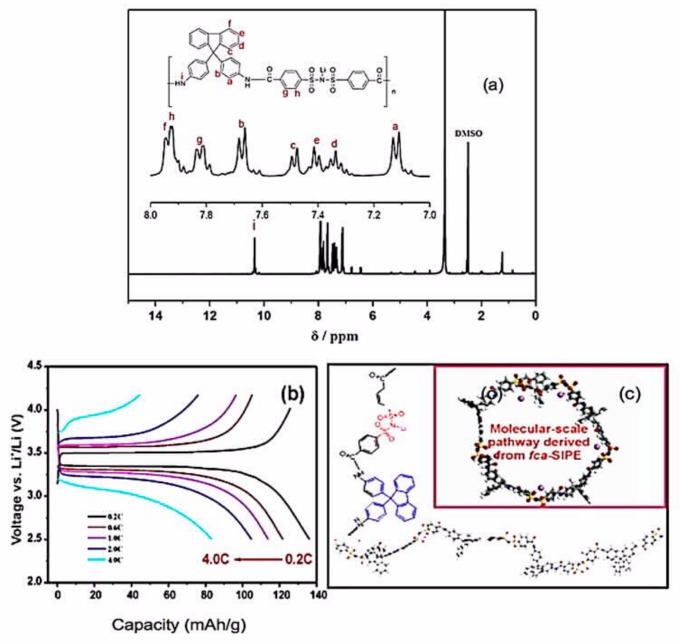
(**a**) ^1^HNMR spectrum of LiPFD. (**b**) Galvanostatic charge–discharge curves of the LiFePO_4_|PVDF-HFP/LiPFD membrane/Li cell at various C-rates at room temperature. (**c**) Graphical illustration of the free volume constructed from the bulky rigid fluorene cardo groups for revealing the nanoscale lithium-ion transport pathways in LiPFD (a cardo fully aromatic single-ion conducting polymer). Reproduced with permission from [221]. Copyright 2017 Wiley.

Liu et al. overcame the difficulty in preparing polymethyl methacrylate (PMMA) by using phase inversion method. They employed a highly viscous siloxane after hydrolysis of (vinyltrimethoxysilane) and succeeded in preparing a polymer membrane based on poly(methyl methacrylate-polyhedral oligomeric silsesquioxane) (P(MMA-POSS)) copolymer [220]. This polymer showed an ionic conductivity of 3.41 mS cm^−1^ at room temperature, an electrochemical stability window that extended up to 5 V vs. Li^+^/Li, and excellent compatibility with lithium. Although the transference number was limited to 0.49, the Li//LiFePO_4_ cell using this membrane with the optimum amount of POSS (10 wt.%) delivered a capacity of 151.9 mAh g^−1^ at 0.2C and a capacity retention rate of 99.8% at 0.5C after 100 cycles. It should be noted that PMMA usually forms electrodes that suffer from brittleness and peeling-off of the electrode layers for reasons that have been discussed elsewhere [100]. The performance of the cell prepared with P(MMA-POSS) suggests that the POSS component of the copolymer helped address this problem. Zhang et al. fabricated lithiated poly (4,4’-(9-fluorenylidene) dianiline)-co-(4,4’-dicarboxyl diphenyl sulfonimide) (LiPFD), a fluorine-containing cardo fully aromatic single-ion conducting polymer electrolyte (*fa*-SIPE), which was blended with PVDF–HFP (Figure 7) [221].

Owing to the free volume generated by the fluorene group, the ionic conductivity reached 6.2 10^−4^ S cm^−1^ at room temperature, with a remarkable transference number of *t*_Li+_ = 0.92 being obtained. Owing to this high *t*_Li+_ and a low interfacial resistance (81 Ω) that were attributed to the porosity of the membrane, the LiFePO_4_//Li cell with this electrolyte delivered a capacity of 134 mAh g^−1^ at room temperature, good power density up to 4C, and no capacity decay at 1C over the 140 cycles that it was tested. Note that usual mixing or stirring always leads to inhomogeneous mixing of the polymers, which obstructs the Li^+^ path and limits the ionic conductivity. To avoid this issue, Zhang et al. proposed an in situ polymerization process in which the precursors of the *fa*-SIPE are dissolved in PVDF–HFP prior to polymerization [222]. The in situ PVDF–HFP/*fa*-SIPE obtained after polymerization of the solution is then impregnated with ethylene carbonate (EC)/dimethyl carbonate (DMC) (v:v, 1:1) solvent to obtain an ionic conductivity of 0.93 mS cm^−1^ at 25 °C and 3.72 mS cm^−1^ at 80 °C, with *t*_Li+_ = 0.88 at room temperature. The Li//LiFePO_4_ cell employing this electrolyte delivered a capacity of 125 mAh g^−1^ at 2C over 250 cycles. Another single-ion polymer electrolyte was obtained with a poly(arylene ether) based polymer (LiPHFE) that was blended with PVDF–HFP [223]. Its ionic conductivity was 0.41 mS cm^−1^ at room temperature and 1.2 mS cm^−1^ at 80 °C. This electrolyte was tested with a LiFePO_4_ cathode. The half-cell delivered a capacity of 100 mAh g^−1^ at 1C over 800 cycles at room temperature. Note, however, that the cathode was wetted by an EC and DMC mixture (1:1, v/v) before stacking on the polymer film to obtain this result.

The number of polymers that are conductive enough to allow their use in lithium-batteries that work at room temperature is limited. As the ionic transportation of SPEs is generally coupled with the segmental motion of the polymer segments in the amorphous phases, many efforts are currently devoted to the design and synthesis of polymer matrices with the aim of preparing non-crystallized polymers with low glass transition temperatures [224,225]. That is why efforts are also taken to prepare GPEs in parallel to the efforts on SPEs. The problem in this case is finding a way to avoid the leaking of electrolyte (which results in the advantage of solid electrolytes being lost) and maintain the mechanical properties high enough to avoid the formation of dendrites. In this regard, Li et al. proposed a tri-layer, DF/L-PMMA/PVDF, where L-PMMA is linear-polymethyl methacrylate, capsuled by cross-linked PMMA. This structure was able to trap an electrolyte consisting of a solution of LiTFSI in 1-ethyl-3-methylimidazolium TFSI [226]. Owing to the large uptake of this liquid electrolyte (296%), the ionic conductivity increased to 1.18 × 10^−3^ S cm^−1^ at 25 °C. The LiFePO_4_//Li cell with this GPE delivered a capacity of 150 mAh g^−1^ at 25 °C and 0.1C, with the capacity retention being 97% over 50 cycles. The capacity was 110 mAh g^−1^ at 1C. The result shows that the fibrous CL-PMMA was able to retain the liquid electrolyte, at least at the scale of the 50 cycles that have been explored. Guo et al. proposed a porous polymer electrolyte containing PVDF and a grafted polymer that was synthesized by a simple method based on sulfonated polystyrene and the monoamine-terminated PEO derivative M2070 [227]. The free-standing membranes thus obtained were impregnated with 1 mol L^−1^ LiClO_4_-EC/PC v/v=1:1) electrolyte solution. The thus-obtained GPE with 30 wt.% grafted polymers showed the best results, with an ionic conductivity of 3.05 × 10^−3^ S cm^−1^ at room temperature. The electrochemical stability extended up to 4.8 V. The corresponding Li/GPE/LiFePO_4_ delivered a capacity of 141 mAh g^−1^ at 0.1C and retained 120 mAh g^−1^ after 130 cycles. A single-ion polymer was fabricated by grafting 4-amino-4’-trifluoromethyl bis(benzene sulfonyl)imide on the side chains of poly(ethylene-alt-maleic anhydride) with a grafting proportion of 50% [228]. After blending with PVDF–HFP, the membrane showed an ionic conductivity of 0.1 mS cm^−1^ at room temperature and 0.35 mS cm^−1^ at 80 °C. The transference number was *t*_Li+_ = 0.92. These data are comparable to the results for another single-ion polymeric electrolyte obtained by the polycondensation reaction between 4,4’-dicarboxyl bis(benzene sulfonyl)imide and 4,4’-amino bis(benzene sulfonyl)imide [229]. This membrane was soaked with EC/PC to obtain a GPE. The corresponding a LiFePO_4_/GPE/Li cell delivered 100 mAh g^−1^, without a significant capacity loss over 1000 cycles. The lower capacity compared with the results in reference [226] was attributed to the lower ion exchange capacity (1.6, against 2.9 mmol g^−1^).

### 3.2. Polycarbonate-Based Electrolytes

Polycarbonates (PCAs) have been reported to be an alternative polymer matrix for SPEs [230,231]. The use of PCA-based polymers is motivated by their high transference numbers [232,233,234,235]. A review of the PCA-based SPEs and their properties can be found in reference [236]. Among PC-based polymers, PPC is the most conductive, which allows its use in the electrolytes of Li//LiFePO_4_ cells at room temperature. The PPC-based all-SPE first proposed by Zhang et al. showed remarkable properties [234]. In this pioneering work that motivated further investigations on PCA-based solid electrolytes, an LFP//Li cell at room temperature using a 75 ± 5 μm thick cellulose supported PPC SPE (CPPC-SPE) delivered 142 mAh g^−^^1^ at 0.1C. At 0.5C, the cell delivered a capacity of 116 mAh g^−1^, with the capacity retention being 95% after 1000 cycles. A composite electrolyte consisting of PPC, LiTFSI, and LLZTO was proposed by Huo et al. [237]. Such a free-standing PPC-LiTFSI-Li_6.75_La_3_Zr_1.75_Ta_0.25_O_12_ all-solid-state composite electrolyte for a flexible ambient-temperature solid lithium battery was fabricated by Zhang et al. [232]. This electrolyte was stable up to 4.6 V. The corresponding LFP//Li cell delivered a capacity of circa 130 mAh g^−1^ at 1C, with 95% of the discharge capacity being retained after 200 cycles at room temperature. If, however, we consider the use of LLZTO for mass production of lithium-ion batteries, we should note that tantalum is rare and costly [238].

He et al. used another PCA-based polymer, namely poly(ethylene carbonate) (PEC), to synthesize a flexible garnet-based composite solid electrolyte composed of cubic nanosized aluminum-doped LLZO, PEC, P(VdF-HFP), and LiTFSI [239]. P(VdF-HFP) was used here to reinforce the flexibility. LiFePO_4_/Li all-solid-state batteries using the optimized composite electrolyte delivered a capacity of 121.4 mAh g^−1^, with retention being 96.3% at 1C after 100 cycles at 55 °C.

The other PCA-based GPEs, like poly(trimethylene carbonate) [240,241], poly(trimethylene carbonate) and poly(ε-caprolactone) copolymers [242,243], poly(ethylene carbonate) [244,245], poly (vinylene carbonate) [246], carbonate-linked PEO [247], poly(propylene carbonate allylglycidyl ether) [248], poly(heptamethylene carbonate) [249], and interpenetrating network of poly(diethylene glycol carbonate) (IPN-PDEC) [250], have been considered as possible polymers for all-solid-state lithium batteries that work only above ambient temperature, because of the lower ionic conductivities. In particular, an all-solid-state Li//LiFePO_4_ cell with SPEs composed of PCA-based polyurethanes and LiTFSI exhibited remarkable properties at 80 °C: A discharge capacity of 161 mAh g^−1^ at 0.2C, whereas at 1C, the cell delivered 134 mAh g^−1^ with 91% retention after 600 cycles [236]. This result is attributed to the use of PCA to bring soft segments that can dissolve the lithium salts and favor the transportation of the lithium ions [251], while the hard segments, which are needed to increase the mechanical strength and avoid dendrites on the lithium anode, was brought by the diisocyanates and short-chain diamines of polyurethane. This electrolyte (with 10 wt.% hard segments, 90% soft segments, and 20 wt.% LiTFSI) is gifted, with an electrochemical window of up to 4.5 V vs. Li^+^/Li and a lithium ion transference number of 0.45 at 80 °C. This result illustrates that, compared to those of PEO-based SPEs, PCA-based SPEs display higher lithium ion transference numbers and ionic conductivities, wider electrochemical windows, and better compatibility with lithium metal anode. They yielded better results than earlier polyurethanes based on different soft segments such as jatropha-oil [252], PEG [253], and poly(tetramethylene oxide glycol) [254]. For comparison, a Li//LiFePO_4_ cell at the same temperature of 80 °C with a SPE composed of thermoplastic polyurethane, PEO, and LiTFSI delivered capacities of 154 mAh g^−1^ at 0.2C and 127 mAh g^−1^ at 1C, with the capacity retention being 94% after 100 cycles at 1C [255]. Even though these results are better than those obtained with many solid electrolytes, the results obtained with PCA-based GPEs are better than those obtained for PEO-based polyurethane SPE. One reason is that PCAs exhibit good salt solubilities because of the carbonate group (–O– (C=O)–O–) being typically highly polar and containing Li^+^-coordinating oxygen [256]. In addition to plasticizing the polymer, the high dielectric constant of PC assists in the dissociation of the lithium cations and the covalently bonded anions, so that it increases the fraction of mobile ions [257]. Note, however, that the electrolytes based on polymers in the presence of PC are actually GPEs owing to the presence of PC, rather than SPEs. Porcarelli et al. prepared cross-linked electrolytes by copolymerization of PEG dimethacrylate (PEGDM), methyl ether methacrylate (PEGM), and lithium 1-[3-(methacryloyloxy)-propylsulfonyl]- 1-TFSI (LiMTFSI) in the presence of PC [258]. For the optimum composition of LiMTFSI, PEGM, PEGDM, and PC of 9:36:5:50 (in wt.%), the conductivity of this electrolyte reached 1.2 × 10^−4^ S cm^−1^ at 25 °C, with the transference number *t*_Li+_ = 0.86 at 25 °C. These composites were actually GPEs, owing to the presence of 50 wt.% of PC as the plasticizer. The half-cell with LiFePO_4_ positive electrode delivered a capacity of 126 mAh g^−1^ and was very stable at 0.5C over the 100 cycles that it was tested. To obtain a good rate capability, however, the cell has to work at a high temperature. At 70 °C, where *t*_Li+_ = 0.90, the capacity retention was 110 mAh g^−1^ at 2C.

### 3.3. Composite Polymer Electrolytes

Block/grafted copolymer electrolytes are part of another strategy of modulating the intrinsic structure of SPEs. In a pioneering work, Phan et al. suggested single-ion BAB triblock copolymers to be highly efficient lithium-metal electrolytes [259]. Following the same approach, densely grafted PEO brushes on a poly(hydroxylstyrene backbone and block copolymers with polystyrene were designed as model systems for lithium ion transport [260]. At 333 K, the ionic conductivity was approximately 6 × 10^−5^ S cm^−1^ and the modulus 2 × 10^6^ Pa for a composition of [EO]:[Li^+^] = 8:1. Another example is a self-doped solid block copolymer electrolyte, which combines a single-ion poly(lithium methacrylate-co-oligoethylene glycol methacrylate) ion conducting block and a structuring polystyrene block (PS) [261]. In this case, the conductivity is very attractive, but the compatibility with lithium has been tested only for five cycles, and no test with LiFePO_4_ or any other cathode has been conducted. On the other hand, single-ion triblock copolymer electrolytes based on linear PEO and side poly(lithium 1-[3-(methacryloyloxy)-propylsulfonyl]-1-(trifluoromethylsulfonyl)imide) (PMTFSI) blocks, PMTFSI−b-PEO−b-PMTFSI, were prepared by Porcarelli et al. and tested in a LiFePO_4_ cell at 70 °C [262]. Owing to the triblock polymer structure including MTFSI, which is beneficial to the mechanical properties, the copolymerization includes PEO units that increase the segmental mobility and, thus, the ionic conductivity, and the single anionic conduction process that increases the transference number *t*_Li+_ to a value close to unity. This cell at 70 °C delivered capacities of 150 mAh g^−1^ at 0.1C and 98 mAh g^−1^ at 0.5C. Moreover, the capacity retained at C/2 after 300 cycles was a high 80 mAh g^−1^. Recently, a polystyrene-poly(ethylene glycol)-polystyrene triblock copolymer with LiTFSI (EG: Li molar ratio of 20:1) was fabricated [263]. This solid membrane exhibited an ionic conductivity of 1.1 × 10^−3^ S cm^−1^ at 70 °C, a transference number of 0.17, a high degree of flexibility, good mechanical strength and thermal stability, good compatibility with lithium, and an electrochemical window that extended up to 4.5 V. At 70 °C, the Li//LiFePO_4_ cell with this electrolyte delivered capacities of 158 mAh g^−1^ at 0.2C and 127 mAh g^−1^ at 1C. The capacity retention at 0.2C was 91% after 120 cycles, which proved the good compatibility with LiFePO_4_.

Semi-interpenetrating polymer networks (s-IPN) include PMMA/polysiloxane-co- propyloxymethoxytriglycol [264] and poly(ethylene glycol) diacrylate (PEGDA)-co- poly-(vinyl chloride) (PVC)/PVDF-co-HFP [265]. Polymer electrolytes incorporating polymerized ionic liquids or other polymer systems having linear chains in s-IPN architectures were also investigated [265,266,267]. In all the cases, the role of the linear chain is to impart ionic conductivity and/or mechanical properties. A solid s-IPN-based polymer electrolyte membrane was prepared by mixing dimethacrylate monomer, PEO, and LiTFSI [268]. As usual, with polymers, the ionic conductivity increases with the concentration of LiTFSI, and the best conductivity exceeding 10^−3^ S cm^−1^ at 80 °C was obtained for a EO_m_/Li ratio of 16:1. With this 80 μm thick membrane as the electrolyte, the LiFePO_4_//Li cell delivered an initial capacity approaching 160 mAh g^−1^ at 0.1C. At 1C, the cell delivered 135 mAh g^−1^, with a capacity retention of 70% after 2000 cycles.

The self-assembly of trimethylated cyclodextrin (TMCD), PEO, and LiTFSI was investigated by Imholt et al. [269]. The methylation of the cyclodextrin (CD) increases its hydrophobicity and favors complexation. It also importantly increases the ionic conductivity of CD/PEO complexes, which reaches 10^−4^ S cm^−1^ at 100 °C for optimized γ-TMCD/PEO_5_LiTFSI (EO:Li ratio = 5:1). The transference number is 0.34, which is still small, but larger than those of many PEO-LiTFSI-based systems. At 60 °C, the LiFePO_4_//Li cell with this SPE delivered a capacity of 110 mAh g^−1^ at 1C, with the capacity retention being 95% after 200 cycles.

The PEO-LiTFSI composite has been the most studied and used because of its compatibility with lithium anode, among other properties. The drawback, however, is its slow oxidation above 3.9 V, which restricts its use to cells with LiFePO_4_ or LiV_3_O_8_ cathodes. To circumvent this problem, Zhou et al. recently fabricated a double-layer polymer electrolyte, in which the PEO-LiTFSI layer is used to contact only the lithium anode, while the other layer is a poly(N-methyl-malonic amide) (PMA)-LiTFSI layer that contacts only the cathode [270]. PMA contains a repeating unit of dimethylacetamide and is used as an additive to protect the electrolyte oxidation by a high-voltage cathode. Therefore, the PMA-LiTFSI layer was protected from side-reactions with the lithium anode by the PEO-LiTFSI layer, while the PEO–LiTFSI layer was protected from high-voltage oxidation by the PMA–LiTFSI layer, so that the electrolyte is suitable for a lithium-cell with cathode materials of the 4-volt class. Indeed, Li/PEO–LiTFSI/PMA–LiTFSI/LiCoO_2_ at 65 °C delivered 108.5 mAh g^−1^ after 100 cycles at 0.2C (100 μA cm^−2^), which amounted to 91.2% of the capacity at the fifth cycle. At 1C, a stable capacity of 57 mAh g^−1^ was obtained. Recently, a nanocomposite cathode LiCoO_2_-LLZO was fabricated by using a block copolymer template containing precursors of both LiCoO_2_ and LLZO [271]. The corresponding half-cell again utilized the PEO-LiTFSI layer as the separator and delivered a capacity of 98.2 mAh per gram of electrode at C/24 at room temperature. The cyclability has been tested for 20 cycles only. At least, over these 20 cycles, the capacity remained constant. These results are therefore promising, and the capacity per gram of the electrode is actually larger than those of most solid-state batteries, but the cell remains to be tested for longer times and at different rates. Another strategy used to prepare PEO-based polymers that are compatible with LiCoO_2_ involves coating the LiCoO_2_ particles with the electrochemically oxidation resistant poly(ethyl cyanoacrylate) (PECA) through in situ polymerization [272]. The researchers used lithium difluoro(oxalato)borate (LiDFOB) as the lithium salt instead of LiTFSI and found that the PECA coating reduced the continuous decomposition of LiDFOB in PEO electrolyte, which improved the cycling ability of the PECA-coated LiCoO_2_/PEO-LiDFOB/Li battery. These results illustrate the importance of the thermodynamic driving force for the decomposition at interfaces in all-solid lithium batteries owing to the limited electrochemical window of the solid electrolyte materials and their poor chemical compatibility with the electrodes. This driving force and the mechanisms of applying interfacial coating layers to stabilize the interface have been studied by first-principle computations [273].

**Figure 8:**
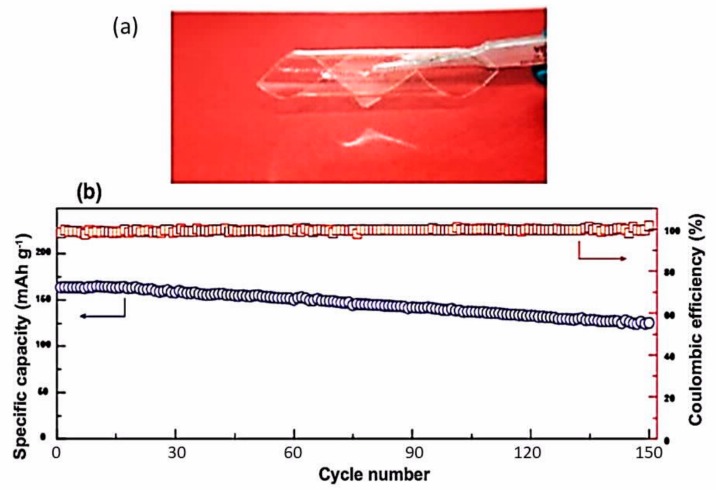
Membrane obtained after curing a liquid mixture of poly(ethylene glycol) diacrylate (PEGDA) and poly(ethylene glycol) diglycidyl ether (PEGDE) with lithium bis-(trifluoromethanesulfonyl)imide (LiTFSI) and benzoyl peroxide (BPO) at 110 °C for 2 h. The mass ratio of PEGDE:PEGDA was 1:1 and the molecular weight of PEGDE 500 g mol^−1^. The chain length of PEGDA was determined by its molecular weight of 1000 g mol^−1^. (**a**) This membrane could be bent without cracking. (**b**) The cycling performance of LiFePO_4_//Li battery with this membrane as the electrolyte. The test was conducted at 0.2C and 55 °C. Reproduced with permission from [274]. Copyright 2018 Elsevier.

An in situ plasticized SPE with a double-network was synthesized by polymerization of PEGDA and poly(ethylene glycol) diglycidyl ether (PEGDE) (PEGDE:PEGDA mass ratio was 1:1), with 1 mol L^−1^ LiTFSI and benzoyl peroxide (BPO; 1 wt.%) as the lithium salt and initiator, respectively [274]. The best mechanical and ionic conducting properties were obtained for the molecular weight (or chain length) of PEGDA equal to 1000 g·mol^−1^, in which case the ionic conductivity was 5.3 × 10^−5^ S cm^−1^ at 30 °C. The explanation given by the authors is that the short chains among the cross-links are tethered, with severely restricted conformational rotations, which result in higher glass transition temperatures. In addition, the electrochemical stability was up to 4.7 V. When used as an electrolyte in an LFP//Li cell at 55 °C, a discharge capacity of 162 mAh g^−1^ was obtained, and the retention was 125 mAh g^−1^ after 150 cycles at 0.2C (Figure 8). Moreover, the cell exhibited excellent mechanical flexibility. This good result is an additional demonstration of the efficiency of in situ polarization to stabilize the electrode-electrolyte interface.

A super soft polymer matrix containing polyether side moieties called “jeffamine” [275] combined with LiTFSI as the source of Li^+^ was proposed recently. Its compatibility with lithium was demonstrated, and, when used as an electrolyte in Li//LFP cells, it delivered decent specific/areal capacity with good Coulombic efficiency between room temperature and 70 °C.

To solve the problem of contact between the cathode and the electrolyte, which is a major issue in all-solid-state batteries, Zhang et al. proposed a method of adding a GPE to the cathode in advance during the preparation process [276]. According to this process, a liquid electrolyte (1 mol L^−1^ LiPF_6_ in EC/DEC 1:1 by volume), PEGDA monomer, a photoinitiator, and LFP were mixed with acetonitrile. The composite GPE@LFP cathode was obtained after the evaporation of acetonitrile, and the polymerization of PEGDA monomers conducted under ultraviolet (UV) irradiation. The electrolyte was prepared by in situ polymerization of the liquid electrolyte + PEGDA monomers that infiltrated a cellulose membrane. The corresponding LFP//Li cell revealed a capacity retention of 94.7 mAh g^−1^ at 1C after 200 cycles, which corresponded to approximately 77.6% of the discharge capacity of the first cycle.

Perfluoropolyether (PFPE)-based liquid electrolytes with LiTFSI exhibit multifunctional properties owing to a unique anion-solvent interaction [277]. This prompts further research on solid PFPE-based electrolytes, as they are not limited in the anodic domain. Fluorinated solid PFPE/LiTFSI was synthesized with urethane methacrylate end-groups [278] but have not yet been tested as electrolytes. Finally, we note that the polymers and copolymers that compose the electrolytes that we have mentioned are linear. However, the unique topology of star polymers allows for a higher mobility in the outer sphere of the arms, which suggests promise for application in SPEs. Wang et al. were the first to propose a hyper-branched star liquid crystal polymer as an all-solid-state polymer electrolyte for LIBs [279]. To combine the advantages of liquid crystals and star polymers, Wang et al. synthesized a hyper-branched star liquid crystal polymer as an all-solid-state polymer electrolyte for lithium-ion batteries [280]. Triphenylene (a discotic liquid crystal) was selected as the core of a six-arm star polymer that was synthesized via sequential atomic transfer radical polymerization of styrene and poly(ethylene glycol) methyl ether-methacrylate (PEGMA). The film composed of the six-arm copolymer and LiTFSI constitutes a new solid electrolyte with an ionic conductivity of 1.46 × 10^−4^ S cm^−1^ at 30 °C, a transference number of 0.37, and an electrochemical window extending up to 5.1 V. The LiFePO_4_//Li cell with this electrolyte at 60 °C delivered a capacity of 139 mAh g^−1^ at 0.1C, which was maintained at 130 mAh g^−1^ after 50 cycles and 127 mAh g^−1^ at 0.2C. This result is promising if we consider that it is the first time that a six-arm polymer and a discotic ionic liquid are considered for application as an electrolyte in solid-state batteries.

### 3.4. Polymer Electrolytes with Ceramic Fillers

The high specific gravity of LLZO is a penalty for realizing a high energy density of a full cell. This can be avoided by inserting any ceramic filler such as LLZO in the polymer matrix in the form of nanoparticles in low concentrations. The nanowire morphology of LLZO is of particular interest, because it forms a percolating network of highly conducting materials with minimum weight penalty [281]. Pre-percolated continuous microstructures can importantly enhance the lithium-ion conductivity without introducing much weight [282]. In addition, such a LLZO network reinforces the mechanical properties needed to suppress the formation of lithium dendrites [283]. For instance, LLTO nanowires importantly increase the ionic conductivity of polyacrylonitrile (PAN)–LiClO_4_ at room temperature [170,194]. In particular, by incorporating only 5 wt.% of the ceramic filler comprising LLZO nanowires that were prepared by electrospinning in PAN, the room temperature ionic conductivity of a PAN-LiClO_4_-based composite was increased by three orders of magnitude to 1.3 × 10^−4^ S cm^−1^ [284]. Note that this LLZO concentration was the optimum value, as the conductivity decreased when the wt.% of the filler was increased further. The optimum concentration of 5 wt.% also refers to that of LLZTO particles, the sizes of which are centered at 30 nm, which leads to the highest ionic conductivity (5.2 × 10^−4^ S cm^−1^ at 20 °C) of the PPC-LiTFSI-Li_6.75_La_3_Zr_1.75_Ta_0.25_O_12_ all-solid-state electrolyte [231]. Following the same strategy, a membrane composed of LLZO particles and PVDF–HFP polymer matrix was proposed by Zhang et al. [285]. Sun et al. proposed a new composite based on LLZO and PVDF:LiClO_4_ observing a high ionic conductivity at room temperature (2.6 × 10^−4^ S cm^−1^ at 20 °C) [286]. The results are in agreement with the work proposed by Zhang et al. in 2017 [287]. Owing to the addition of LLZO particles, the transference number *t*_Li+_ in this HSE increased to 0.61, because the local chains of the polymer can be relaxed, and the segment motion is promoted due to the interaction of inorganic fillers and polymer chains. At 0.5C, the cell Li/HSE/LiFePO_4_ delivered a reversible discharge capacity of 113 mAh g^−1^, with a capacity retention of 92.5% over 180 cycles. A porous PVDF–HFP polymer was also impregnated with a liquid electrolyte to form a gel interlayer between the electrodes and the garnet particles (calcium- and niobium-doped LLZO) [288]. The corresponding Li//LiFePO_4_ cell delivered 140 mAh g^−1^ at a current density of 170 mA g^−1^, which was very stable over the 70 cycles that the cell was tested.

The mechanism behind the ionic conductivity displayed by LLZO-PEO-LiTFSI is debated. While some NMR experiments suggest a preferential conductivity via doped LLZO [232], others suggest that the conductivity is because of the PEO [289]. This suggests that the conductivity is very much dependent not only on the amount of LLZO inserted into the matrix, but also on the morphology of the LLZO particles. In particular, the beneficial effect of nanosized LLZO particles (and ceramic particles in the polymer electrolyte in general) suggests an effect of the interface between the garnet and the polymer, which is maximized by the nanosize. This is consistent with an additional transport mechanism at the interface between LLZO and P(EO)_20_-LiClO_4_ that was revealed by impedance spectroscopy analysis [290]. Actually, the fact that the enhancement in the conductivity upon the introduction of ceramic fillers to polymers is due to the space charges at the ceramic/polymer electrolyte interface has been recognized since decades [291]. These considerations can be summarized in a sentence by Takada et al.: “Understanding and controlling the interfacial ionic transport will pave the way to solid-state batteries that are superior to conventional liquid-electrolyte systems” [292].

**Figure 9:**
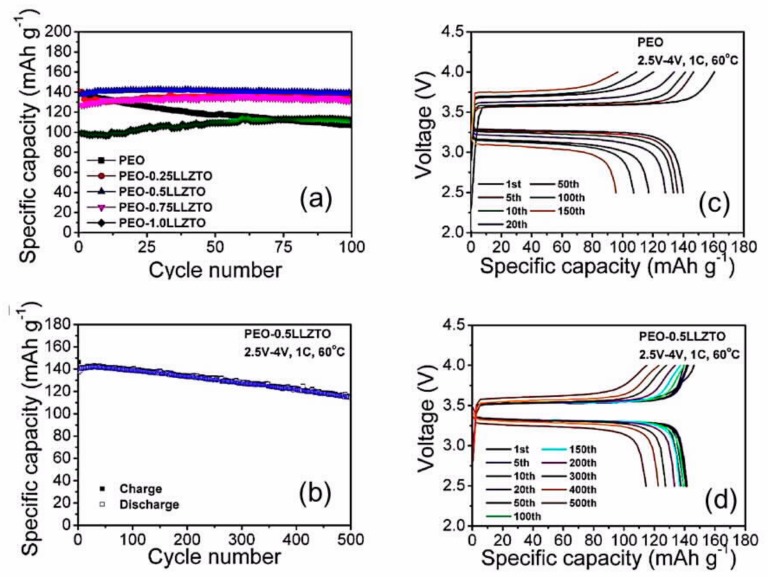
Electrochemical properties of a LiFePO_4_//Li cell with poly(ethylene oxide) (PEO)-LLZTO electrolyte cycled at 60 °C at the rate of 1C for different LLZTO concentrations. (**a**) Capacity retention after initial 100 cycles of the cells. (**b**) Cycle performance of the LFP/PEO-0.5LLZTO/Li cell. (**c**,**d**) Charge/discharge profiles of the cells LFP/PEO/Li and LFP/PEO-0.5LLZTO/Li, respectively. Reproduced with permission from [300]. Copyright 2017 Elsevier.

Composite electrolytes made of LLZTO and a polymer like PEO afford good results. However, owing to PEO, the ionic conductivity is still too low to operate the battery at room temperature, and the cells in this case were tested at 55–60 °C [150,194]. PEO-based composite cathode layers (filled with LiFePO_4_ particles) ≈300 μm in thickness and composite electrolyte layers (filled with aluminum-doped LLZTO particles) were stacked layer-by-layer with lithium foils as the negative layer and hot-pressed into a monolithic all-solid-state LIB. When tested at 60 °C, the cell delivered a capacity of 155 mAh g^−1^_,_ but the tests were limited to 10 cycles only [293]. Solid-state LiFePO_4_//Li batteries with the electrolytes of “ceramic-in-polymer” and “polymer-in-ceramic” PEO-LLZTO-LiTFSI delivered capacities of 139.1 mAh g^−1^, with a retention of 93.6% after 100 cycles at 0.2C and 55 °C, and 127 mAh g^−1^, after 200 cycles, respectively [294]. This is quite comparable with the result obtained with the PEO/LiTFSI+LATP/PAN nanofiber electrolyte (144 mAh g^−1^ after 100 cycles under 0.2 C at 60 °C) [295]. The SPE-LLZTO-SPE electrolyte was fabricated by Chi et al., where the SPE is LiTFSI/PEO at a concentration ratio of 8:1 [296]. The LiFePO_4_/SPE-LLTZO-SPE/3D-Li cell, where 3D-lithium is the lithium anode prepared in a particular way (described in reference [297]) to restrain the growth of lithium dendrites, was tested at 90 °C. At a rate of 0.2C, the cell delivered a capacity of 140 mAh g^−1^, which was stable at 135 mAh g^−1^ after 200 cycles, with a Coulombic efficiency of 99.6%. In another work, PEO-LLZO-LiTFSI, where LLZO was incorporated in the form of nanowires, was used as the electrolyte in a LiFePO_4_//Li cell that delivered a capacity of 158.8 mAh g^−1^ after 70 cycles under 0.5C at 60 °C [298]. However, anions are more mobile than the cations in PEO-LiTFSI polymer electrolytes, which results in their lithium-ion transference numbers being usually lower than 0.5 [299]; therefore, the choice of this composite may not be the best. However, the same PEO-LLZO-LiTFSI electrolyte where LLZO was incorporated in the form of nanofibers was tested earlier in a symmetric Li/PEO-LLZO-LiTFSI/Li cell, where the membrane could effectively suppress the dendrites when the cell was operated at a current density of 0.2 mA cm^−2^ for approximately 500 h and at a current density of 0.5 mA cm^−2^ for over 300 h [195]. We have already mentioned the association of LLTO nanowires in PAN–LiClO_4_ at room temperature [170,194]. The LLZTO filler in the PEO/LiClO_4_ matrix was investigated as a composite electrolyte for a lithium solid-state battery with LFP cathode (Figure 9) [300].

For the composition of PEO:LiClO_4_:LLZTO of 60.20:9.69:30.10 (wt.%), the cell at 60 °C and 1C rate delivered a capacity of 140 mAh g^−1^, with the capacity retention being 83% after 500 cycles. LLZTO nanoparticles dispersed in PEO were used by Zhang et al. to operate lithium cells with LFP and LiFe_0·15_Mn_0·85_PO_4_ (LFMP) cells at 60 °C [150]. Both the cells were operated for more than 200 cycles at 0.1C with the capacity retention being 90%. At this temperature, the cells delivered energy densities of 345 Wh kg^−1^ (662 Wh L^−1^) with LFP and 405 Wh kg^−1^ (700 Wh L^−1^) with LFMP (without considering the package weight or volume). Then, Huo et al. investigated the same cells, except that they added an ionic liquid to wet the interface with LLZTO [301]. Owing to the gain in conductivity of one order of magnitude with the wetting, the cell could be used at room temperature, however, a capacity retention less than 90% was observed after 100 cycles at 0.1C. In these works, the advantage of the large electrochemical window of LLZTO was exploited to use a 4+ V cathode (LFMP). In the same way, a combination of LiMn_0·8_Fe_0·2_PO_4_ and LiMn_0·85_Fe_0·1_Mg_0.05_PO_4_ with Li_4_Ti_5_O_12_ anode was used with a thin LLZO-based hybrid electrolyte layer. At 60 °C, the good cycling and rate capability are compatible for the development for low-voltage systems in industry [302,303].

The cross-linked poly(ethylene glycol) methyl ether acrylate (CPMEA) polymer and LATP ceramic were used to build a polymer/ceramic membrane/polymer sandwich structure that was used as an electrolyte in an all-solid lithium battery with LiFePO_4_ cathode. The capacity retention of Li/LiFePO_4_ using this electrolyte was still approximately 102 mAh g^−1^ at 0.6C (0.51 mA cm^−2^) after 640 cycles at 65 °C [304]. Here, LATP has been chosen because it has advantages over other inorganic materials in terms of the high lithium-ion conductivity and chemical stability in air; the CPMEA layer adheres to/wets the lithium metal surface and renders the lithium-ion flux at the interface more homogeneous, which facilitates homogeneous deposition of lithium, which in turn prevents the formation of dendrites.

LATP has also been associated with PEO and boronized polyethylene glycol [305]. At 60 °C, the resulting membrane used as an electrolyte with lithium metal anode and LFP cathode delivered capacities of 158 and 94 mAh g^−1^ at 0.1C and 2C, respectively, but the life cycle was not investigated. A high conductivity with the PEO-LiClO_4_ system was obtained by adding 10 wt.% LATP nanoparticles, in which case the conductivity reached 1.7 × 10^−4^ S cm^−1^ at 20 °C owing to the transport of cations within the interphase region surrounding the particles, which achieved percolation at low nanoparticle loadings [211].

LAGP was also selected to disperse in the PEO-matrix without employing any other additive [158]. The PEO-20% LAGP hybrid electrolyte exhibited the maximum ionic conductivity of 6.76 × 10^−4^ S cm^−1^ and an electrochemical window of 0–5.3 V at 60 °C. The all-solid-state battery LiFePO_4_/Li fabricated with this electrolyte delivered 166, 155, and 108 mAh g^−1^ at 0.1, 0.2, and 1C, respectively, with the capacity retention being 90% after 50 cycles at 60 °C. A solid-state Li//LiFePO_4_ cell assembled with a HSE composed of 70 wt.% LAGP, 21 wt.% PEO-LiClO_4_, and 9 wt.% SCN delivered a high discharge capacity of 136.8 mAh g^−1^ over 100 cycles at 0.2C at room temperature [306]. The good result was attributed to the enhanced ionic conductivity due to an increase in the solvating power for dissolving lithium salts and a reduction in the crystallinity of the PEO phase upon the incorporation of SCN [307]. Actually, the existence of SCN as a plastic crystal and its high polarity are responsible for its good ability to dissolve various lithium salts [307]. The introduction of SCN into polymer-based electrolytes dates back many years and a review on these prior works can be found in reference [307]; the electrolytes thus obtained can be used only at room temperature at low C-rates (0.1C). To improve the performance at higher rates, SCN was introduced more recently into poly(DADMA)-TFSI, which is a pyrrolidinium-based PIL, with LiTFSI serving as the lithium salt [308]. 80% [50%PIL-50%SCN]-20%LiTFSI revealed an ionic conductivity of 5.74 10^−4^ S cm^−1^ at room temperature, an electrochemical window of 5.5 V, and Young’s modulus of 4.9 MPa. The Li//LiFePO_4_ cell with this electrolyte was able to deliver at 25 °C capacities of 131.8 and 121.2 mAh g^−1^ at 0.5C and 1C, respectively. Recently, a SCN-based solid-state plastic crystal electrolyte (PCE) was engineered as an interlayer to resolve the instability of sulfide electrolytes against lithium metal [309]. The PCE was composed of 5 mol% LiTFSI in SCN plus 2 wt.% LiNO_3_ additive. LGPS was chosen as the solid sulfide electrolyte. The LiFePO_4_/PCE-LGPS-PCE/Li battery delivered a capacity of 131 mAh g^−1^ at 0.5C, of which 122 mAh g^−1^ remained after 120 cycles at 0.5C.

The incorporation of sulfide electrolytes as an active filler into the PEO matrix has also been explored. We have already mentioned that PEO with 2% vol. Li_3_PS_4_ nanofiller when used as a hybrid polymer electrolyte in a LiFePO_4_ battery at 60 °C exhibited 80.9% capacity retention rate after 325 cycles, owing to the improvement in the mechanical properties and the good rate capability, which were attributed to the increase in conductivity [92]. Furthermore, microparticles of LGPS incorporated into PEO also act as fillers. At 60 °C, LiFePO_4_/PEO_18_-LiTFSI 1%LGPS/Li cell delivered 158 mAh g^−1^, with 92.5% capacity retention after 60 cycles at 0.1C [310]. It is difficult, however, to make a comparison between the results obtained with Li_3_PS_4_ and LGPS, because the improvement in performance associated with the filler usually requires nanosized particles, which lead to larger interfacial regions with the polymer and are more effective in preventing dense packing of the polymer segments than micron-sized fillers [150]. Note again that high cost realistically excludes germanium-based solid electrolytes including LGPS or LAGP at the industrial scale. Vinado et al. proposed to substitute germanium for tin to reduce the cost, and fabricated a cell with Li_10_SnP_2_S_12_ electrolyte, lithium–indium alloy anode, and (LCO) cathode through ALD of Li_3_NbO_4_ on LCO to improve the interfacial stability [311]. Indeed, Li_10_SnP_2_S_12_ displays the same conductivity as LGPS; nevertheless, despite the improvement with Li_3_NbO_4_ coating, the cyclability was limited. Note, however, that Li_10_SnP_2_S_12_ was used in the form of a cold-pressed pellet and not as a filler in the form of nanoparticles. In addition, attention must be paid to the interface with the lithium anode. In this context, Wang et al. fabricated an artificial layer by introducing an inorganic-organic hybrid interlayer (“alucone”) at the interface between the lithium metal and Li_10_SnP_2_S_12_ electrolyte by using molecular layer deposition [312]. Coupled with the LCO (not protected) cathode, the addition of this interlayer appreciably improved the performance of the cell, compared with that of the same battery without the alucone layer. The delivered capacity at 55 °C and 0.1C capacity of 120 mAh g^−1^ was maintained at 60 mAh g^−1^ after 150 cycles. One can thus expect good results by simultaneously protecting the LCO particles, as reported in reference [311], and inserting the alucone interlayer between Li_10_SnP_2_S_12_ and the lithium anode, as mentioned in reference [312]. This also illustrates the interest in molecular layer deposition [32].

SiO_2_ is another ceramic filler that has been used to increase the ionic conductivity. In particular, in situ synthesis of 12 nm diameter SiO_2_ nanospheres and PEO chains was achieved by hydrolysis of tetraethyl orthosilicate in a PEO solution [313]. The SiO_2_ filler facilitated the segmental motion of the polymer and improved the degree of LiClO_4_ dissociation, so that the ionic conductivity increased to 1.2 × 10^−3^ S cm^−1^ at 60 °C and 4.4 × 10^−5^ S cm^−1^ at 30 °C. The synthesis process plays an important role here. The *in situ* synthesis referred to in this paper makes it possible to homogenously mix the filler nanoparticles inside the polymer. On the other hand, when the ceramic-polymer composite electrolytes are synthesized by mixing preformed ceramic particles with polymers, not only does it result in an inhomogeneous distribution in some well-crystallized parts in the polymer, but it also leads to agglomeration of the nanoparticles. Another example of the efficiency of SiO_2_ filler is a hierarchical electrolyte (SiSE) fabricated via in situ polymerization of tripropylene glycol diacrylate monomer in the presence of a liquid electrolyte, which was absorbed in a SiO_2_ hollow nanospherical layer [314]. In this case, the quasi-solid SiSE was in situ fabricated on the cathode, and the absence of formation of dendrites on the lithium side resulted in an improved cycling stability of the LiFePO_4_/SiSE/Li cell.

## 4. Solid- and Quasi-Solid-State Batteries at Higher Potentials

### 4.1. Four-Volt Class Cells

As a compromise, an increase in conductivity can be achieved by combining a solid polymer with a low conductivity and a liquid electrolyte with a high conductivity to obtain a GPE. Moreover, as some of the polymers now display an electrochemical window that reaches 5 V, it is possible to switch from LiFePO_4_ to other inorganic cathodes with larger redox potentials relative to Li^+^/Li. In particular, in situ polymerized poly(ethylene glycol phenyl ether-acrylate) (PEGPEA)-based GPE combined with 1 mol L^−1^ LiPF_6_ in EC//DMC/ethyl methyl carbonate (EMC; 1:1:1 in volume) solvent was introduced by Niu et al. [315]. This GPE displayed a conductivity of 3.35 10^−3^ S cm^−1^ at 25 °C and an electrochemical stability up to 4.9 V vs. Li^+^/Li. The Li[Ni_0.5_Co_0.2_Mn_0.3_]O_2_ /PEGPEA-GPE/Li battery delivered a capacity of 155 mAh g^−1^ at 0.2C, with a capacity retention of 97.5% after 70 cycles (but has not been tested further), which makes it a promising GPE for lithium batteries.

PVDF-based polymers are currently used as a component of solid electrolytes, in combination with ceramics, to improve the mechanical properties. PVDF–HFP polymer including aluminum-doped LLTO covered with a modified SiO_2_ layer was used as an electrolyte in Li//LiCoO_2_ after activation in a solution of 1 mol L^−1^ LiPF_6_ in EC/EMC. The cell delivered an initial capacity of 153 mAh g^−1^ at 0.5C, with a capacity retention of 80.5% and a Coulombic efficiency of 99.2% after 500 cycles [316]. This GPE, stable up to 5 V vs. Li^+^/Li, was thus able to suppress the formation of dendrites. We will see later that this GPE (activated in a different liquid electrolyte) also afforded interesting results in Li-O_2_ cells for the same reason.

Nanowires of 5% palygorskite ((Mg,Al)_2_Si_4_O_10_(OH)) as new ceramic fillers in PVDF with LiClO_4_ salt were sufficient to increase the elastic modulus to 96 MPa and enhance the yield stress by 200% [317]. This illustrates the beneficial effect of the nanowire morphology, which produces a cross-linking network that is beneficial to the mechanical properties. In addition, the transference number increased to 0.54, owing to the interaction between palygorskite and ClO_4_^−^. Li//LiNi_1/3_Mn_1/3_Co_1/3_O_2_ cell with this electrolyte delivered a capacity that increased from 117.6 to 121.4 mAh g^−1^ during the first five cycles, which was maintained at 118.1 mAh g^−1^ after 200 cycles at 0.3C at room temperature.

Poly(vinylidene carbonate) with LiDFOB as the lithium salt incorporated into a cellulose nonwoven substrate constitutes a solid electrolyte with an electrochemical window extending up to 4.5 V and a transference number of 0.57 [246]. The corresponding Li//LiCoO_2_ cell at 50 °C delivered a capacity of 146 mAh g^−1^ at 0.1C, with a capacity retention of 84% after 150 cycles. At 0.5C, the discharge capacity was 73 mAh g^−1^.

PEO and PMMA blended with the copolymer PVDF–HFP were impregnated with a liquid electrolyte, 1.0 mol L^−1^ LiPF_6_/EC-DMC (1:1 v/v), which was chosen as the plasticizer to form a GPE. The corresponding cell with lithium anode and LiCoO_2_ cathode delivered a capacity of 52.7 mAh g^−1^ at a current density of 0.1 mA cm^−2^, with 98% capacity retention after 100 cycles [318].

Wang et al. proposed modification of the surface of lithium with PEO + LiTFSI to suppress the formation of dendrites upon combining with LAGP-PEO solid electrolyte [319]. Owing to LAGP, the oxidative decomposition potential of the electrode was increased to 5.2 V, which was much higher than that of PEO alone [320]. The all-solid-state cell with LiMn_0.8_Fe_0.2_PO_4_ positive electrode cycled in the voltage range 2.5–4.5 V, at 50 °C, delivered an initial capacity of 160 mAh g^−1^ at 0.1C, and stabilized at approximately 137 mAh g^−1^ over 200 cycles. As LAGP is not compatible with lithium metal, Wang et al. inserted a 500 nm thick LiPON thin film on the metal surface by RF magnetron sputtering of a Li_3_PO_4_ target. The corresponding Li(LiPON)/LAGP-PEO(LiTFSI)/LiFePO_4_ cell delivered a capacity close to 160 mAh g^−1^ over 150 cycles at 0.2C and 50 °C [321]. Addition of LLZTO rather than LAGP to PEO+LiTFSI helps get rid of the expensive germanium without altering the pinning of TFSI^−^. This composite electrolyte has been tested not only with LiFePO_4_, but also with LiNi_0.5_Co_0.2_Mn_0.3_O_2_ and lithium-metal anode, in which case a pouch cell was assembled to light the LED in both flat and bended states [322].

Recently, Chen et al. fabricated a new solid electrolyte that exhibits not only a good ionic conductivity, but also a high transference number *t*_Li+_ [323]. Instead of PEO, they chose PEC, which is known to display a high *t*_Li+_ owing to its single carbonate group (–O– (C=O)–O–). PEC and LiFSI were inserted into the interlayer of lithium montmorillonite (LiMNT). LiMNT is a single-ion conductor, which is needed to obtain a high *t*_Li+_. In addition, the carbonate group contains lone-pair electrons, and the resulting electrostatic interactions between PEC, LiFSI, and LiMT order the Li^+^ into the intercalation space, which increases the ionic conductivity. To increase it even further, a small quantity of fluorinated compounds has been added, namely high-voltage fluoroethylene carbonate (FEC) solvent and poly(tetrafluoroethylene) (PTFE) binder. The 30PEC-70LiMNT-80LiFSI-15FEC-3PTFE composite electrolyte showed an ionic conductivity of 3.5 × 10^−4^ S cm^−1^ and a transference number *t*_Li+_ = 0.83 at room temperature, and was stable up to 4.5 V vs. Li^+^/Li. Furthermore, a 3D lithium anode was chosen instead of the usual 2D lithium-foil in order to avoid the growth of lithium dendrites [297]. With a LiFePO_4_ cathode, the cell at room temperature delivered 146 mAh g^−1^ at 0.5C, with capacity retentions of 93.5% after 100 cycles and 91.9% after 200 cycles at 0.5C. The cell maintained a capacity of 108 mAh g^−1^ at 3C. With an Al_2_O_3_/LiNi_0.5_Co_0.2_Mn_0.3_O_2_ cathode, the cell cycled in the range 2.5–4.3 V and delivered an initial capacity of 150.7 mAh g^−1^ at 0.2C, which was maintained at 138.6 mAh g^−1^ after 100 cycles. The capacity at 1C was 138.6 mAh g^−1^. Note that fluorination has also been recognized as an important aspect for realizing a FEC-based electrolyte for Li//LiNi_0.6_Co_0.2_Mn_0.2_O_2_ cell, which was cycled at 1.5 mA cm^−2^ for more than 600 cycles, in relation to the formation of a SEI on the lithium anodes in this environment [324]. FEC was also be used as an additive that protected the lithium metal anode through the formation of a LiF-rich solid electrolyte interphase; DFT simulations have shown that an artificial SEI enriched in LiF salt promotes uniform lithium electrodeposition [325]. The Li//LiNi_0.5_Co_0.2_Mn_0.3_O_2_ cell with 5% FEC in EC-DEC electrolyte showed improved cyclability than a cell without FEC (although the capacity retention with 5% FEC was limited to 65% after 100 cycles at 1C) [326]. In the former case, the electrolyte was liquid, but it illustrates the role of FEC on the anode, which may also explain the performance of FEC-based solid electrolytes (mentioned earlier).

The combination of polymer + salt + ionic liquid has been considered to be promising for a long time for obtaining electrolytes with enhanced properties, because the ionic liquid is known to act as a plasticizer as well as a supplier of free ion charge carriers. Focusing on the results obtained recently, polymer electrolytes (PEO + 20 wt.% LiTFSI) + *x* wt.% 1-butyl-3-methylimidazolium bis(trifluoromethane sulfonyl) imide ionic liquid was investigated by Singh et al. [327]. The best results were obtained for *x* = 20 wt.%, in which case the ionic conductivity was 1.5 × 10^−4^ S cm^−1^ at 30 °C, with a transference number *t*_Li+_ = 0.27. The Li//LiMn_2_O_4_ cell with this electrolyte showed a stable cyclic performance. We shall later see an extension of this work for sodium-batteries.

In a different approach, Borzutzki et al. fabricated a fluorinated-based SIPE, in which homopolymers contained a polysulfonylimide segment in the polymer backbone. This polymer was obtained by combining carboxyl(benzene sulfonyl)imide with different dianiline units to form an amide linkage. The optimized result was obtained for a blend of this polymer with PVDF–HFP in the ratio 3:1 to obtain an ionic conductivity of 0.52 mS cm^−1^ at room temperature [328]. Note, however, that a liquid plasticizer is needed to reach this value; the EC:PC ratio of 1:1 (v/v) was selected owing to its high thermal stability. Owing to its–C(CF_3_)_2_ functional group, this polymer showed an enhanced solubility in THF, which favored the lithiation. In addition, as it is a single-ion polymer, its Li^+^ transference number was remarkably high, being 0.9. At 0.1C, the cell with this electrolyte, LiNi_1/3_Mn_1/3_CO_1/3_O_2_ cathode, and lithium-metal anode delivered capacities of 115 mAh g^−1^ after five cycles and 100 mAh g^−1^ after 100 cycles at room temperature. PVDF–HFP-based GPEs containing PC-based liquid electrolyte were developed successfully to enhance the safety performance of LiNi_0.5_Mn_0.3_Co_0.2_O_2_/graphite batteries [329].

Nitrile-based polymer electrolytes have attracted research interest because of their high dielectric constant, high anodic oxidization potential, and strong coordination ability [330]. Their properties for potential application in flexible, solid-state, or high-voltage lithium-ion batteries have been reviewed in reference [331]. However, the disadvantages of nitrile-based polymer electrolytes, such as a poor cathodic stability with poor compatibility between the polymer electrolyte and the lithium electrode or lithiated graphite, limit their further application.

**Figure 10:**
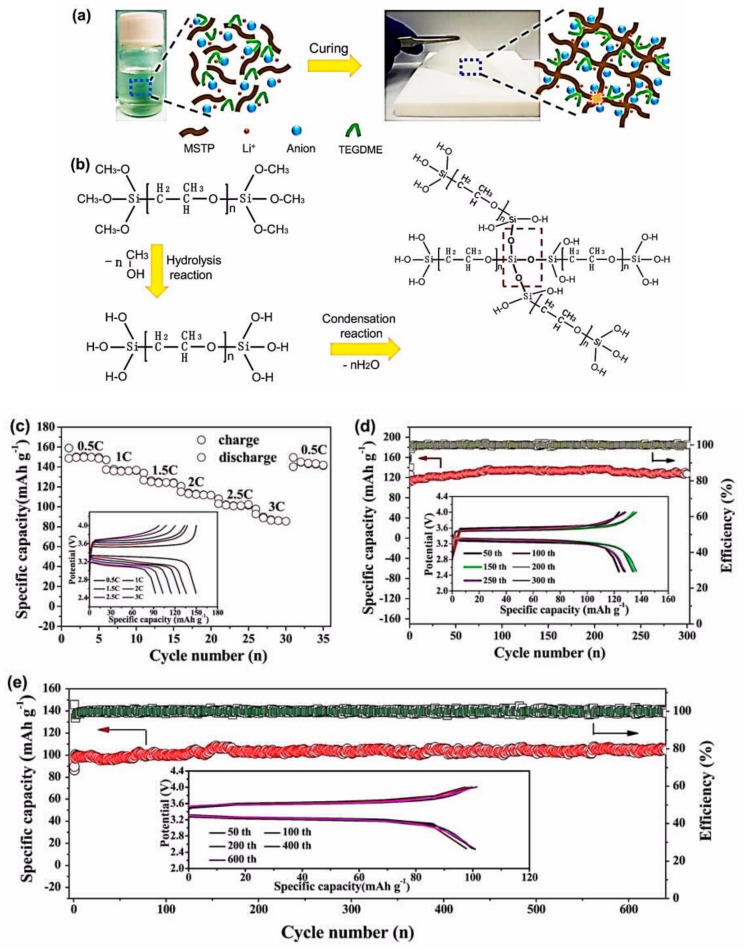
(**a**) Optical images of the modified silyl-terminated polyether (MSTP) polymer electrolyte (MSTP-PE) before and after polymerization, and schematic illustration of the preparation process with the structure of the cross-linked network of MSTP-PE. (**b**) The molecular structures of the MSTP monomer and MSTP-PE, along with the polymerization process. The chemical bonds in the light red area are the cross-linking parts. (**c**) Rate performances of LiFePO_4_/MSTP-PE/Li battery at room temperature. Cycling performance of LiFePO_4_/MSTP-PE/Li battery at (**d**) 1C and (**e**) 3C. The insets are the selected charge–discharge curves for the batteries with different cycles. Reproduced with permission from [332]. Copyright 2017 Elsevier.

A novel polymer electrolyte was obtained based on MSTP, which was synthesized via polymerization of the monomer with LiTFSI as the lithium salt and TEGDME as the plasticizer (Figure 10) [332]. The conductivity of this SPE was 3.6 × 10^−4^ s cm^−1^, but, most important of all, the transference number was high (0.65), which explained the remarkable electrochemical properties obtained for Li//LiFePO_4_ cell at room temperature: A capacity of 130 mAh g^−1^ at 1C rate over 300 cycles, and a stable capacity of 100 mAh g^−1^ at 3C over 600 cycles. This electrolyte exhibited a stability window that extended up to 5 V, but, to the best of our knowledge, has not yet been tested as an electrolyte with a cathode of the 4-5 V family.

Recently, Duan et al. extended the electrochemical window of solid electrolytes by employing a multilayered structure, with the aim being to use NCM622 and NCM811 cathodes [333]. An oxidation tolerant polymer, PAN, was in contact with the cathode, whereas a reduction tolerant polymer, PEGDA, was in contact with the lithium anode. In between, PAN/Li_1.4_Al_0.4_Ge_1.6_(PO_4_)_3_ (LAGP; 80 wt.%) composite electrolyte was employed, with the PAN-rich side contacting the PAN on the cathode for good interface compatibility, while LAGP contacted the PEGDA of the anode side to avoid dendritic penetration. The overall structure had a thickness of 25 μm. This heterogeneous multilayered solid electrolyte (HMSE) showed a conductivity of 3.7 × 10^−4^ S cm^−1^ and an electrochemical window that extended up to 5 V. The solid-state Li/HMSE/NCM622 cell delivered a capacity of 180 mAh g^−1^ at 0.5C, with a retention of 81.5% after 270 cycles. The discharge capacity of the Li/HMSE/NCM811 cell stabilized at approximately 170 mAh g^−1^ over 100 cycles, which corresponded to 97.7% of the initial capacity.

On the other hand, the PVDF-Li_10_SnP_2_S_12_ solid-state electrolyte with its electrochemical window extending up to 4.5 V and a transference number of 0.6 was tested in a Li//LiFe_0.2_Mn_0.8_PO_4_ cell [87]. The cell delivered a capacity of 130 mAh g^−1^ at 0.5C at room temperature, with the capacity retention being 88% after 140 cycles.

### 4.2. Extension to Five-Volt Class Cells

A higher voltage means a higher energy density. The decomposition of the conventional liquid electrolytes near 5 V, however, hinders the development of lithium-cells, which has been the motivation for the intense research on solid electrolytes, or, at least, GPEs that would be both stable at this high voltage and compatible with lithium. LiNi_0.5_Mn_1.5_O_4_ is the archetype positive electrode belonging to the 5 V class. As garnets are stable up to 6, they can be selected for this purpose. Dense composite electrodes can be prepared at room temperature by aerosol deposition of LiNi_0.5_Mn_1.5_O_4_ (LMN) and LATP to form LiNi_0.5_Mn_1.5_O_4_-LATP composite on a platinum sheet as a substrate. After annealing at 500 °C in dry air to improve the crystallinity of LMN, lithium phosphorus oxynitride (LIPON; ca. 5 μm thick) and lithium metal (ca. 3 μm thick) films were sequentially added by RF magnetron sputtering and vacuum evaporation, respectively, to obtain a solid-state cell. However, the cycle life was small, because the volume change of LiNi_0.5_Mn_1.5_O_4_ during cycling resulted in a loss of contact with LATP [334]. It should be mentioned that magnetron sputtering suffers from a very low deposition rate, therefore, it is an expensive and not well scalable process. In addition, LIPON displays a moderate ionic conductivity (10^−6^ S cm^−1^ at room temperature), and a lot of research is devoted to the synthesis of thinner films, as 5 μm appears to be too thick. LIPON can be grown by metal–organic CVD method [335]. Moreover, the films prepared by CVD avoid the formation of cracks [336]. The limitation of the cycling life to a few cycles, reported in reference [334], might be simply attributed to the way in which the LIPON film was synthesized.

Li_2_FeMn_3_O_8_ (LFMO) was selected by Han et al. as the cathode to demonstrate a high-voltage cell [131]. The cathode, comprising LFMO, carbon black, and PVDF (binder), was fabricated by conventional slurry-coating on an aluminum foil. A small amount of high-voltage liquid organic electrolyte was added in between the cathode and Li_7_La_2.75_Ca_0.25_Zr_1.75_Nb_0.25_O_12_ electrolyte to reduce the interfacial resistance, and an ultrathin Al_2_O_3_ layer deposited by ALD was used between the garnet and the lithium metal. The capacity at 0.1 C (1 C = 150 mA g^−1^) stabilized at approximately 110 mAh g^−1^ over 50 cycles.

Nonwoven PTFE is regarded as an ideal polymer matrix for developing advanced separators, but it is difficult for the electrolyte to penetrate the interspace of the nonwoven PTFE owing to its low surface tension. To overcome this difficulty, Chai et al. modified PTFE with poly(methylethyl a-cyanoacrylate) (PMCA) to synthesize a novel composite polymer that was impregnated with LiBOB salt. After drying, this composite membrane was saturated with PC containing 1 mol L^−1^ LiBOB to obtain a GPE for 5 V lithium batteries that was gifted with a conductivity of 1.24 × 10^−3^ S cm^−1^ and a transference number *t*_Li+_ = 0.63 [337]. The LiNi_0.5_Mn_1.5_O_4_//graphite full cells fabricated using this GPE delivered a capacity of 118 mAh g^−1^ at room temperature at 0.5C, with the capacity retention being 91.5% after 100 cycles. Note that the composition of the composite polymer was inspired by the poly(ethyl α-cyanoacrylate)-based GPE proposed by Hu et al. to improve the cycling performance of LiMn_2_O_4_-based batteries at high temperatures [338]. As PMCA reveals a better stability, it would be interesting to test it now in cells with LiMn_2_O_4_ cathodes.

We have already pointed to the interest in using PPC-based polymers that have been tested with the LFP positive electrode. Recently, a GPE obtained by blending PPC with PVDF–HFP as the polymer host has been used as the electrolyte in a lithium cell with LiFe_0.2_Mn_0.8_PO_4_ cathode in the potential range of 2.5 to 4.4 V [339]. The cell at room temperature delivered a capacity of 155 mAh g^−1^ at 0.2C, with a capacity retention of 89.8% after 100 cycles. The use of PPC is also promising in the context of the 5 V class. Zhao et al. have fabricated a cellulose-supported PPC membrane. Here, the robust cellulose helps overcome the poor mechanical integrity of PPC. This membrane was then submerged in the PC/LiDFOB liquid electrolyte to form a GPE that was stable up to 5 V, with an ionic conductivity of 1.14 × 10^−^^3^ S cm^−^^1^ at room temperature and an ion transference number of 0.68 [340]. The cycling performances of LMN//Li cell using this GPE were investigated between 3.5 and 5 V at room temperature. The cell delivered capacities of 109 mAh g^−^^1^ at 0.5C, of which 91.3% was retained after 100 cycles, and 80 mAh g^−^^1^ at 2C.

## 5. Li/Li-O_2_, Li-Air Batteries

Li-O_2_ cell displays a theoretical capacity of 3862 Ah kg^−1^, which corresponds to an energy density of 11.68 kWh kg^−1^ for a potential of approximately 3.0 V. The discharge of Li-O_2_ cell proceeds along the following steps [341,342,343,344,345,346]. First, oxygen is reduced to superoxide:

O_2_ + e^−^ → O_2_^−^ (1)

Li^+^ + O_2_^−^ → LiO_2_. (2)

Then, further reduction to lithium peroxide occurs through disproportionation of lithium superoxide:

LiO_2_ + LiO_2_ → Li_2_O_2_ + O_2_ (3)

or through an additional electrochemical reduction step:

LiO_2_ + Li^+^ + e^−^ → Li_2_O_2_. (4)

Upon charging, oxidation of Li_2_O_2_ yields Li^+^ and O_2_ gas [347].

According to these equations, Li-O_2_ batteries exhibit the potential to completely replace gasoline in vehicles on a weight basis. This has been the motivation for extended studies, and continuous progress has been made in the last 20 years. The advances and challenges can be found in several reports [348,349,350,351]. However, this battery still experiences critical issues that need to be addressed in order to make it commercially viable. In practice, the performance of Li-O_2_ batteries is far from expectation. The energy density remains well below the theoretical value, and the rate capability and cyclability are not yet enough to envision commercial use. Discharge intermediates involving peroxide anions, a powerful oxidant, and superperoxide, which is a powerful nucleophile anion, cause side reactions on the lithium anode. Even more dramatically, it has been shown recently that the extremely aggressive singlet oxygen O_2_ was formed during the disproportionation [346] These side reactions make it even more difficult to address the problem of dendrite formation and corrosion on the lithium anode [352,353]. It is then vital to control the discharge process of Li-O_2_ batteries, which requires efforts that are devoted to all parts of the cell, including the electrolyte, catalyst activity, and cathode structure [354]. The slow kinetics are attributed to the 2 mol e^−^/mol O_2_ peroxide chemistry [355]. Today, however, it is already possible to obtain a reversible 1 mol e^−^/mol O_2_ process with a cathode consisting of iridium nanoparticles on a reduced graphene oxide (rGO) electrode in a liquid electrolyte [356]. Indeed, many Li-O_2_ batteries utilize nonaqueous liquid electrolytes. However, their chemical and electrochemical instability, volatility, and flammability pose safety problems, as in any lithium battery, but, in the particular case of Li-O_2_ batteries, the liquid electrolytes are not stable in the harsh environment of the battery, which contains extremely reactive oxygen species. Therefore, the practical use of such liquid electrolytes is simply precluded by the oxygenated environment.

Furthermore, it is well known that the limited cyclability of Li-O_2_ cells with a carbon electrode is mainly due to the formation of Li_2_CO_3_-like species or other reaction byproducts on the cathode surface during the discharge and charge processes, with the decomposition of such species occurring at 4.2–4.5 V [357,358,359,360]. Li_2_CO_3_ originates from the CO_2_ in the air or electrolyte, or that produced by carbon oxidation. To inhibit these side reactions that are detrimental to the cycling life, the cycle performance is usually measured when the discharge capacity is limited to 500 mAh g^−1^ at different current densities. These will be the cycling conditions for the whole section devoted to Li-air or Li-O_2_ batteries, unless specified otherwise.

**Figure 11:**
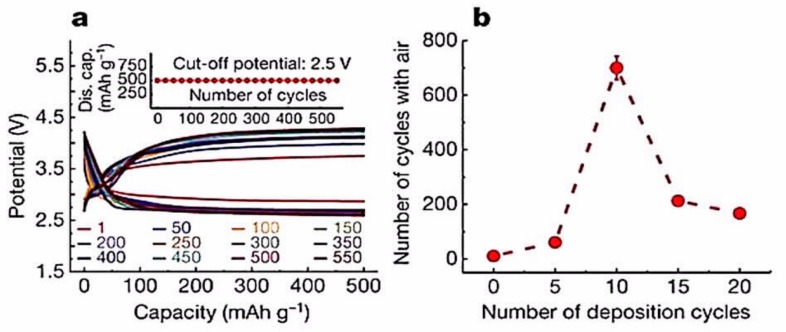
(**a**) Discharge–charge voltage profiles up to a capacity of 500 mAh g^−1^, with a constant current density of 500 mA g^−1^ of the Li-air cell with MoS_2_ cathode and an ionic liquid/DMSO EMIM-BF_4_/DMSO (25%/75%) electrolyte, over 550 cycles. The inset shows the capacity as a function of the number of cycles. (**b**) Dependence of the number of battery cycles in air on the number of deposition cycles used to form the anode-protection Li_2_CO_3_/C layer. Reproduced with permission from [362]. Copyright 2018 Nature.

Some works have pointed to the utilization of MoS_2_, either as a cathode [361] or as a catalyst [362], in Li-air batteries, but the breakthrough came recently for a system comprising a lithium carbonate-based protected anode, MoS_2_ cathode, and mixture of the ionic liquid 1-ethyl-3-methylimidazolium tetrafluoroborate (EMIm-BF4) and DMSO as the electrolyte (Figure 11). This system operates as a Li-air battery in a simulated air atmosphere with a long cycle life of up to 700 cycles at a constant current density of 500 mA g^−1^, based on the carbon weight (against 10 cycles, only in the absence of anodic protection, which is thus the critical parameter) [362]. This result is a promising step toward engineering the next generation of lithium batteries with much higher specific energies.

### 5.1. Solid Electrolytes

Solid electrolytes are desired in the design of flexible batteries [348,362,363]. The attempts to obtain Li-air or Li-O_2_ flexible batteries using liquid electrolytes have been rarely successful because of the leaking during twisting and bending [364,365]. There is, however, an exception. To fabricate a flexible rechargeable Li-air battery, Liu et al. employed lithium triflate (LiCF_3_SO_3_)–TEGDME, which is known for its relatively high stability toward superoxide (O_2_^−^) [366]. They developed a binder-free cathode that was formed by hierarchical rutile (TiO_2_) nanowire arrays (TiO_2_ Nas) that were uniformly grown on nonwoven carbon textiles, which were labeled as TiO_2_ Nas/CT. The Li-air battery with this cathode and electrolyte, a glass-fiber membrane, and a lithium-foil anode could reveal a stable cycling life of more than 350 cycles, with a capacity limit of 500 mAh g^−1^ at the current density of 100 mAh g^−1^ [367]. Severely bent and twisted cells were used to power a commercial red LED display screen without damage. Nevertheless, solid electrolytes are highly desirable for flexible batteries. The PEO/LiTFSI solid electrolyte can be used only at the high temperature of 80 °C [368], and other solutions are needed to obtain a cell that works at room temperature.

Liu et al. have fabricated an original woven-type battery consisting of a lithium-foil anode, a hydrophobic polymer electrolyte, and an air cathode [369]. The cathodes and anodes, being orthogonally double-woven, press against each other without the need for other components to provide pressure to ensure normal operation of the battery. As the air-diffusion layer and the packing material are the main contributors to the weight in commonly assembled metal–air batteries, the gain in energy density in this geometry, which is typically avoided, boosted the integral energy density to a record 523 Wh kg^−1^. In addition, this battery showed excellent mechanical suppleness.

A new polymer electrolyte based on lithiated perfluorinated sulfonic acid ionomer (PFSA-Li) was employed by Shi et al. to fill the pores of a PTFE substrate that was used to reinforce the soft polymer electrolyte [370]. A test cell was fabricated by employing the PFSA-Li/PTFE membrane swollen with DMSO solvent as both a polymer electrolyte and separator and multiwalled carbon nanotubes (MWCNTs) as the cathode. The discharge/charge curves obtained at a constant current density of 1.0 A g^−1^ and a limited capacity of 1000 mAh g^−1^ (0.5 mAh cm^−2^) showed a good cycling stability and stable terminal voltages over 90 cycles.

A novel Li-air battery-array pack consisted of arrays of small-scale air cathodes and lithium anodes that were interconnected by carbon ropes and copper wires [363]. The air cathodes and anodes were isolated by a poly(vinyl formal) (PVFM)-based Janus cross-linked membrane as the GPE supporter, which consisted of a dense side and the other porous side with the MWCNT coating. The discharge–charge plateaus of this pack barely changed even after 10,000 cycles of folding/stretching, and the pack revealed a high gravimetric energy density of 295 Wh kg^−1^ and a volumetric energy density of 274 Wh L^−1^.

### 5.2. GPEs

Some polymers cannot be used with Li-air or Li-O_2_ batteries because of their oxidation and instability in the presence of Li_2_O_2_, which is due to the existence of oxygenated radicals that are produced during the discharge process. Such polymers include PAN, PVC, PVDF, and poly(vinylpyrrolidone) (PVP) [371,372,373]. PEO cannot be used either, because of the formation of formate-polymeric species from the degradation of polymer electrolyte, and their irreversible deposition is detrimental to the cycling life [374]. Another reason is that PEO auto-oxidizes in an oxygenated environment [375]. Poly(3,4-ethylenedioxythiophene) (PEDOT) is also prohibited, because the formation of sulfone functionalities on the PEDOT surface and cleavage of the polymer repeating unit impairs the electronic conductivity and leads to poor cycling life [376].

The case of the PVDF–HFP-based GPE is under debate. Such a GPE is popular in lithium batteries, especially when it is coupled with LiTFSI, because the interaction between the Li^+^ in LiTFSI electrolyte and the fluorine atoms in the macromolecular chains of PVDF–HFP is beneficial to the diffusion of Li^+^ [377]. A blend of cellulose acetate (CA) and PVDF–HFP, synthesized using the solution casting technique, which was followed by impregnation with LiTFSI solution, was tested as an electrolyte between the Super-P black carbon cathode and the lithium anode of a Li−O_2_ cell [378]. A good capacity retention was obtained, but the testing was conducted over 40 cycles only. At the least, the electrochemical stability improved with respect to the standard polyethylene (PE) separator plus liquid electrolyte configuration. On the other hand, Jung et al. incorporated Pyr14 TFSI in a PVDF–HFP-based GPE [379] and observed that PVDF–HFP undergoes extensive elimination reactions upon exposure to peroxide, which was also confirmed by Amanchukwu et al. [371]. However, with a PVDF HFP-based membrane, few signs of deterioration were detected over 16 cycles in a Li-air cell [380]. Some researchers claim the merits of PVDF–HFP, considering that its use is promising [381]. However, the cycle life span of the cell with GPE was not satisfactory: The ethoxylated trimethylolpropane triacrylate (ETPTA)/PVDF–HFP/liquid electrolyte they proposed was stable for less than 100 cycles [382], and the free-standing GPEs with PVDF–HFP matrix plasticized with TEGDME was tested over 50 cycles only [383], whereas PVDF–HFP with l-ethyl-3-methylimidazolium bis-(trifluoromethanesulfonyl)-imide (EMI-TFSI) was not even stable over 20 cycles [384]. Note also that EMI-TFSI-LiTFSI was found to be inappropriate for Li-air batteries; therefore, this result is not necessarily attributable to PVDF–HFP [385]. We also note that PVDF–HFP was used recently to apply the coaxial-type design to the Li-air battery. A cable-type Li-air battery containing a lithium wire anode, a PVDF-HFP-based polymer electrolyte prepared with 2-hydroxy-2-methyl-1-phenyl-1-propanone (HMPP) UV initiator, and an aligned-carbon-nanotube cathode could be deformed without damage through a dynamic bending process at a speed of 10 degrees per second [386]. The electrolyte was actually a GPE, formed by the addition of LiTF-TEGDME. This cell was able to cycle at the current density of 1400 mA g^−1^ at a capacity that was fixed at 500 mAh g^−1^, with the voltage decreasing linearly from 2.5 V with the increase in cycle number, though it was still maintained at 2.2 V after 100 cycles. A battery pack of three cells connected in parallel and woven into a flexible powering textile delivered a discharge voltage of 8 V and could power commercial LED equipment successfully, even under water. In addition, a flexible and stretchable Li-air battery has been developed by designing a rippled air electrode made of aligned carbon nanotube sheets, a lithium array electrode, and a GPE based on PVDF–HFP [387]. This battery supported 180 cycles at a capacity that was limited to 500 mAh g^−1^ and at a current density of 1000 mAh g^−1^. Moreover, the discharge voltage was only moderately affected by 1000 stretching cycles, which were carried out at a strain of 75%; the bending cycles, carried out at a bending angle of 90°; and the twisting cycles, performed at a twisting angle of 180°, which proved its ability to be used in wearable electronics. To the best of our knowledge, these are the best results obtained with PVDF–HFP for a Li-air battery. Another result that does not favor the use of PVDF in Li-O_2_ batteries is the fact that PVDF/p-benzoquinone (pBQ) GPE with LiTFSI/TEGDME could be cycled for only 30 cycles [388]. On the other hand, the combination of PAN and tetrachloro-1,4-benzoquinone (tCl-pBQ) with LiTFSI/TEGDME yielded much better results, with the stability increasing to 89 cycles [389]. Note, however, that the difficulty with PVDF–HFP is specific to Li-O_2_ or Li-air batteries, which originates from the fact as mentioned above that O^2−^ and Li_2_O_2_^2−^ are very strong nucleophiles and bases, respectively, that can react with the GPE.

A GPE consisting of a polymer and an ionic liquid, with or without salts, can control the oxygen reduction chemistry in a Li-O_2_ cell, support the formation of ionic liquid-superoxide complexes, and reduce the number of reactive species present in the cell [355]. PMMA was selected as the GPE because of its stability in contact with Li_2_O_2_ at room temperature, with or without LiTFSI salt [371]. In the absence of Li^+^ (a film without LiTFSI), the ionic liquid cation (either Pyr^+^, EMI^+^, or BTM^+^) can act as a Li^+^ substitute and is capable of complexing the superoxide oxygen reduction products, which explains why discharging can occur at 1.96 and 2.1 V for Pyr14TFSI- and EMI-TFSI-based cells, respectively [390]. This complexation of the superoxide with the Il cation is a one-electron process. Note that this complexation is observed only in the GPE-based Li−O_2_ cells because PMMA acts as a diffusion barrier that actively limits the transport of Li^+^ from the oxidized anode. On the other hand, this is not observed in pure ionic liquid Li−O_2_ cells because lithium ions can easily migrate from the anode. Now, if the lithium salt LiTFSI is added to the ionic liquid, then the oxygen reduction process does not stop at the superoxide, but continues to yield peroxide, which corresponds to a two-electron process [371]. However, the kinetics associated with the two-electron process are sluggish, as revealed by the 2 mol e^−^/mol O_2_ process of forming Li_2_O_2_ that occurs when ionic liquid/salt systems based on 1-butyl-1-methylpyrrolidinium TFSI and EMI-TFSI are used as the electrolyte [391]. Moreover, the conductivity of the electrolyte is decreased when the ratio Li^+^/Pyr^+^ increases, due to the interactions between the PMMA carbonyl groups and the lithium ions that limit ion transport. Therefore, the main result obtained from reference [371] is the ability to realize 1 mol e^−^/O_2_ chemistry through a GPE, without the addition of expensive catalysts.

A 100 cycles at the current density of 200 mA g^−1^ were obtained with polypropylene (PP)-supported (PMMA)−blend−poly(styrene) doped with nanofumed SiO_2_ as the electrolyte and Super-P carbon cathode [392]. This is an improvement with respect to the stability of 40 cycles obtained in reference [378] for the PVDF–HFP-based GPE, which presents another evidence of the limits of PVDF in Li-O_2_ batteries. The other results obtained with PVDF–HFP will be given below in the section emphasizing the role of SiO_2_ filler.

Meng et al. proposed a PVFM-based Janus membrane consisting of one dense side to prevent the formation of lithium dendrites, with the other side prepared by coating with MWCNTs, which assist the cathode in forming an enlarged electrolyte-wetted interface [393]. This membrane punched into a disk 16 mm in diameter was impregnated with 200 μL of a liquid electrolyte composed of 1.1 mol L^−1^ LiTFSI in the mixed solvents of DMSO and TEGDME (in a volume ratio of 8:2). The corresponding Li-O_2_ battery with δ-MnO_2_ @CNTs as the cathode survived 150 cycles at 1000 mAh g^−1^ capacity limit at a current density of 200 mA g^−1^, with a narrow voltage gap of 0.90 V. This extended life, however, is partly attributed to the better catalytic property of MnO_2_ than that of MWCNTs [394]. These results also illustrate the good association of TEGDME and LiTFSI in Li-O_2_/air batteries. In glyme-based electrolytes containing LiTFSI salt, the salt decreases the ionic association [395], which, in turn, increases the Li^+^ conductivity.

### 5.3. Addition of Ceramics

The introduction of a ceramic film has been considered as a solution to avoid side reactions on the lithium anode [396]. Ceramics combine strong mechanical stiffness with high lithium transference numbers but must be combined with polymers to avoid large solid–solid interfacial resistances and fast formation of dendrites on the anode. Such solid electrolytes, including polymer and ceramic electrolytes, are known to be competitive alternatives to liquid electrolytes in Li-O_2_ batteries and improve battery safety [397,398,399]. Several reasons can be provided for this observation. First, the anions of the lithium salt absorb strongly on the Lewis acid groups on the surface of the ceramic fillers in composite polymer electrolytes, which enhances dissociation [400]. Secondly, they can improve the performance of Li-O_2_ batteries by stabilizing the interfacial resistance and preventing lithium anode corrosion.

We have already mentioned that LAGP ceramic affords good results in Li//LiFePO_4_ batteries. In Li-O_2_ batteries, this ceramic is even more interesting because it can permit adsorption of oxygen molecules on its surface, which is followed by the reduction of oxygen and the formation of Li_2_O_2_. An HSE integrating poly(methyl methacrylate-co-styrene) (PMS) and LAGP (1:1 w/w) was fabricated and reported in reference [401]. After mixing the PMS with LAGP in THF, the HSE was prepared through the phase inversion process and reinforced by a PE support to obtain a film of thickness 30 μm, with an ionic conductivity of 0.32 × 10^−^^3^ S cm^−^^1^, a transference number *t*_Li+_ = 0.75, and a stable window of up to 5.2 V vs. Li^+^/Li. This HSE was tested as an electrolyte with a cathode prepared by gathering together silky single-layer graphene to form a cross-linked gel after entrapping sufficient ionic liquid 1-ethyl-3-methylimidazolium ([C_2_C_1_im]) LiTFSI [402]. The Li-O_2_ cell revealed a stability of 350 cycles at a current density of 200 mA g^−^^1^, with a capacity limit of 1000 mAh g^−1^ and negligible decay at 50 °C. Although LAGP is compatible with lithium metal, Zhou et al. coated a Ge^0^ film on LAGP by sputtering to eliminate the possible reduction of Ge^4+^ and improve the contact between LAGP and the lithium anode. The quasi-solid Li-air battery with this additional film operated for 30 cycles at the current density of 200 mA g^−1^ and a discharge capacity of 1000 mAh g^−1^ [403]. Germanium is only one of the lithiophilic materials, which also include silicon, ZnO, and Al_2_O_3_, which are used as alternatives to polymers such as PVDF–HFP and PEO by inserting at the interface between solid-state electrolytes and electrodes to decrease the interfacial resistance [126,145,289,404,405,406].

LATP was also integrated into a Li-air/O_2_ battery that delivered a capacity of 16800 mAh g^−1^ at 0.1 mA cm^−2^ by using the same strategy of wetting based on an integrated structure of electrode and electrolyte as in other lithium battery chemistries [407]. It consisted of coating an LATP membrane onto the surface of a pre-sintered air cathode that contained LATP powder plus 75% porous carbon. This wet-process, commonly used in lithium-ion batteries, has been newly applied in Li-air/O_2_ chemistry, and is a promising process for improving the performance of Li-air/O_2_ batteries [408].

Another ceramic aluminum-doped LLZO has also been used recently in a Li-O_2_ cell, in combination with a GPE inserted between the ceramic and lithium metal, to prevent direct contact between LLZO and lithium and, also, to obtain a good interfacial contact. An integrated cathode was utilized in which a porous aluminum-doped LLZO solid electrolyte frame was covered with a carbon layer and CoO nanoparticles as the catalyst. The GPE was obtained by soaking an ionic liquid prepared by dissolving LiTFSI in PYR14TFSI (molar ratio of 1:9) in a SPE (PVDF–HFP containing 10 wt.% modified mesoporous silica filler) [409]. This cell was highly stable up to 100 °C and exhibited a long cycle life of up to 132 cycles in the limited capacity mode of 500 mAh per gram of C + CoO at 0.3 mA cm^−2^. This result also demonstrates a recent trend in combining the fluidity of room temperature ionic liquids with the high mechanical strength of solid matrices to form the so-called ionogels [410,411]. This combination is promising for all kinds of lithium-ion batteries, including Li-O_2_, where it can also protect the lithium anode against reactions with O_2_ species [355].

A composite GPE was obtained in the form of a flexible film by using ETPTA (*M*_w_ = 428 g mol^−1^), and HMPP as the photoinitiator in the ratio of 1:99 by weight (HMPP:ETPTA) [412]. The liquid electrolyte consisted of TEGDME solvent, in which LiTFSI was dissolved. The GPE was prepared by mixing HMPP:ETPTA monomer into the liquid electrolyte (80:20 wt.% EPTA:liquid electrolyte), along with the addition of 1 wt.% glass microfillers. The introduction of the ceramic glass was beneficial to the cycling life of the Li-O_2_ cell, which, however, did not exceed 54 cycles. The authors attributed this limitation to the formation of lithium carbonate on the cathode, due to the degradation of the tetraglyme-based solvent in the electrolyte. However, as already mentioned in this review, the formation of lithium carbonate is often observed on carbon electrodes without the presence of glymes in the electrolytes, and the much longer cycling life observed in Li-O_2_ batteries with the combination TEGDME/LiTFSI proves that the problem more probably arises from the use of PVDF as the cathode, which was prepared by coating CNTs/PVDF slurry on a carbon cloth gas diffusion layer in the absence of a catalyst.

A hybrid quasi-solid-state electrolyte (HQSSE) that combines poly(methyl methacrylate-styrene) with amorphous LiNbO_3_ in THF was obtained by the phase inversion process by using PE as a support, with SiO_2_ as the nanofiller [413]. LiNbO_3_ was preferred to LATP owing to its larger lithium diffusivity. To avoid clogging of the interface between HQSSE and single-walled carbon nanotubes (SWCNTs) due to the formation of Li_2_CO_3_-like species that hinders the diffusion of O_2_ and Li^+^, lithium-salt-modified SWCNTs (LSM) and an ionic-liquid-based cross-linked network gel (CNG) were used as the cathode. As a result, the terminal voltages of the discharge process for the solid-state Li-O_2_ battery were almost unchanged over 100 cycles at the current density of 250 mA g^−1^, and stable discharge processes were obtained even at the high current density of 2000 mA g^−1^_._ This remarkable performance at high rates is attributed to the combination of a high conductivity of the HQSSE (0.2 mS cm^−1^ at room temperature) and the LSM@CNG cathode, which provides efficient pathways for electrons, ions, and oxygen [402,414,415].

### 5.4. SiO_2,_ an Important Filler

Not only is SiO_2_ a plasticizer, but it also promotes the dissociation of the electrolyte and increases the ionic conductivity. PEGMA can be used as an ion-conducting agent in Li-O_2_ batteries. By coupling with methacrylated tannic acid (MTA), which acts as a cross-linker, and nanofumed silica, which is a filler, a polymer composite electrolyte was obtained [416]. This composite achieved a remarkable ionic conductivity of 0.14 × 10^−3^ S cm^−1^ at room temperature, which was not only attributed to the SiO_2_, but also because of the small amount of MTA, which allowed polymerization and cross-linking of PEO derivatives into free-standing films despite the short chain lengths of EO. For the evaluation of the electrochemical properties, infiltration of 10 μL of a liquid electrolyte (1 mol L^−1^ LiCF_3_SO_3_ in TEGDME) was carried out to improve the contact between the lithium powder anode and the polymer electrolyte. SiO_2_ was employed here to reduce the crystallinity of the solid electrolyte and increase the dissociation of the lithium salt, which further increases the ionic conductivity. Using a Pd_3_Co/MWCNT cathode, a lifetime of 125 cycles at the current density of 100 mA g^−1^_MWCNT_ was obtained, when the discharge capacity was limited to 500 mAh g^−1^_MWCNT_. Part of the result may be attributed to the choice of the cathode, as Pd_3_Co displays an excellent catalytic activity [417], but it provides evidence that this quasi solid-state electrolyte is a step toward the development of Li-O_2_ batteries. It also illustrates the interest in the introduction of SiO_2_ into the polymer matrix of electrolytes, which is also known to improve their resistances to flames and high temperatures. These positive effects of SiO_2_ were also observed in Li- and Na-CO_2_ batteries [418,419].

**Figure 12:**
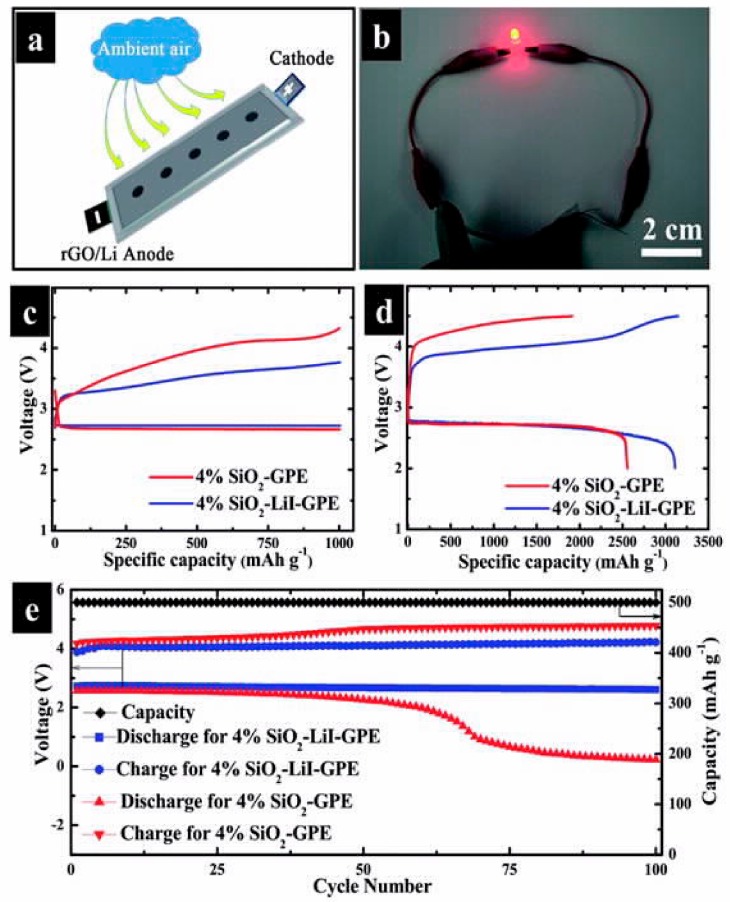
(**a**) Schematic of the operation of a flexible belt-shaped Li-air battery using 4% SiO_2_–LiI GPE with rGO/Li anode in ambient air; (**b**) photographic profile of a LED driven by this cell under bending conditions; discharge and charge curves obtained at a current density of 100 mA g^−1^ (**c**) with a fixed capacity of 1000 mAh g^−1^ and (**d**) under full discharge/charge conditions; (**e**) voltage at the discharge/charge terminal at a current density of 100 mA g^−1^ and a fixed capacity of 500 mAh g^−1^ as a function of cycle number. Reproduced with permission from [419]. Copyright 2018 Royal Society of Chemistry.

Another SiO_2_-based GPE was obtained by mixing 4 g of solution A (1 mol L^−1^ LiTFSI and 0.05 mol L^−1^ LiI in TEGDME), 5 g of solution B (1 g PVDF–HFP/4 g NMP), and 3.01 g of solution C (0.01 g HMPP in 3 g TMPET), where TMPET is trimethylolpropane ethoxylate triacrylate, to which was added *x* wt.% SiO_2_ before UV irradiation [420]. The best results were obtained for *x* = 4, and a Li-air cell using this electrolyte with a rGO/Li anode and a rGO-based cathode showed a lifetime of 100 cycles at a current density of 100 mA g^−1^ and a fixed capacity of 500 mAh g^−1^ (Figure 12). Note that this cell was actually a Li-air cell, and not a Li-O_2_ cell. Indeed, many conventional polymer electrolytes cannot prevent the potential contamination of the lithium anode by air [421,422,423,424], in which case Li-air cells suffer from significant voltage polarization, and only Li-O_2_ cells can be built. Safe anodes for Li-air batteries then require lithium-alloying materials, and the results of reference [417] show that rGO/Li is one of them, which led the authors to conclude that combining an rGO/Li anode with a compact GPE containing SiO_2_ may be one of the ultimate choices for flexible Li–air batteries that can operate in ambient air. In addition, this GPE is not flammable. Another GPE also utilized SiO_2_ as a filler, PVDF–HFP as a polymer matrix, and liquid LiTFSI as a plasticizer [425], like what was reported in reference [420]. However, the optimized amount of SiO_2_ was 20 wt.% (against 4 wt.% in reference [420]), in which case the conductivity reached 0.93 mS cm^−1^ at room temperature. A comparison between the electrochemical properties of a Li–O_2_ cell prepared with this electrolyte and a cell prepared with liquid electrolyte showed that the results obtained by cycling them at different current densities with the capacity limited to 6000 mAh g^−1^ are comparable. The only significant difference was in the cycling stability, which was 89 cycles (890 h) for the cell with the GPE and only 50 cycles (500 h) for the cell with the liquid electrolyte. A PVDF–HFP polymer including aluminum-doped LLTO covered with a modified SiO_2_ layer was fabricated by Le et al. After activation in a solution of 1 mol L^−1^ LiTFSI in TEGDME, this GPE was placed between the lithium anode and the glass fiber separator, which significantly improved the cyclability of the Li–O_2_ cell [317]. This cell operated up to 71 cycles under a limited capacity of 1000 mAh g^−1^, against only 47 cycles in the absence of this composite GPE.

A polyurethane membrane efficiently protects the lithium metal from the crossover of water and oxygen from the air-cathode side [426]. By utilizing the hydrogen bond between the thermoplastic polyurethane (TPU) and aerogel SiO_2_ in GPEs, Zou et al. developed a nonwoven fabric-supported aerogel SiO_2_-filled TPU matrix with a tetraglyme liquid component. The flexible Li-O_2_/air battery built with this electrolyte survived 250 cycles at the current density of 500 mA g^−1^ and a fixed capacity of 1000 mAh g^−1^ at room temperature [427]. It also worked in humid air (it powered a LED lamp for 20 days) and could be operated for more than 145 cycles (580 h) at 50 °C. Another choice of the GPE for Li-air batteries is possible [428], but it presented serious safety problems as it was easily combustible and decomposed at high temperatures.

It should also be noted that the morphology of the filler plays an important role, which we already pointed out in the previous sections on other battery chemistries. Chamaani et al. investigated the influence of 1D glass microfillers in GPEs using ethoxylated trimethylolpropane triacrylate polymer and tetraglyme-based solvent [429]. The introduction of 1 wt.% of such a glass microfiller in the GPE led to 37% improvement in the ionic conductivity and 25% increase in the transference number. The corresponding Li–O_2_ cell showed the highest discharge cycling performance, with a median of 54 consecutive discharge cycles, against 40 cycles for the GPE without the glass filler. We also note that all the polymers that we have mentioned were used as a component of the electrolyte for Li-air batteries and revealed a cyclability that needed to be improved; the results were actually disappointing, compared with their performances in other lithium-battery chemistries. The main reason is that the hydrogen atoms adjacent to the electron-withdrawing groups in polymers are vulnerable to attack by O_2_ reactive species. It is therefore desirable to replace some of the labile hydrogen atoms of the polymer chains with other functional groups to reduce the number of pathways for nucleophilic/oxidative attack [430]. Antioxidants such as phenol or amine stabilizers effectively reduce the deterioration of polymers [431].

### 5.5. Redox Mediators

One major challenge in Li–O_2_ or Li–air batteries is that re-oxidation of the insulating discharge product Li_2_O_2_ is quite difficult. Furthermore, the crystals of Li_2_O_2_ necessary for electrochemical regeneration can be nucleated away from the carbon grains/nanotubes. To avoid this problem, a redox mediator (RM) can be used in GPEs [388,428,432,433]. During charging, the RM first undergoes electrochemical oxidation into RM_ox_ at the cathode side. Then, RM^+^ converts back into RM by chemically oxidizing Li_2_O_2_ [434]. An example of RM is LiI, and a Li–air battery using GPE with 0.05 mol L^−1^ LiI as the electrolyte could stably cycle 400 times in ambient air (relative humidity of 15%), owing to the conversion of I^−^/I_3_^−^ [425]. Note, however, that Kwak et al. discovered that LiI is involved in side reactions of ether-based electrolyte solutions in Li-O_2_ cells [435], and recommended paying more attention to organometallic RMs [436]. A sandwich-structured quasi-SPE has been designed with separated catholyte and anolyte that is composed of PPC/Li-Nafion/PMMA SPE and TEMPO, which is a nitroxide radical precursor of the RM additive [432]. PMMA contained a small amount of TEGDME, and PPC was involved to obtain a favorable lithium anode/electrolyte interface, which further blocked the shuttling of the mediator. PMMA can absorb TEGDME (with TEMPO) to form a gel-like structure, and the amount of solvent was reduced by 90% with respect to that in a cell with liquid electrolyte. Owing to the permselectivity of Li-Nafion membrane in blocking the mediator shuttling to lithium metal, the Li–O_2_ cell using this quasi-SPE could still work for 200 cycles (at a fixed capacity of 500 mAh g^−1^ and a current density of 100 mA g^−1^), with a charge profile under 4 V. Heme biomolecule was used as a RM in a liquid electrolyte (TEGDME+LiClO_4_) [437], but, to the best of our knowledge, it has not been tested in GPEs.

A redox polymer was obtained by the incorporation of redox-active counter-anions based on anthraquinone and nitroxide groups into poly(DADMA) type PIL [438]; however, the cycle life with this electrolyte has not been explored yet. The migration of the RM to the anodic side may deteriorate the lithium-anode; therefore, the anode must be protected against this shuttle effect. The remedies that have been envisioned for the Li-O_2_ battery are the same as those used to avoid the shuttle effect of polysulfides in Li-S batteries, which are reviewed in reference [43]: Use a modified separator [439] or cathode [440], or add a protective layer such as a solid electrolyte [441,442]. Zhang et al. used InI_3_ as a RM that could generate a stable indium layer by reaction with lithium to prevent further consumption [443]. Using a sandwich-structured PPC/Li-Nafion/PMMA SPE and TEMPO as a cathodic additive, Liu et al. built a Li-O_2_ battery that remained stable over the 50 cycles that it was tested, because the migration of the RM was blocked by the Li-Nafion membrane [432]. A stable RM-decorated GPE composed of soluble LiI-decorated PP-supported PMS with nano-TiO_2_ doping was combined with (RuO_2_@RGO)-based cathode [444]. Owing to this interfacial engineering, the overpotential of the Li//O_2_ battery decreased markedly, with the terminal voltage at the end of charging being lower than 4 V, and it survived 50 cycles at the current density of 200 mA g^−1^ at a fixed capacity of 1000 mAh g^−1^. Note that the cycling stability of the cell is limited by that of the lithium metal anode, because the shuttling of I_3_^−^ to the anode is inevitable, which results in reduction of the oxidized RM and corrosion of the lithium anode.

### 5.6. Lithium Salts

Lithium salts are pivotal components of the electrolytes. We have recently devoted a review to the salts used in lithium, sodium, potassium, and magnesium batteries [445], in which, however, the attention was not focused on the Li–O_2_ chemistry. That is why this section is devoted to them, while we simply guide the reader to the review [445] for the other chemistries. This section is justified because most of the traditional inorganic salts used in lithium-ion batteries turned out to be unstable against Li_2_O_2_ and cannot be used in Li//O_2_ batteries [446]. Organic salts with sulfonate anions, in particular, LiTf, LiC_2_F_5_SO_3_, and LiC_4_F_9_SO_3_, are a better choice [447]. The problem with them is their low ionic conductivity, which is the reason that an imide salt, LiTFSI, is now preferred. With larger anions, so as to obtain a greater dissociation constant, LiTFSI displays a much higher conductivity. Studies on LIBs have shown that LiTFSI corrodes the aluminum of the current collector [448], even though other sources of corrosion have been invoked [449]. Furthermore, aluminum is usually not used as a current collector in Li//O_2_ cells, rather carbon felts are used. Large salt concentrations improve the performance of the cell [450,451]. A large salt concentration is also known to improve the transference number [452]. However, the application of concentrated electrolytes is restricted to GPEs (and of course liquid electrolytes). In SPEs, high salt concentrations deteriorate the mechanical properties, therefore, a compromise between the salt concentration and the mechanical properties have to be found.

## 6. Li–S Cells

The practical application of Li–S batteries is limited by their poor cyclability, which can be caused by the “shuttle effect” arising from the lithium polysulfides generated during the conversion of sulfur to the soluble Li_2_S_n_ (4 ≤ *n* ≤ 8), although some solutions have now been found to this problem (see reference [43] for a review). The stability of the lithium metal anode is also a big challenge. Nevertheless, Eshetu et al. determined that the use of lithium azide (LiN_3_) as a novel electrolyte additive in all-solid-state Li-S batteries solves this problem. It results in a compact and highly conductive Li_3_N passivation layer on the lithium anode that exhibits two beneficial effects, namely reduction of the shuttle effect and suppression of dendrite formation [453]. In commercial Li–S batteries, the current electrolyte is a liquid (with 1,3-dioxolane (DOL) and dimethoxyethane (DME) as the solvent). However, this electrolyte poses safety issues owing to its volatility and flammability, and a solid electrolyte is clearly needed to reduce the risk of thermal runaway [454,455]. The first improvement has been to combine this liquid electrolyte with a solid electrolyte. The solid electrolyte is used to oppose the shuttle effect, whereas the liquid electrolyte is utilized to maintain the ionic contact between the electrolyte and the electrodes. The best such hybrid electrolyte has been obtained through the choice of NASICON-type Li_1+x_Y_x_Zr_2−x_(PO_4_)_3_ (LYZP) (*x* = 0–0.15) as the solid electrolyte [456]. The Li_2_S_6_//Li cell with this hybrid electrolyte delivered a capacity of ≈1000 mAh g^−1^ (based as usual on the active sulfur material), with a capacity retention of ≈90% after 150 cycles. However, the dissolution of polysulfides in a liquid electrolyte is inevitable. The use of solid electrolytes is thus required to improve the cyclability, although the sulfur/Li_2_S loading achieved with solid electrolytes is still too low for practical application [457]. Actually, even the *x* = 0 case of LYZP, i.e., LiZr_2_(PO_4_)_3_, can be used as a solid electrolyte, because it reacts with a metallic lithium anode to form a Li^+^-conducting passivation layer containing Li_3_P and Li_8_ZrO_6_ that is wetted by the lithium anode and which also wets the LiZr_2_(PO_4_)_3_ electrolyte [458]. However, this electrolyte has been tested successfully only in lithium-ion batteries. The Li/LiZr_2_(PO_4_)_3_/LiFePO_4_ battery at 80 °C delivered capacities of 140 and 120 mAh g^−1^ with cell polarizations of 0.1 and 0.2 V at 50 and 100 µA cm^−2^, respectively, with a Coulombic efficiency of 99.5 ± 0.5% over 40 cycles. However, it has not been tested in Li–S cells; therefore, the compatibility with sulfur remains to be investigated, though it is likely good.

### 6.1. Use of Solid-State Electrolytes in Li–S Batteries

Garnets are used in Li–S batteries because their ionic conductivity is higher than that of NASICON electrolytes, but the interface formed with electrodes is more resistive. The same LLZT-2LiF solid electrolyte as that used in a cell with LiFePO_4_ cathode [149] was also employed in a Li–S cell in the same work. The polysulfide shuttling was effectively suppressed by the solid electrolyte. The reversible capacity stabilized at 988 mAh g^−1^ after 100 cycles, with a retention of 93% of the stabilized capacity at the second cycle at 200 μA cm^−2^. LLZO codoped by Al^3+^ and Nb^5+^ to yield a molar ratio of Al:Nb:La:Zr of 0.24:0.25:3:1.75 was used to fabricate a cathode by loading 61–64 wt.% active sulfur into a porous nanoparticle-decorated carbon foam, and an LLZO–PEO–LiClO_4_ electrolyte was directly coated onto the cathode [459]. The corresponding Li-S cell delivered 900 mAh g_sulfur_^−1^ at 37 °C for a current density of 0.05 mA cm^−2^ and up to 1556 mAh g_sulfur_^−1^ at 70 °C. At 37 °C, the capacity decreased to 800 mAh g_sulfur_^−1^ during the first 10 cycles, but then, the capacity retention was 98.7% after 90 cycles, which confirmed the good performance of PEO-based composite electrolytes in Li-S batteries [460]. However, a recent in situ investigation of the polysulfide shuttling in a Li–S battery with the ceramic-polymer LLZO–PEO composite electrolyte has shown polysulfide dissolution into the electrolyte during the discharging process, and the dissolved polysulfides still remain in the electrolyte during the subsequent charging [461]. This result is consistent with the irreversible loss in capacity observed during the initial cycles.

The solution proposed by Huang et al. for maintaining contact at the interface and increasing the cycle life was to add a second phase; they prepared solid Li_6.4_La_3_Zr_1.4_Ta_0.6_O_12_ (LLZTO) with 5%–9% nano MgO powders by a simple solid-state process [462]. Owing to the tantalum doping, the conductivity was maintained at 5 × 10^−4^ S cm^−1^ at room temperature and, owing to the second-phase MgO, the fracture strength was increased by 50% to reach 135 MPa. A battery cell consisting of Li/composite ceramics/sulfur-carbon at 25 °C exhibited a capacity of 685 mAh g^−1^ at 0.2C at the 200^th^ cycle, while maintaining a Coulombic efficiency of 100%.

**Figure 13:**
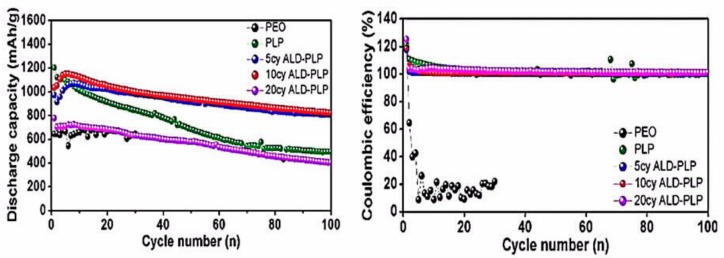
Li–S batteries employing PEO/LATP/PEO sandwich electrolyte with Al_2_O_3_-coated aluminum-doped LATP. Left: Cycling performances for different concentrations of LATP. Right: The corresponding Coulombic efficiencies. All cycling tests were performed at a current density of 0.1C (1C = 1670 mAh g^−1^) and 60 °C. Reproduced with permission from [463]. Copyright 2018 Royal Society of Chemistry.

Like in the lithium-ion batteries reviewed in the previous sections, polymer–ceramic sandwich electrolytes have been considered for Li–S batteries, with the choice of LATP as the ceramic. However, the new problem with respect to the lithium–sulfur chemistry in the earlier lithium-ion batteries is the deterioration of LATP by the polysulfides, which act as reductants of Ti^4+^ [463]. Therefore, LATP must be protected. Liang et al. have realized a protective coating on LATP by ALD of Al_2_O_3_ on the surface [464]. The PEO/LATP/PEO sandwich electrolyte with this Al_2_O_3_-coated aluminum-doped LATP could endow an all-solid-state Li-S cell with a discharge capacity of 823 mAh g^−1^ after 100 charge/discharge cycles at 0.1C at 60 °C (Figure 13).

Han et al. fabricated a Li_2_S–Li_6_PS_5_Cl–C composite cathode with a weight content of the active material Li_2_S of 59.6%. This composite cathode was obtained by dissolving Li_2_S, as the active material; PVP, as the carbon precursor; and Li_6_PS_5_Cl in ethanol, which was followed by a coprecipitation and high-temperature carbonization process [465]. The nanoparticles of Li_2_S and Li_6_PS_5_Cl were coated with PVP during the evaporation of ethanol at 100 °C, and the particles were coated with carbon during the carbonization of PVP at 550 °C. At room temperature, the ionic conductivity of this cathode was 9.6 × 10^−6^ S cm^−1^, and the electronic conductivity 2.2 × 10^−5^ S cm^−1^. The all-solid-state cell with this cathode, lithium–indium anode, and LGPS electrolyte delivered a capacity of 830 mAh g^−1^ (71% utilization of Li_2_S) at 50 mA g^−1^ for 60 cycles at room temperature. The Li_2_S loading was ≈3.6 mg cm^−2^.

A major problem in Li–S batteries is the sulfur loading, which is limited by the insulating character of sulfur, which limits the energy density of the cells with respect to that of commercial lithium-ion cells. However, a high sulfur loading > 7 mg cm^−2^ was achieved by Fu et al., who fabricated a bilayer garnet solid-state electrolyte [466]. The garnet was LLZO, which was doped with both calcium and niobium (Li_7_La_2.75_Ca_0.25_Zr_1.75_Nb_0.25_O_12_) to improve the ionic conductivity (2.2 × 10^−4^ S cm^−1^ at 22 °C). Using the tape casting method, a garnet bilayer was formed that comprised a dense layer and a porous layer. Carbon nanotubes infiltrated the porous layer, and sulfur was loaded by melting sulfur powder into the porous structure at 160 °C. The corresponding Li–S cell delivered a capacity of 645 mAh g^−1^ at a current density of 0.2 mA cm^−2^. This corresponds to an energy density of 248.2 Wh kg^−1^, considering the total mass of the cathode. However, the capacity decreased upon cycling to approximately 450 mAh g^−1^ after 30 cycles, either because the sulfur loading was too high or because the shuttle effect was not entirely suppressed. The template method used in reference [463] to fabricate a porous garnet structure was also employed by Gong et al. to synthesize a LLZO-based textile structure, and tape-casting was also used to fabricate the dense supporting layer [467]. The template process consists of soaking a textile template with the ceramic precursor solution, followed by pyrolysis to eliminate the organic part. The advantage of the textile structure is that it is flexible. LiTFSI/PEO infiltrated the textile and the open pores were filled with S/CNTs to obtain a garnet textile reinforced composite polymer electrolyte. The corresponding Li-S battery loaded with 10.8 mg cm^−2^ sulfur cycled at 0.15 mA cm^−2^ delivered 1250 mAh g^−1^ at the fifth cycle, which decreased to 1000 mAh g^−1^ at the 40th cycle. The authors specified that a small amount of liquid electrolyte was added to obtain this result. Higher sulfur loadings resulted in faster decreases in the capacity upon cycling, proving that this is the crucial parameter that limits the cycle stability.

### 6.2. Polymers

Considering the energy density, the use of solid-state electrolytes poses a problem for Li–S batteries, as the density of solid-state electrolytes is at least 2–5 g cm^−3^, which will also contribute to lowering the energy density [454]. This is why more attention has been focused on SPEs, as their density is close to 1 g cm^−3^, which is close to that of liquid electrolytes. However, the drawback of the SPEs is again the low ionic conductivity. Among them, PEO-LiTFSI has been extensively studied, like in the case of lithium-ion batteries. It is even possible to increase the ionic conductivity of this SPE by adding halloysite nanotubes (HNTs) to obtain a homogeneous PEO + LiTFSI (EO:Li = 15:1) + HNT (10%) electrolyte that reveals a conductivity of 1.11 × 10^−4^ S cm^−1^ and a lithium ion transference number of 0.40 at 25 °C, which are sufficient to obtain a Li–S cell that delivers 745 ± 21 mAh g^−1^ after 100 discharge/charge cycles at 0.1C, with 87% retention, compared to the second discharge capacity [468]. Halloysite is an aluminosilicate (Al_2_Si_2_O_5_(OH)_5_) natural nanoclay with a silica outer surface; therefore, the origin of the gain in conductivity is the separation of the lithium salt into lithium ions that are absorbed on the negatively charged outer silica surface, and the anions may be accommodated on the positively charged inner aluminol surface. However, we have already mentioned that this is a dual-ion conducting system, which implies a low transference number. In the lithium–sulfur chemistry, there is an additional problem with this SPE, namely, PEO complexes the alkali metal salts and can thus dissolve the lithium polysulfides, which will contribute to the “shuttle effect” [469]. To reduce the shuttle effect, inverse vulcanization of sulfur [470,471] and inverse vulcanized sulfur (p(S-DVB)) copolymers [472] were proposed. In particular, the Li–S cell comprising the optimized p(S-DVB) cathode (80:20 w/w S/DVB ratio) and LiFSI/PEO electrolyte shows a high specific capacity (ca. 800 mAh g^−1^) and a high Coulombic efficiency over 50 cycles.

The sulfur cathode prepared by polymerization of aniline to form macro-polyaniline (PANI) mixed with sublimed sulfur and conductive carbon was combined with PANI-MIL-53(Al)-LiTFSI electrolyte [473]. After 1000 cycles at 4C, 80 °C and 0.5C, 60 °C, the discharge capacities of 325 and 558 mAh g^−1^ were obtained, respectively. The sulfur loading, however, was only 0.8 mg cm^−2^, which was too low for this cell to be competitive with lithium-ion batteries in term of energy density. Nevertheless, this result proves that sulfur is stably linked to PANI, which inhibits polysulfide dissolution and shuttling in all-solid-state Li-S batteries. Moreover, the modification of PANI by the porous metal–organic MIL-53(Al) framework was efficient to improve the ionic conductivity, which explains the good rate capability, and it was applied in the development of other electrolytes such as PEO-MIL-53(Al)-LiTFSI electrolyte for other types of batteries [474].

Improved cyclability was achieved with a starch-hosted electrolyte obtained using DMSO solvent and (3-glycidyloxypropyl)trimethoxysilane (KH-560) reactant [475]. After crosslinking and mixing with LiTFSI, this electrolyte in a Li-S cell delivered 864 ± 16 mAh g^−1^ at 0.1C for 100 cycles, 562 ± 118 mAh g^−1^ at 0.5C for 1000 cycles at room temperature, and 388 ± 138 mAh g^−1^ for 2000 cycles at 2C and 45 °C.

The works we have cited provide evidence for the fact that LiTFSI is traditionally used as the lithium salt in Li–S cells. However, Li–S cells containing LiFSI/PEO electrolyte have shown much better cycling performances, compared to those of cells with conventional LiTFSI/PEO, as a LiFSI/PEO cell delivers a high specific discharge capacity of 800 mAh g_sulfur_^−1^ and a high areal capacity of 0.5 mAh cm^−2^, with good rate capability and cyclability over 50 cycles [476]. The cell performance was then improved by combining two LiFSI/PEO polymer electrolytes, one containing a glass-ceramic and the other containing Al_2_O_3_ filler, in which case, the capacity was increased to 518 mAh g^−1^ and 0.53 mAh cm^−2^, with a Coulombic efficiency higher than 99% at the end of 50 cycles at 70 °C [477].

A lithiated Nafion (Li-Nafion) membrane swollen with PC (PC-Li-Nafion) was also proposed. The drawback is its low conductivity, which is 2.1 × 10^−4^ S cm^−1^ at 70 °C, but, the lithium-transference number is very high (0.95). When a thin-layer Li-Nafion resin with a thickness of approximately 2 μm is intercalated between the cathode and PC-Li-Nafion membrane to improve the interfacial contact, the Li–S battery delivered reversible specific capacities of 1072.8 mAh g^−1^ at 0.05C and 895 mAh g^−1^ at 1C at this temperature. The capacity retention at 1C was 89% after 100 cycles [478]. Note, however, that Li-Nafion, which has also been tested in Li–O_2_ batteries [433], is expensive and that pure Li-Nafion alone cannot be used as an electrolyte because its mechanical strength is too low.

A sandwiched GPE PVDF/PMMA/PVDF as a separator was synthesized by Yang et al. [479]. The outer PVDF layer is porous, which helps the ether-based electrolyte to pass through and then enhance the Li^+^ transfer; the inner PMMA layer is a solid film, which exhibits a good compatibility with the ether-based electrolyte and traps the dissolved Li_2_S_x_ (4 ≤ *x* ≤ 8), and was impregnated with LiTFSI salt in the solvent DME-DOL (1:1). DOL is commonly used in Li–S batteries as it reduces the electrolyte viscosity and prevents the corrosion of the lithium metal electrode. This separator can not only reduce the shuttle effect and thus improve the cycling life, but also improve the utilization of sulfur, which significantly results in a higher capacity with respect to those of commercial separators.

The multilayer design used in the previous example involving a GPE layer is a good strategy for suppressing the shuttle effect. Solid electrolytes are recognized as the ultimate approach to eliminating the shuttle effect; nevertheless, the challenge is to maintain the contact between the electrolyte and the electrodes. Indeed, the addition of a GPE buffering layer is also a good strategy to reduce the interface resistance, and, as the liquid electrolyte is immobilized in the GPE, the benefit of the solid electrolyte is retained, except for the flexibility. Another example is the gel-ceramic multilayer electrolyte fabricated by Wang et al. that consists of a LAGP layer (of thickness 0.6 mm) and a PEO-based GPE (a gel-forming liquid electrolyte of 1 mol L^−1^ LiTFSI in TEGDME) [480]. A remarkable result was obtained with a pentaerythritol tetraacrylate-based GPE, which displayed an outstanding ion conductivity of 1.13 10^−2^ S cm^−1^ and a transference number *t*_Li+_ of 0.47 [481]. The Li–S cell with this GPE and sulfur loading of 1.2–1.5 mg cm^−2^ delivered a capacity of 529.7 mAh g^−1^ after 400 cycles at 0.5C, which corresponds to a capacity retention of 82%. Owing to the high conductivity, the cell still delivered a capacity of 600 mAh g^−1^ at 5C.

A nanoscale microfibrillated cellulose-laden polymer system was synthesized by Nair et al. [482]. Here, the polymer was based on methacrylic oligomers and reinforced with raw nanoscale cellulose fibers, where the crosslinked polymer matrix acts as a cage to trap the liquid electrolyte comprising a 0.75 mol L^−1^ LiTFSI solution in a 1:1 (v/v) mixture of TEGDME, DOL, and lithium nitrate (0.5 mol L^−1^). Like PMMMA, TEGDME shows a high solubility for polysulfides, and LiNO_3_ is added to improve the sulfur utilization and cyclability, as it forms a sturdy protective SEI film on the lithium surface by inhibiting their reduction on lithium metal. On the other hand, the formation of ionic couples/aggregates that result from the excess number of mobile ions formed by the addition of LiNO_3_ was responsible for the rather limited value of the transference number (0.35). Nevertheless, owing to the high ionic conductivity (1.2 mS cm^−1^ at 20 °C), stable cycling at approximately 730 mAh g^−1^ was achieved. The sulfur loading, however, was only 42 wt.% (the cathode consisted of 70 wt.% sulfur-activated carbon, which contained 60 wt.% sulfur).

The other strategy is to look for other SPEs and efforts are currently being taken to design SIPEs to eliminate lithium dendrites and reduce the dissolution of lithium polysulfides [483]. A SIPE was fabricated for a sodium-ion battery by Pan et al. with a membrane made of sodium ion exchanged poly(bis(4-carbonyl benzene sulfonyl)imide-co-2.5-diaminobenzenesulfonic acid) macromolecules that were blended with PVDF–HFP [484]. The ionic conductivity of this membrane was 0.91 × 10^−4^ S cm^−1^ at 20 °C and 4.1 × 10^−4^ S cm^−1^ at 80 °C (there must be a solvent/plasticizer added). Therefore, it is not yet competitive with the Na-NAFION film, which is more conductive with a larger ion density, but it proves that a SIPE is also possible for sodium-ion batteries.

To improve the electrochemical properties, SIPE-based GPEs have been proposed, rather than single-ion conducting polymer solid electrolytes. Pan et al. replaced 4-amino-4’-trifluoromethyl bis(benzene sulfonyl)imide used in the work reported in reference [227] for lithium batteries with lithium 4-amino-phenyl sulfonyl(trifluoro methyl sulfonyl)imide (LiATFSI) as the material that was grafted with poly(ethylene-alt-maleic anhydride) (Mw = 100 000-500 000) to obtain a single-ion conducting polymer (EMA-graft-LiATFSI) blended with PVDF–HFP. The gel polymer obtained by impregnation of this membrane with EC/DMC was used as a GPE to obtain a Li–S cell that delivered a capacity of 780.8 mAh g^−1^ at the 1000^th^ cycle at 1C rate [485]. The result, however, was obtained with a rather low sulfur loading of 1.6 mg cm^−2^.

Polysiloxane electrolyte reveals a good electrochemical stability, but poor ionic conductivity, which can be improved by adding a high concentration of lithium salt. Such a “polymer-in-salt” was fabricated with the polysiloxane (BPSO) electrolyte, LiTFSI, and PVDF. As the addition of salt is detrimental to the mechanical properties, CA was used as a framework [486]. The 90% (BPSO-150% LiTFSI)-10% PVDF + CA composite electrolyte displayed a conductivity of 4 × 10^−4^ S cm^−1^, transference number of 0.52, and stability window of 4.7 V, with the mechanical strength being 6.8 MPa. The Li–S cell constructed with this electrolyte with a sulfur mass loading of 2.0–3.0 mg cm^−2^ delivered a capacity of 910 mAh g^−1^ at the second cycle at 1C, and approximately 460 mAh g^−1^ after 80 cycles.

Other remarkable results have been obtained for Li–S batteries with sulfur-based ceramics, without involving metal sulfide cathodes. With a rGO@S–Li_10_GeP_2_S_12_–acetylene black composite, a capacity of 903 mAh g^−1^ was obtained at 1C at 60 °C, which was maintained at 830 mAh g^−1^ after 750 cycles [487]. Most of all, Li_7_P_3_S_11_ glass-ceramic solid electrolyte with a high ionic conductivity and a composite cathode made of sulfur and BP2000 porous carbon (S@BP2000) with a core-shell structure were used to fabricate a novel all-solid-state sulfur battery [488]. Owing to the ionic conductivity of Li_7_P_3_S_11_ (2 × 10^−3^ S cm^−1^ at room temperature, the cell delivered a capacity of 1391 mAh g^−1^ at 0.2C and 677 mAh g^−1^ at 5C, and a capacity retention of nearly 100% after 1200 cycles was obtained, for a mass loading of the cathode of 2 mg cm^−2^.

Xu et al. synthesized another glass ceramic Li_7_P_2.9_Mn_0.1_S_10.7__0.3_ by high-energy ball milling. This ceramic demonstrated a good compatibility with lithium, and an ionic conductivity of 5.6 mS cm^−1^ at room temperature [489]. The all-solid-state S-C-Li_7_P_2.9_Mn_0.1_S_10.7_|_0.3_/Li_7_P_2.9_Mn_0.1_S_10.7_|_0.3_/Li battery at room temperature delivered capacities of 604 mAh g^−1^ at 0.1C and 412 mAh g^−1^ at 0.2C, compared to 682 and 574 mAh g^−1^, respectively, for the liquid cell. The capacity of the all-solid-state battery was stable during the 60 cycles that it was tested.

Other sulfur-based solid-electrolytes have been considered. Using PAN-sulfur (PAN-S) reaction product as the cathode and the same PCE interlayer as that used for the lithium battery with LiFePO_4_ cathode, Wang et al. fabricated an all-solid-state Li-S battery, PAN-S/PCE-LGPS-PCE/Li, that delivered at room temperature a second discharge capacity of 890 mAh g^−1^, which was retained at 775 mAh g^−1^ after 100 cycles [310]. Suzuki et al. injected LGPS into the pores of sulfur carbon replica composite through immersion of LGPS and the sulfur carbon replica in THF, which was followed by mixing and drying [490]. The thus-obtained composite cathode resulting from the combination of liquid and mechanical mixing was used as a cathode in a cell with lithium–indium counter electrode and LGPS electrolyte and exhibited a capacity larger than 1500 mAh g^−1^ at 0.5C, and 1200 mAh g^−1^ after 50 cycles, when the cell was compressed at 213 MPa during the charge–discharge measurements to maintain its integrity, despite the volume change occurring during cycling. This result shows that this process is quite efficient in optimizing the contact between the active cathode particles and the solid-state electrolyte of the Li–S battery.

Choi et al. have fabricated a Li–S battery with Li_4.4_Si anode, Li_2_S-P_2_S_5_ solid electrolyte, and sulfur + acetylene black + solid electrolyte cathode [491]. They employed secondary ball milling to improve the electrolyte/cathode interfacial area, but, even so, significant capacity fading was observed over the six cycles tested. Actually, Li_2_S-P_2_S_5_ decomposes into Li_2_S and Li_3_P at the surface of lithium [492] and cannot be used in direct contact with this anode. However, it can be used with lithium–indium, and Hakari et al. obtained outstanding results with a Li/Li_2_S all-solid-state battery that consisted of Li-In/80Li_2_S·20P_2_S_5_ electrolyte/Li_2_S-LiI. The 80Li_2_S·20P_2_S_5_ solid solution is decomposed to form LiI dispersed in the Li_2_S matrix. Then, the conversion reaction of Li_2_S/S without the formation of liquid electrolyte-soluble polysulfides proceeds in the all-solid-state Li/S cell. The formation of LiI provides electrochemical reaction sites because LiI exhibits a relatively high ionic conductivity. This cell at room temperature delivered capacities of 1100 mAh g^−1^, which corresponded to 95% utilization of sulfur at 0.5 C, and 980 mAh g^−1^ at 2C for 2000 cycles without any degradation [493]. As usual, in the literature, the capacities are reported per gram of the active element, i.e., sulfur in Li_2_S here. The gravimetric energy density per gram of this cell was not specified but will certainly be lower than that of a commercial lithium-ion battery, because lithium–indium is a heavy anode fabricated from a scarce element and the voltage of the Li–S cell is low. However, this is a major improvement for all-solid-state Li-S batteries, where at most 75% of the sulfur could be used so far, owing to its insulating nature. It is a step toward high-performance Li-S batteries based on Li_2_S cathodes [494].

Actually, the best ionic conductivity of Li_2_S-P_2_S_5_ of 1.58 × 10^−3^ S·cm^−1^ is not obtained with the composition 80Li_2_S·20P_2_S_5_ chosen by Akari et al., but with the composition 70Li_2_S·30P_2_S_5_, owing to the precipitation of the high-conductivity thio-LISICON III phase and the heat treatment, which reduced the grain boundary [495]. Using commercial Li_2_S powders (30 wt.%), 70Li_2_S·30P_2_S_5_ glass-ceramic (60%), and carbon as the composite cathode, 70Li_2_S·30P_2_S_5_ as the electrolyte, and lithium–indium anode, the Li–S cell delivered a second discharge capacity of 811 mAh g^−1^, but at the extremely slow rate of 0.01C, and the capacity retention was still only 57% after 10 cycles, a result that the authors attributed to the two large particles of sizes 10–20 μm of the commercial Li_2_S.

Zhang et al. focused their attention on the cathode to improve both its ionic and electrical conductivities, and its interface with the solid electrolyte [496]. First, sulfur was in situ precipitated onto the surface of graphene oxide (GO) grafted with an electrolyte composed of PEG. Sulfur and conductive carbon (Super-P) were then in situ precipitated onto the substrate surface to form a GO–PEG@C/S cathode. GO, with many reactive functional groups on the surface, could trap the polysulfides, and the presence of PEG enhanced the ionic conductivity; Super-P was utilized here to buffer the volume change during cycling and enhance the electrical conductivity. A metal–organic framework (MIL-53(Al)) was used to prevent polysulfide dissolution and shuttling and form PEO–LiTFSI–MIL-53(Al)–acetonitrile electrolyte. The corresponding cell with lithium anode was tested at 80 °C. Capacities of 613 and 444 mAh g^−1^ were obtained at 1C and 2C, respectively, after five cycles. After 100 cycles, the discharge capacities were 531 mAh g^−1^ at 1C and 380 mAh g^−1^ at 2C, with capacity retentions of 86.6% and 85.6%, respectively.

A remarkable result has been obtained recently by Kim et al. [497]. These researchers reported a novel closo-borane lithium superionic conductor, 0.7Li(CB_9_H_10_)-0.3Li(CB_11_H_12_), with excellent stability against lithium metal and a high conductivity of 6.7  ×  10^−3^ S·cm^−1^ at 25 °C. It exhibited a stable lithium plating/stripping reaction with the interfacial resistance < 1 Ω cm^2^. The high conductivity arises from the stabilization of the high-*T* disordered phase of Li(CB_9_H_10_) by the partial replacement of (CB_9_H_10_)^−^ with (CB_11_H_12_)^−^. A solid-state Li-S battery was fabricated with this electrolyte and a sulfur-carbon cathode, with a theoretical capacity of 1672 mAh g^−^^1^ of the electrode. As the theoretical capacity of sulfur is 3860 mAh  g^−^^1^, we can deduce that sulfur weighted 43.3% of the total weight of the cathode. For a discharging rate of 3C and a charging rate of 1C at 50 °C, the discharge capacity was 1533 mAh g^−^^1^ in the second cycle, which dropped to 1469 mAh g^−^^1^ after 20 cycles, leading to high energy densities of 2578–2782 Wh kg^−1^. This is the best energy density obtained so far for a solid-state Li-S battery, but, most of all, this is also better than the energy densities obtained with Li-LiCoO_2_ [64,84,498], Li–LiNi_0.5_Mn_1.5_O_4_ [499], and Li-Li_2_FeMn_3_O_8_ [131] all-solid-state batteries, which is proof that the Li-S cell can overcome its handicap of a low operating voltage (ca. 2.1 V) relative to those of high-voltage lithium-ion batteries.

### 6.3. Substitution of Sulfur for Selenium

Li et al. introduced selenium in the sulfur cathode and fabricated a SeS_2_/LGPS-Li_3_PS_4_/Li solid-state cell that delivered a high capacity of over 1100 mAh g^−1^ (98.5% of its theoretical capacity) at 50 mA g^−1^ that remained highly stable for 100 cycles [500]. As selenium is a functional material that displays essentially the same properties as sulfur, except for the nutritional properties of selenium at very low doses (it is toxic at higher doses), it is tempting to substitute sulfur for selenium. There are some advantages to this substitution [501]. First, selenium is much more conductive than sulfur (1 × 10^−5^ S cm^−1^ against 5 × 10^−30^ S cm^−1^). Selenium exhibits a melting point that is higher than that of sulfur; therefore, the Li–Se battery would be safer than the Li–S battery. This is the motivation for the recent interest in Li–Se batteries at the laboratory scale. Moreover, selenium-doping promotes the solid-state (de)lithiation chemistry of selenium–sulfur cathodes, switching from the conventional two-step solid–liquid–solid reaction to solid-state (de)lithiation by directly bypassing the formation of soluble polysulfides/polyselenides [502]. Owing to all these properties, the selenium-doped S_22.2_Se/Ketjenblack cathode fabricated in this work delivered a reversible capacity of above 1000 mAh g^−1^ at C/20 over 50 cycles, which was 700 mAh g^−1^ at 5C. The rate capability was also enhanced, as the capacity was still 660 mAh g^−1^ after 250 cycles at 1C and 583 mAh g^−1^ after 250 cycles at 2C. However, there are also disadvantages with the use of selenium. The volumetric capacity is similar for both the materials, but the theoretical gravimetric capacity of selenium is only 678 mAh g^−1^ (1675 mAh g^−1^ for sulfur). In addition, selenium is 4000 times rarer than sulfur, and it is of course much more expensive. Therefore, the practical chance of developing a Li–Se cell at the industrial scale is limited, unless selenium is used as a catalyst to improve Li–S cells. The performances of Li–Se cells, nevertheless, are attractive. A Li–Se cell with a hybrid electrolyte consisting of LAGP ceramics sandwiched by 1 mol L^−1^ LiTFSI in TEGDME in the positive electrode and 1 mol L^−1^ LiTFSI + 2 wt.% LiNO_3_ in TEGDME in the negative lithium electrode delivered 677 mAh g^−1^ at 0.1C, 613 mAh g^−1^ over 500 cycles at 0.8C (fading rate = 0.008% per cycle), and 540 mAh g^−1^ at 1.5C [503]. The selenium loading was 1–1.5 mg cm^−2^ only, but a stable discharge capacity of 454 mAh g^−1^ was still obtained when the selenium loading was 5.9 mg cm^−2^. Another Li–Se battery was demonstrated by Li et al., who used Se-Li_3_PS_4_-C as the cathode, Li_3_PS_4_ as the electrolyte, and a lithium–tin alloy as the anode [504]. This Li–S cell delivered a capacity of 652 mAh g^−1^ at a current density of 50 mA g^−1^, with 90% capacity retention after 100 cycles at room temperature.

### 6.4. Use of Transition-Metal Sulfides

Usually, transition-metal sulfides show excellent compatibilities with solid sulfide electrolytes [79,505,506]. They exhibit better reversibility compared to those of their oxide counterparts. The reason is that the M–S bonds are weaker than the M–O bonds, therefore, they can alleviate the volume change caused by the insertion of sodium ions, which is more difficult with metal oxides. In addition, the capacity of the metal sulfides is large. Fe_1-*x*_S nanostructures delivered a reversible capacity of 563 mAh g^−1^ after 200 cycles at a current density of 100 mA g^−1^ within the voltage range 0–3 V vs. Na^+^/Na [507]. A carbon nanotube (CNT)-encapsulated Fe_1-*x*_S composite (Fe_1-*x*_S@CNTs) as the active material was used as the cathode in a Li–S battery and exhibited a capacity of 123 mAh g^−1^, based on the cathode mass [508].

Another metal sulfide that has been considered for Li–S batteries is NiS. Nickel sulfide-anchored CNT (NiS-CNT) nanocomposites were successfully synthesized by hydrothermal method. The Li/75%Li_2_S-24%P_2_S_5_-1%P_2_O_5_/Li_10_GeP_2_S_12_/NiS-CNT all-solid-state batteries delivered a capacity of 515 mAh g^−1^ after 30 cycles at a current density of 0.1 A g^−1^, and maintained a capacity of 170 mAh g^−1^ after 150 cycles at the current density of 1 A g^−1^ [509]. The high rate capability is attributable to the nanosize (10 nm) of the NiS-CNT nanocomposite, which enhances the effective surface area. For comparison, 50 nm thick highly crystalline layered VS_2_ nanosheets in Li/75%Li_2_S-24%P_2_S_5_-1%P_2_O_5_/Li_10_GeP_2_S_12_/VS_2_ all-solid-state battery showed a high reversible capacity of 532.2 mAh g^−1^ after 30 cycles at the current density of 50 mA g^−1^. This capacity was maintained at 437 and 270 mAh g^−1^ at 100 and 500 mA g^−1^, respectively, after 100 cycles [510].

**Figure 14:**
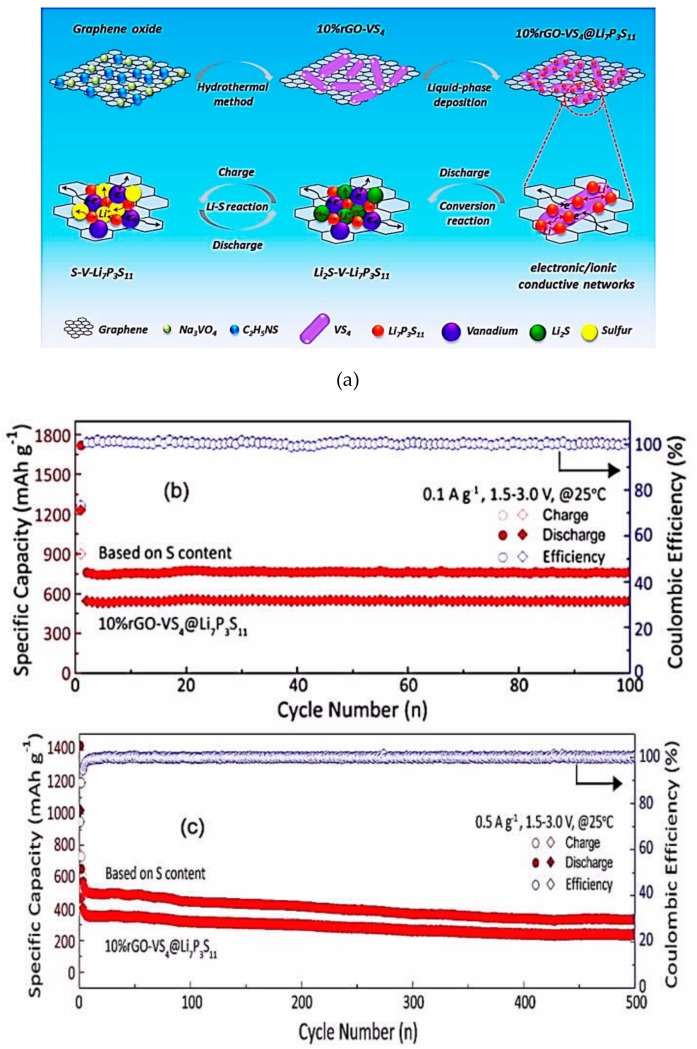
(**a**) Illustration of the synthesis process of 10%rGO-VS_4_ and 10%rGO-VS_4_@Li_7_P_3_S_11_ cathodes. Electrochemical performances of Li/75%Li_2_S-24%P_2_S_5_-1%P_2_O_5_/Li_10_GeP_2_S_12_/10%rGO-VS_4_@Li_7_P_3_S_11_ all-solid-state batteries at room temperature and at the current densities of (**b**) 0.1 A g^−1^ and (**c**) 0.5 A g^−1^. Reproduced with permission from [508]. Copyright 2019 Elsevier.

Remarkable results were obtained with another metal sulfide, VS_4_. Part of the interest in VS_4_ is its structure, as it exhibits a structure with parallel quasi-1D chains of V^4+^(S_2_^2−^)_2_, which reveal a large interchain distance of 5.83 Å, which offers the possibility of a high capacity based on the redox chemistry of vanadium and the cleavage of the S–S bonds. Wang et al. found that rGO-supported cuboid-shaped VS_4_ nanoparticles in Li-S battery delivered a capacity of ≈580 mAh g^−1^ at 0.1 A g^−1^, a long cycle-life (≈98% was retained at 0.5 A g^−1^ after 300 cycles), and high rates (up to 20 A g^−1^) [511]. To increase the electrical conductivity of the sulfur cathode, Zhang et al. used linear-chain VS_4_-anchored rGO nanosheets prepared by one-pot hydrothermal method. The rGO-VS_4_ nanocomposites were in situ coated with Li_7_P_3_S_11_ solid electrolyte to improve the interfacial contact. In addition, VS_4_ can transform into metallic vanadium nanoparticles and Li_2_S, based on a conversion reaction, after the first discharge. Then, Li_2_S delithiates into amorphous elemental sulfur, while the metallic vanadium increases the electrical conductivity [512]. The all-solid-state lithium battery Li/75%Li_2_S-24%P_2_S_5_-1%P_2_O_5_/Li_10_GeP_2_S_12_/10%rGO-VS_4_@Li_7_P_3_S_11_ delivered a capacity of 611 mAh g^−1^ at a current density of 0.1 A g^−1^ between 0.5 and 3.0 V after 100 cycles (Figure 14). Moreover, it exhibited a superior cycling stability and reversible capacities of 238 and 333 mAh g^−1^ after 500 cycles, based on the mass of 10%rGO-VS_4_@Li_7_P_3_S_11_ and sulfur content, respectively. At the current densities of 0.1 and 1.0 A g^−1^, the energy density was 545 Wh kg^−1^ and the power density 789 W kg^−1^, respectively, based on the weight of the composite electrode (we need additional values for the two current densities). This represents a remarkable performance that is even higher than the results obtained with LiCoO_2_ and Co_9_S_8_/Li_7_P_3_S_11_ cathodes in all-solid-state lithium batteries [513]. In another work, Pang et al. showed that VS_4_ anchored on graphene sheets delivered a large specific capacity (349.1 mAh g^−1^ after 100 cycles), with 84 % capacity retention after 1200 cycles at a current density of 100 mA g^−1^ [514]. Flower-like vanadium sulfide/rGO was also investigated [515]. Among the different morphologies that have been investigated, the electrochemical performance degrades in the order of urchin-like VS_4_ > octopus-like VS_4_ > sea grass-like VS_4_ > flower-like VS_4_ [516]. In addition, the S_2_^2−^ dimers in the VS_4_ nanodendrites provide abundant sites for Mg^2+^ insertion, and VS_4_ has been used as a cathode in a magnesium battery, with the discharge capacity being 251 mAh g^−1^ at 100 mA g^−1^ and the capacity being 74 mAh g^−1^ after 800 cycles at a current density of 500 mA g^−1^ [517].

## 7. Sodium-Ion Batteries

US and Japanese companies developed NIBs in full cell configurations in the 1980s, wherein sodium–lead alloy composites and P2-type Na*_x_*CoO_2_ were used as the negative and positive electrodes, respectively, [518,519,520] even before the commercialization of LIBs. Indeed, full cells are easier to build than half-cells, because sodium metal, which is used as a counter electrode, is more highly reactive than lithium metal. Currently, some papers have reported the electrochemical properties of cells with sodium metal negative electrode, while others refer to the use of sodium insertion material as the negative electrode, which makes the comparison between them more difficult. Several reviews have been published that focused on the electrode materials [521], salts and solvents [445], and electrolytes [522]. Not surprisingly, the experience with lithium batteries has been used, and the electrolytes envisioned for sodium batteries are mainly the same, except for the ion carriers. They involve ionic liquids [523,524,525,526,527,528,529,530], NASICON [531], composites [532,533,534,535,536], polymers, and GPEs [484,537,538,539], including single-ion conductors [484]. In principle, there are some fundamental advantages with the application of polymer electrolytes in a sodium-ion battery, as compared to their application in a lithium system. In particular, the smaller cation–anion interactions in many sodium salts result in more efficient creation and transport of Na^+^ carriers in gel-polymer systems, where liquid electrolytes are trapped in the polymer matrices. In particular, PEO/NaTFSI has been used by different researchers [536,540,541]. NaTFSI can be chosen for the same reason as LiTFSI has been chosen in lithium batteries with PEO-based electrolytes, namely, the high degree of charge delocalization of [TFSI]^−^, which leads to much weaker ion–ion interactions and thereby increased dissociation and solubility of [TFSI]^−^ in PEO matrix. Another choice of sodium salt is NaClO_4_. In particular, a PEO-based crosslinked polymer electrolyte prepared by UV curing that included NaClO_4_ as the sodium salt dissolved in PC was tested for application in sodium-ion batteries [542]. This polymer electrolyte showed a very good conductivity > 1 mS cm^−1^ at room temperature, an electrochemical window that extended up to 4.7 V, and a transference number *t*_Na+_ of 0.32. The corresponding cell with TiO_2_ and sodium electrodes displayed an average operating voltage of 0.92 V. At a low current density of 0.1 mA cm^−2^, after five cycles, which were needed for equilibration, the cell delivered a capacity of 260 mAh g^−1^. At the current density of 0.5 mA cm^−2^, the initial capacity was 100 mAh·g^−1^, 60% of which was retained after 5250 h (corresponding to 1000 reversible cycles). NaClO_4_ was also chosen as the salt in PEO blended with TiO_2_ [533]. The capacity delivered by the half-cell with Na_2/3_Co_2/3_Mn_1/3_O_2_ cathode was stable over the 20 cycles that it was tested. However, the capacity was very low, presumably because the membrane was too thick (0.18 mm). Therefore, additional experiments over a greater number of cycles with a thinner membrane are needed before any conclusion can be drawn. Bitner-Michalska et al. proposed a fluorine-free electrolyte based on percyano-substituted organic salts in PEO [543]. Among them, sodium 2,3,4,5-tetracyanopyrrolate (NaTCP) is promising, as the solid electrolyte NaTCP/PEO_14_ shows an ionic conductivity larger than 1 mS cm^−1^ at 70 °C. Only the stability window (5 V vs. Na^+^/Na) has been studied so far.

The interesting results obtained for lithium batteries with PEO-polyurethane combination [258] suggested that PEO-based polyurethane sulfonate ionomers incorporating NaSO_3_ ionic group might work. This material was studied by Wang et al. for different ratios of Na^+^/EO, but they showed that only 2% of the Na^+^ contributed to the conductivity at any given time [542].

An 80% polycaprolactone-20% polytrimethylene carbonate (PCL–PTMC) copolymer with LiTFSI was used some time ago as a solid electrolyte for lithium-ion batteries, and revealed an ionic conductivity of 4.1 × 10^−5^ S cm^−1^ at 25 °C and a high cation transference number of 0.62 at 40 °C [544]. More recently, the same copolymer was used with NaTFSI for application in a sodium-ion battery [545]. With 25% NaTFSI, this electrolyte showed a conductivity of 3.9 × 10^−6^ S cm^−1^ at 25 °C, but the transference number decreased to 0.47 upon switching from lithium to sodium. The tests conducted with a hard carbon (HC) anode and Prussian blue (Na_2-x_Fe[Fe(CN)_6_]) cathode were not conclusive in the sense that the capacity decreased rapidly, but this was expected, given the choice of HC anode. The same group has shown stable cycling of a Na|PTMC:NaFSI|(Na*_x_*Fe[Fe(CN)_6_]) cell that was subjected to 80 consecutive cycles; an average discharge capacity of 90 mAh g^−1^ at 60 °C was obtained [546], which confirmed the capability of the PTMC-NaFSI combination to be applied in sodium-batteries, provided that the salt concentration is optimized.

Na^+^ ceramic electrolytes and NASICON with Na_1+3x_Zr_2_(P_1-x_Si_x_O_4_)_3_ structure exhibited an ionic conductivity > 10^−3^ S cm^−1^ at *T* = 65 °C [532]. An even better conductivity of 4.0 × 10^−3^ S cm^−1^ at room temperature was observed through optimized scandium substitution leading to the composition of Na_3.4_Sc_0.4_Zr_1.6_(SiO_4_)_2_(PO_4_) [547], but there is no chance of utilizing scandium in an industrial process. Bulk and ionic conductivity values of 1.43 × 10^−3^ and 1.10 × 10^−3^ S cm^−1^ were obtained in Na_3+x_Zr_1.9_La_0.1_(SiO_4_)_2_(PO_4_) at room temperature, owing to the doping of La^3+^ as a substitute of Zr^4+^, which improved the mobility of the Na^+^ [548]. A PVDF matrix with NASICON-type Na_3_Zr_2_Si_2_PO_12_ and NaClO_4_ as the sodium salt has also been used as an electrolyte in a half-cell with Na_0.67_MnO_2_ cathode [549]. The interface between this solid electrolyte and sodium anode was modified with a minute dose (2 μL) of a liquid electrolyte (1 mol L^−1^ NaClO_4_/PC/5 vol % FEC), which is necessary to improve the interfacial contact and uniformize the Na^+^ current. The corresponding Na//Na_0.67_MnO_2_ cell delivered a capacity of 96.5 mAh g^−1^ at 0.2C. At 1C, the capacity was still 87 mAh g^−1^, with a retention of 94% after 100 cycles. This result provides evidence that this electrolyte avoided the dissolution of manganese, which prevented the use of this cathode in liquid electrolytes.

According to DFT and molecular dynamics simulations, mere 2% concentration of sodium-ion vacancies in Na_3_PS_4_ should increase the ionic conductivity to 0.2 S cm^−1^ [550]. Many efforts have therefore been taken to generate vacancies in Na_3_PS_4_ by aliovalent doping. The best experimental result was obtained for the substitution of Cl^−^ for S^2^^−^: An ionic conductivity larger than 1 × 10^−3^ S cm^−1^ was observed at 25 °C for Na_2.9375_PS_3.9375_Cl_0.0625_ [551]. The conductivity obtained through other substitutions did not increase beyond 1.5 × 10^−4^ S cm^−1^ [104,552,553,554,555,556]. A new superionic phase β-Na_3_PS_4_ has been discovered [555], but it has not yet been tested as an electrolyte.

**Figure 15:**
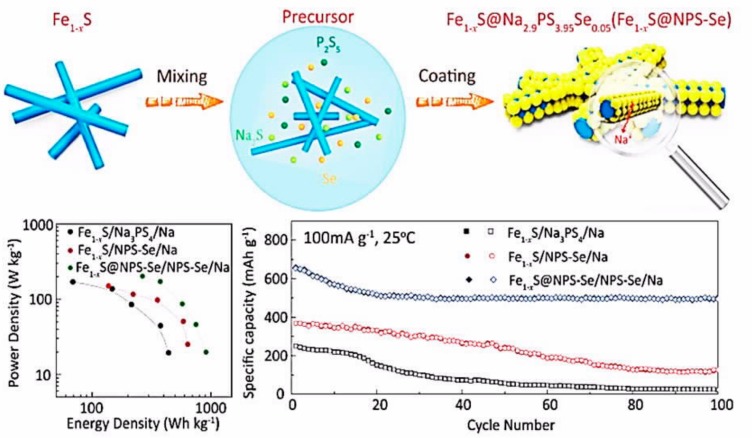
Top: Coating of Na_2.9_PS_3.95_Se_0.05_ (NPS-Se) on Fe_1-*x*_S nanorods to obtain the cathode for sodium-ion batteries. Bottom: Electrochemical properties of the Fe_1-*x*_S@Na_2.9_PS_3.95_Se_0.05_/Na_2.9_PS_3.95_Se_0.05_/Na cell. Reproduced with permission from [556]. Copyright 2018 The American Chemical Society.

We have already mentioned the interest in the substitution of sulfur by selenium in the lithium–sulfur chemical system. This has also found applications in lithium–sodium cells. Substitution at the doping level has been tested in Na_3_PS_4_. Na_2.9_PS_3.95_Se_0.05_ coated on Fe_1-*x*_S nanorods by in situ liquid-phase approach was used as the cathode in an all-solid-state Li–S battery. This coating was carried out as a wet process to improve the contact of the cathode with the electrolyte, which was also Na_2.9_PS_3.95_Se_0.05_. The Fe_1-*x*_S@Na_2.9_PS_3.95_Se_0.05_/Na_2.9_PS_3.95_Se_0.05_/Na cell delivered a capacity of 494 mAh g^−1^ after 100 cycles at a current density of 100 mA g^−1^ and excellent rate capability, with a capacity of 300 Ah g^−1^ at a current density of 300 mA g^−1^ [556] (Figure 15). This cell outperforms the other lithium–sodium cells reported in the literature [557,558,559]. The result also illustrates the efficiency of the liquid-phase method in improving the ionic conductivity through in situ deposition of solid sulfide electrolytes on the surfaces of active materials [79,515,560].

All the conventional solid-state electrolytes that contain phosphorus sulfide are unstable in air [69,104,561]. This was the motivation to substitute phosphorous with stibium and synthesize Na_3_SbS_4_ via the aqueous-solution route [562]. The major advantage of this route is its homogeneity, which permits coating of cathode particles and fabrication of sheet-type electrodes. Kim et al. used Na_3_SbS_4_-coated FeS_2_ cathode and Na_3_SbS_4_ as the solid electrolyte to obtain a sodium cell with the discharge and charge capacities of 324 and 256 mAh g^−1^, respectively, for the first cycle at 30 °C at 50 μA cm^−2^, when cycled between 0.6 and 3.0 V. This is the range of potentials chosen because the discharging (or sodiation) proceeds through a reversible intercalation reaction via the reduction of the sulfides, and capacity fading is avoided owing to the severe volume changes that occur due to the conversion reaction at lower potentials. However, despite this protective measure, the cell retained only 62% of the initial capacity after 50 cycles. It should be noted that these researchers estimated the ionic conductivity of Na_3_SbS_4_ to be 0.1–0.2 mS cm^−1^ at 25 °C. However, owing to the vacancies at the Na2 sites of the tetragonal lattice, the conductivity increased to 3 mS cm^−1^ [563], which was in agreement with theoretical predictions [564].

In any case, similar to ceramics in lithium cells, an additional stable interfacial interstitial interlayer that is wetted by the anode is mandatory [180,305]; it should display the same functions: Avoid a large interfacial impedance and the formation of anode dendrites [565], but also buffer the large volume changes to maintain contact during cycling. The synthesis, chemistry, and interfacial properties of ionic liquid membranes produced as SEI films that are formed directly on metallic sodium through electro-polymerization of functional imidazolium-type ionic liquid monomers in liquid electrolytes have been reported by Wei et al. [566]. Zhou et al. proposed an interlayer that was formed by heating a Na_3_Zr_2_(PO_4_)(SiO_4_)_2 (_NASICON) pellet with liquid sodium at 380 °C for 30 min. The sodium metal dispersed over the surface of the NASICON pellet, which indicated that the interlayer was wetted by sodium [567]. The 380 °C heat-treated NASICON (H-NASICON) pellets in the presence of sodium metal were then tested in a NaTi_2_(PO_4_)_3_/H-NASICON/Na all-solid-state cell, as NaTi_2_(PO_4_)_3_ has been shown to exhibit a stable capacity during long-term sodiation–desodiation in polymer electrolytes [568]. NaTi_2_(PO_4_)_3_ cathode membranes were prepared with cross-linked poly(ethylene glycol) methyl ether-acrylate (CPMEA) as a Na^+^-conducting binder and carbon black as the electron conductor. At 65 °C, when the conductivity of CPMEA reaches 0.7 × 10^−4^ S cm^−1^, the discharge capacity was approximately 110 mAh·g^−1^ at 0.2 C rate during the first 25 cycles and 75 mAh g^−1^ at 1 C during the following 35 cycles. Comparable results were obtained in reference [567] for NaTi_2_(PO_4_)_3_//Na cells with CPMEA/NASICON double-layer electrolyte, as the cathode films were prepared with the polymer electrolyte as the binder. Zhang et al. chose PEO (*M*_w_ = 600 kg mol^−1^)-NaFSI as the polymer and Na_3.4_Zr_1.8_Mg_0.2_Si_2_PO_12_ as the Na^+^ conducting ceramic filler to obtain a composite membrane with a conductivity of 2.4 × 10^−3^ S cm^−1^ at 80 °C; they used this as an electrolyte between Na_3_V_2_(PO_4_)_3_ cathode and sodium-metal anode, and the resulting cell delivered a capacity of 106 mAh g^−1^ without any loss over 120 cycles [530].

While Li_10_SnP_2_S_12_ was investigated as an electrolyte for lithium-batteries [310], Na_10_SnP_2_S_12_ was synthesized for use in sodium-batteries [569]. Its ionic conductivity is remarkable: 0.4 mS cm^−1^ at room temperature, which rivals those of Na_3_PS_4_ [570] and the best sodium sulfide composites, but it has not been tested yet as an electrolyte. On the other hand, chlorine-doped Na_3_PS_4_ with a conductivity reaching 1.14 mS cm^−1^ was tested as an electrolyte in a cell with TiS_2_ cathode and sodium anode, but only at a low C-rate (C/10), at which the cell delivered a capacity of 80 mAh g^−1^ over 10 cycles; further, the cycle life was not explored beyond 10 cycles [571]. Rao et al. have investigated experimentally and by computational methods the doping of Na_3_PS_4_ with Ge^4+^, Ti^4+^, and Sn^4+^ [558]. The highest conductivity of 2.5 × 10^−4^ S cm^−1^ was obtained for Na_3.1_Sn_0.1_P_0.9_S. The full cell Na_2+2δ_Fe_2-δ_(SO_4_)_3_/Na_3.1_Sn_0.1_P_0.9_S/Na_2_Ti_3_O_7_ cycled at 80 °C between 1.5 and 4 V at 2C delivered 109 mAh g^−1^ of Na_2+2δ_Fe_2−δ_(SO_4_)_3_ close to its theoretical value (113 mAh g^−1^), with the capacity retention being 91% after 50 cycles and 82% after 100 cycles. This good result might also be attributed to the fact that the test was conducted on a full cell rather than on a half-cell, as Na_3_PS_4_ is not stable against sodium metal [572].

Na_11_Sn_2_PS_12_ is a new and promising NASICON with a conductivity of 1.4 mS cm^−1^ and an activation barrier energy for Na^+^ that is as low as 0.25 eV [573]; however, it has not been tested in solid-state sodium batteries. It should be noted that the ionic transport properties are even better than those of Li_11_AlP_2_S_12_ in lithium-ion batteries, which are a conductivity of 8 × 10^−4^ S·cm^−1^ at 25 °C and an activation energy of 25.4 kJ mol^−1^ [574].

Lalère et al. used SPS to assemble all-ceramic dense monolithic sodium-ion batteries based on symmetrical Na_3_V_2_(PO_4_)_3_/Na_3_Zr_2_Si_2_PO_12_/Na_3_V_2_(PO_4_)_3_ (NVP/NZSP/NVP) cells that operate at temperatures up to 200 °C [559]. NVP was used as both the anode and cathode, because it can be either reduced to Na_4_V_2_(PO_4_)_3_ or oxidized to NaV_2_(PO_4_)_3_. The energy density of this cell was approximately 1.0 mAh cm^−2^ at C/10. This is 85% of the theoretical capacity of the material, and the cell was stable over the 30 cycles that it was tested. This is not enough for commercial use, but it is the first time that SPS was used to assemble a sodium cell; therefore, the result is promising for this technique.

A strategy to deal with the strongly reacting sodium metal-sulfide electrolyte interface is to fabricate a passivating layer through the reaction of the conductor with the alkali metal anode that can protect the interface that inevitably forms when an alkali metal deposits back on the anode during the charging cycle. Such a passivating interface that is electronically insulating but ionically conductive can be engineered by introducing select elements and/or compounds that react beneficially with lithium/sodium metal [575,576,577,578]. In particular, this strategy is required for Na/Na_3_SbS_4_ interface [579]. This was achieved by Tian et al. through the formation of a hydrated layer on the surface of Na_3_SbS_4_ [580]. Hydration formed a novel phase (Na_3_SbS_4_·8H_2_O) that partially reacted with sodium metal to form NaH and Na_2_O as passivating products that suppressed the electrolyte decomposition.

SCN, which has already been mentioned for its recent use in lithium batteries, was also utilized for sodium-ion batteries. It cannot be used in contact with the highly reactive sodium anode, but Lu et al. found that it is sufficient to introduce a compact NaF-rich interphase on a sodium surface via chemical reactions between fluoroethylene carbonate-Na+ and sodium metal that resulted in a compatible sodium anode/SCN-based electrolyte interface that reduced the overpotential in a symmetric cell to 150 mV after 4000 h [581]. The corresponding Na–CO2 battery based on the chemical reaction 4Na + 3CO2 ↔ 2Na2CO3 + C delivered a capacity of 7624 mAh g−1 at 50 mA g−1, which was maintained at 2689 mAh g−1 at 500 mA g−1, but the cathode had to be changed after 50 cycles. Nevertheless, the cell recovered the initial capacity after changing the cathode; therefore, the problem in the battery was with the cathode and not with the anode, which proved the efficiency of the surface modification.

Na_3_NH_2_B_12_H_12_ was proposed as a new solid electrolyte for sodium batteries [582]. Its Na^+^ conductivity is 1.0 × 10^−4^ S cm^−1^ at a temperature of 372 K. At this temperature, the cell with the positive electrode of Na_3_NH_2_B_12_H_12_/TiS_2_ (in 50:50 weight ratio), Na_3_NH_2_B_12_H_12_ electrolyte, and the negative electrode of sodium delivered a second discharge capacity of 146 mAh g^−1^ at 0.1C. The capacity was reduced to 102 mAh g^−1^ after 100 cycles and 77 mAh g^−1^ after 200 cycles; the cyclability was still too small for practical use, but importantly, it was an improvement with respect to the results obtained with other [B_10_H_10_]^2−^ and [B_12_H_12_]^2−^ compounds [583,584,585].

A novel Na^+^ diffusion mechanism in mixed organic–inorganic ionic liquid electrolytes has been revealed by Forsyth et al. [586] at high concentrations. Given that the preferred average coordination number of Na^+^ is between 4 and 5, at a high concentration, a substantial fraction of anions is shared by more than one Na^+^. Diffusion of the sodium ions can then occur through their facile exchange at the available anion sites, which leads to a high Na^+^ transference number and stable high-rate electrochemical cycling of sodium cells. This new diffusion mechanism has also been reported in reference [587] based on an investigation of the effect of NaFSI salt concentration in methylpropylpyrrolidinium (C3mpyr) FSI ionic liquid that exploits the good solubility of NaFSI in the ionic liquid. At the high concentration of 50 mol % NaFSI, the Na^+^ transference number increased to 0.3, which is typically the result obtained with the conventional liquid electrolytes. In the same way, earlier experiments with LiFSI in C3mpyrFSI systems showed that a lithium ion concentration approximately 50 mol % produced the best cell performance for lithium battery applications at ambient temperatures in terms of the highest rate performance and stability, compared to those obtained at lower LiFSI concentrations [587]. Singh et al. utilized this new diffusion mechanism to investigate a SPE consisting of a polymer + an ionic liquid and NaTFSI salt [588]. PEO was chosen as the polymer, and 1-butyl-3-methylimidazolium TFSI (40 wt.%) as the ionic liquid. For this optimized ionic liquid concentration, the ionic conductivity was ~ 4.1 × 10^−4^ S cm^−1^, and the Na^+^ transference number ~ 0.39. The sodium cell with this electrolyte and Na_0.7_CoO_2_ cathode delivered a capacity of 138 mAh g^−1^ at room temperature. This illustrates the interest in the combination of polymer + ionic liquid + salt, which was already discussed in the section on lithium batteries. Another example is the electrolyte membranes based on PEO polymer, 1-butyl-3-methyl-imidazolium-methylsulfate (BMIM-MS) ionic liquid, and sodium methylsulfate (NaMS) salt that were investigated by Singh et al. [589]. For the optimized composite (PEO+10 wt.% NaMS and 60 wt.% BMIM-MS loading), the ionic conductivity at room temperature was 1 × 10^−4^ S cm^−1^, and the transference number *t*_Na+_ = 0.46. Unfortunately, this electrolyte has been tested over five cycles only. Another attempt was the GPE comprising 0.5 mol L^−1^ of sodium trifluoromethanesulfonate in the ionic liquid 1-ethyl 3-methyl imidazolium trifluoromethane sulfonate, which was entrapped in PVDF–HFP dispersed with particles of the passive filler Al_2_O_3_ and the active filler NaAlO_2_ [590].

A solid electrolyte consisting of PEO_20_–NaClO_4_–5% SiO_2_–70% ethyl-3-methylimidazolium FSI showed an ionic conductivity that stabilized at 7 × 10^−4^ S cm^−1^ at room temperature and a high Na^+^ transference number of 0.61. The sodium-metal cell with this electrolyte and a hybrid cathode prepared by an yttrium-doped sodium zirconate-carbon composite delivered a capacity of 46.2 mAh g^−1^ after 100 cycles, with a capacity retention of 51% at a current density of 0.05C at ambient temperature [591]. All these results illustrate the difficulty of finding a solid-state sodium battery with a good cyclability when using PEO as the supporting polymer. Another example is NaFSI/PEO with the molar ratio of EO/Na = 20 [533]. The half-cell with this electrolyte and Na_0.67_Ni_0.33_Mn_0.67_O_2_ as the cathode material delivered a capacity of 85 mAh g^−1^ at 0.2C, which reduced to 70 mAh g^−1^ after 30 cycles at 80 °C. Ma et al. chose another salt, namely, fluoro-sulfonyl)(n-nonafluoro butanesulfonyl) imide (Na[(FSO_2_)(n-C_4_F_9_SO_2_)N] (NaFNFSI) [592]. NaFNFSI/PEO (EO/Na = 15) showed an anodic electrochemical stability of 4.87 V vs. Na^+^/Na and a conductivity of 3.36 × 10^−4^ S cm^−1^ at 80 °C. The half-cell with Cu_1/9_Ni_2/9_Fe_1/3_Mn_1/3_O_2_ cathode at this temperature delivered a capacity of 100 mAh g^−1^, with a capacity retention of 70% after 150 cycles.

For PEO, however, better results were obtained without the addition of ionic liquids. Instead, carbon quantum dots (CQDs), with diameters in the range 2.0–3.0 nm, were dispersed in the PEO matrix that was blended with LiClO_4_ and NaClO_4_ for use as electrolytes in lithium and sodium batteries, respectively [593]. At room temperature, the PEO/CQDs-Li electrolyte exhibited a conductivity of 1.39 × 10^−4^ S cm^−1^ and a Li^+^ transference number of 0.48, whereas PEO/CQDs-Na electrolyte exhibited a conductivity of 7.17 × 10^−5^ S cm^−1^ and a Na^+^ transference number of 0.42. At 60 °C, PEO/CQDs-Na-based battery yielded an initial discharge capacity of 101.5 mAh g^−1^, which stabilized at 89.4 mAh g^−1^ after 100 cycles. At the same temperature, the PEO/CQDs-Li-based battery delivered a capacity of 120 mAh g^−1^ at 4C over 200 cycles. The origin of the beneficial effect of CQD fillers is the same as those of other fillers like SiO_2_ [310] and TiO_2_ [594], namely, efficient Lewis acid-based interactions at the interface between polymer and filler, which increase the ionic conductivity, decrease the crystallinity, and enhance the segmental motion of the polymer matrix. The difficulty with nanosized particles is in avoiding their agglomeration. The superior performance of CQDs is attributed to not only the reduction in the particle size to 2–3 nm, which increases the effective interface area, but also the good dispersion achieved through a simple aldol condensation reaction [595].

The NASICON Na_3.4_Zr_1.8_Mg_0.2_Si_2_PO_12_ was incorporated into NaTFSI-PEO_14_ as a filler [596]. This filler increases the ionic conductivity for the same reasons as those mentioned above for the other fillers, but it also reveals two specific properties that display additional effects. First, it is highly conductive per se. Second, the Na^+^ in NASICON can be absorbed by the polymer matrix, considering that PEO permits a high degree of coordination of the cation, which increases the sodium vacancies on the surface of NASICON and facilitates ion transport along the surface region. As a result, a conductivity of 2.8 mS cm^−1^ was obtained at 80 °C when the content of the NASICON filler in the composite polymer was 50 wt.%. The sodium cell at 80 °C with this electrolyte, NVP cathode, and sodium-metal anode delivered a stable capacity of 105 mAh g^−1^ over 80 cycles at 0.2C.

Improved cycle life was realized with a new class of polysulfonamide-supported PEG divinyl ether-based polymer electrolytes via in situ preparation [597]. This electrolyte was characterized by a conductivity of 1.2 mS cm^−1^ at ambient temperature, wide electrochemical window (4.7 V), and favorable mechanical strength (25 MPa). The Na_3_V_2_(PO_4_)_3_/MoS_2_ sodium-ion full cell using this polymer electrolyte delivered 88 mAh g^−1^ at 0.5 C, with 84% capacity retention after 1000 cycles.

Gao et al. proposed a gel-polymer/glass-fiber electrolyte consisting of PVDF–HFP reinforced by a glass-fiber paper and modified by a polydopamine coating to tune the surface properties and then saturated with a solution of 1 mol L^−1^ NaClO_4_ in PC. The sodium half-cell with this electrolyte and Na_2_MnFe(CN)_6_ as the cathode delivered an initial discharge capacity of 120 mAh g^−1^, with a capacity retention of 89.4% after 100 cycles at 1C [534].

A symmetrical sodium cell was cycled at 50 °C with an electrolyte composed of trimethylisobutylphosphonium (P_111i4_) TFSI organic ionic plastic crystal and a high concentration of NATFSI [598]. For the optimized composition of 25 mol% P_111i4_-TFSI-75 mol% NaTFSI, a stability of 100 cycles at 0.1 mA cm^−2^ was observed, along with good compatibility with sodium. At the same temperature, where this electrolyte is almost a solid, the NaFePO_4_ cell cycled at C/10 delivered 75 mAh g^−1^, but only five cycles were tested.

Among solid oxide electrolytes, Na-β”-alumina has been the subject of many studies, which have been reviewed in particular in reference [599], because of its high conductivity (2 × 10^−3^ S cm^−1^ at 25 °C). However, it is difficult to prepare and mixed with another phase Na-β-alumina, which is less conductive. Therefore, a high-enough β”/β ratio is needed, and, for this purpose, different metal oxides (MgO, Li_2_O) are added to stabilize the β” phase. Some progress has been made in recent years [600,601]. However, too much of the β” phase is not desirable either, because of its poor mechanical strength and sensitivity to moisture [602]. The other difficulty already mentioned regarding the use of solid electrolytes in lithium batteries is the need to minimize the amount of grain boundaries, and progress has been achieved in the particular case of Na-β”-Al_2_O_3_ [603]. The second difficulty, also mentioned earlier, is to maintain the contact between the solid-state electrolyte and electrodes. We have shown that this is usually addressed by using a composite, in particular, a polymeric buffer layer, or by adding a thin layer of liquid between the electrode and the solid pellet. Another solution was proposed by Liu et al. [604], who designed a “toothpaste cathode” consisting of a mixture of PY_14_-FSI ionic liquid with Na_0.66_Ni_0.33_Mn_0.67_O_2_ active particles and Super-P, with the fraction of active material being 40 wt.%. Here, the ionic liquid acts as the wetting agent, binder, and ionic conductor, while Super-P carbon is employed here to ensure the electrical conductivity and provide the paste appearance. The cell with this cathode, Na-β”-Al_2_O_3_ solid electrolyte, and sodium-metal anode at 70 °C delivered capacities of 80 and 58 mAh g^−1^ at 0.1C and 6C, respectively, for an active material loading of 2 mg cm^−2^. The cycling stability was outstanding, with the capacity retention being 90% after 10 000 cycles at 6C and the Coulombic efficiency being close to 100%.

Closo-boranes are also considered as promising electrolytes for all-solid-state sodium batteries. Na_2_(B_12_H_12_)_0.5_(B_10_H_10_)_0.5_ exhibits a conductivity of 0.9 × 10^−3^ S cm^−1^ at 20 °C and is compatible with sodium metal, with a stability window of 3 V [605]. Substitution of the closo-anion of Na_2_B_12_H_12_ enhances the ionic conductivity; in particular, a conductivity of 10^−1^ S cm^−1^ at 360 K was obtained with Na_2_B_12_H_12−x_I_x_ [606]. Hansen et al. demonstrated that the [B_12_*X*_12_]^2−^ (*X* = F, Cl, Br…) of halogenated sodium-closo-dodecaboranes undergo reorientational motions at high temperatures that promote the conductivity of the Na^+^ [607].

Sodium-rich antiperovskites like Na_3_OBr and Na_4_OI_2_ also show promise owing to their high Na^+^ conductivities, and the reasons have been explored by Zhu et al. [608]. Such antiperovskite-based electrolytes, however, still need to be tested, and conductivity is not the only parameter to be considered. For instance, Li_3_OCl antiperovskite films in lithium batteries display a good conductivity of 2 × 10^−4^ S cm^−1^, but a cell with this electrolyte and LiCoO_2_ cathode that was tested at the current density of 10 mA g^−1^ between 2.2 and 4.2 V delivered a capacity that already decreased to 64 mAh g^−1^ after 20 cycles [179].

Another strategy is to obtain polymer electrolytes that are suited to sodium cells, so that the ions are mobile even below the glass transition temperature, in which case the segmental motion of the polymer is decoupled from the ionic conductivity. This can be achieved by replacing the small Na^+^ by the bulky quaternary ammonium cations. The chemical structures of such polymers can be found in reference [257]. Noor et al. selected poly(2-acrylamido-2-methyl-1-propane-sulfonate) (PAMPS) homopolymer [609] or PAMPS with polyvinyl sulfonate [610] as a polymer backbone. With the addition of a small amount of a tetraglyme plasticizer, the conductivity reached 10^−5^ S cm^−1^ at 50 °C; however, tetraglyme displays no effect on the Li^+^ dynamics in PAMPS-based ionomers, the reasons for which have been explained by molecular dynamics calculations [611,612,613]. Usually, these polymers are tested in Na/Na symmetric cells to verify their compatibility with sodium. However, such polymers need to be tested in sodium half-cells. When using NaFePO_4_, such a cell delivered only 80 mAh g^−1^ at C/5 at 70 °C [535].

We have already mentioned the interest in using phosphonium FSI ionic liquid electrolyte for lithium batteries [204]. The same group also investigated this electrolyte with a high salt concentration for sodium-ion batteries [614,615]. In particular, a Na//NaFePO_4_ cell with P_111i4_FSI ionic liquid and NaFSI (sodium salt) as the electrolyte delivered a capacity of 85 mAh g^−1^ at C/2 at 50 °C, with a capacity retention of 95% after 100 cycles. Recently, this group further fabricated an ion gel membrane based on 50 wt.% poly(DADMA)-TFSI (a PIL that is already used in lithium batteries [306]) and 50 wt.% C3mpyrFSI, to which was added 14 mol% NaFSI salt plus 5 wt.% Al_2_O_3_ nanoparticles to improve the mechanical properties [616]. 50 wt.% C3mpyrFSI was the optimum concentration, as a higher concentration, despite increasing the transference number, decreases the ionic conductivity. This composite was tested as an electrolyte in a Na//NaPO_4_ cell. At 70 °C, the cell cycled from 1.5 to 4.0 V delivered a capacity of 115 mAh g^−1^ at C/20 and 85 mAh g^−1^ at C/5, and was able to remain stable for 60 cycles, though the capacity reduced to 90 mAh g^−1^ at C/20. Nevertheless, this is the first solid-state sodium battery based on ion gels, and, in this sense, the result is promising, as it is already better than the capacities obtained with polymers.

## 8. Concluding Remarks

The research domain dealing with electrochemical energy storage is one of the most active in science and almost all countries are extensively funding scientists and institutes, not to forget the internal efforts of large companies. The pace is even more frantic that the forecast, if climate action is not tackled, in particular with transportation and renewable energy storage, is dire. By providing and discussing ≈ 600 references, the authors hope to guide the reader to a heap of results that reflect the ingenuity of the scientific community.

The three types of electrolytes, liquids, including ionic liquids, polymers per se or as gelling agents for immobilized liquids, and ceramics, are all contenders, with their own pros and cons, and are often used in conjugation. The conductivities of dry polymer electrolytes, either PEO-based or now polyester, can be up to 10^−4^ S cm^−1^ at room temperature, but they are still mostly used at 50–70 °C to minimize the diffusion and transfer resistances. Polyesters show some chemical fragility on the metal electrode side and the degradation products can act as plasticizers, thereby artificially enhancing the performance of batteries [617,618,619].

Gels, with infinite choices of scaffolding polymers, are able to trap a large fraction of liquids (organic or ionic liquids), and the conductivity almost reaches that of the free solvent, being up to almost 10^−2^ S cm^−1^ at room temperature. The organic solvent thus becomes responsible for the development of the SEI with the electrode, but the interfacial resistance is low. Interestingly, gels, together with dry polymers, allow tethering of negative charge to the backbone, which results in transference numbers close to unity. Multilayer electrolytes are often efficient in preventing the crosstalk between electrodes, as in the case of polysulfides.

Ceramics are often considered as the ultimate solution to the electrolyte problems. It is true that a number of materials are now known with conductivities of the order of 10^−3^ S cm^−1^ at room temperature. However, the difficulties in processing ceramics cannot be overcome, as a 1 kWh battery, whatever be the electrolyte, requires several square meters of surface. Sintering processes do not result in thicknesses well below 30 µm and, for garnet electrolytes, require high temperatures. Garnets are in addition inhibited by their high specific gravity, which is reflected in the energy density of the cell. All the deposition techniques of complex materials that involve vacuum (sputtering, etc.) are of low productivity and thus expensive. In this respect, sulfide-based electrolytes stand out, as they can be simply pressed into shape for the electrolyte or composite electrode. The interfacial contact is often established by the insertion of a layer of polymer/gel or liquid (ionic liquid).

Certainly, the composite electrolytes combining an inorganic ceramic and a solvating polymer are the object of intense research activity. It is hoped that they will combine the best of the two worlds by benefitting from the high conductivity of ceramics and the processability of polymers. The results so far acquired are promising and point toward the need to have nanoparticle dispersions in the macromolecule, with possible interactions on both sides of the interface, in particular, creation of shallow vacancies on the ceramic side, which enhances the conductivity. However, great care must be taken, as most of the ceramic materials are very sensitive to moisture, and the effect is multiplied by the surface area.

It is very difficult to point to a possible watershed technology and several more years will be needed to see the emergence of possible winners. The investments in setting-up a disruptive technology are staggering at the scale of the EV/grid storage market, and industrialists may be hesitant, considering the amount that they have already invested in scaling-up the conventional technology. However, lithium-metal polymer technology already uses alternative manufacturing process such as, for instance, the highly efficient extrusion, instead of the low productivity solvent casting method, and has proven to be competitive at its scale (≈2 MWh/yr).

What is certain is that the pace of discovery will not slow and the authors hope that more collaborations between research teams, countries, and continents will be forged, considering the urgency to find sustainable ways to store the 10^12^ kWh/yr that is needed for mankind.
